# Diet of Mesozoic toothed birds (Longipterygidae) inferred from quantitative analysis of extant avian diet proxies

**DOI:** 10.1186/s12915-022-01294-3

**Published:** 2022-05-12

**Authors:** Case Vincent Miller, Michael Pittman, Xiaoli Wang, Xiaoting Zheng, Jen A. Bright

**Affiliations:** 1 Department of Earth Sciences, The University of Hong Kong, Pokfulam, Hong Kong SAR, China; 2 School of Life Sciences, The Chinese University of Hong Kong, Shatin, New Territories, Hong Kong SAR, China; 3 Department of Earth Sciences, University College London, Gower Street, London, WC1E 6BT UK; 4 Institute of Geology and Paleontology, Linyi University, Linyi City, Shandong 276005 China; 5 Shandong Tianyu Museum of Nature, Pingyi, Shandong 273300 China; 6 Department of Biological and Marine Sciences, University of Hull, Hull, HU6 7RX UK

**Keywords:** Aves, Avialae, Birds, Diet, Cretaceous, Finite element analysis, Body mass, Morphometrics, Mechanical advantage, Jehol Biota

## Abstract

**Background:**

Birds are key indicator species in extant ecosystems, and thus we would expect extinct birds to provide insights into the nature of ancient ecosystems. However, many aspects of extinct bird ecology, particularly their diet, remain obscure. One group of particular interest is the bizarre toothed and long-snouted longipterygid birds. Longipterygidae is the most well-understood family of enantiornithine birds, the dominant birds of the Cretaceous period. However, as with most Mesozoic birds, their diet remains entirely speculative.

**Results:**

To improve our understanding of longipterygids, we investigated four proxies in extant birds to determine diagnostic traits for birds with a given diet: body mass, claw morphometrics, jaw mechanical advantage, and jaw strength via finite element analysis. Body mass of birds tended to correspond to the size of their main food source, with both carnivores and herbivores splitting into two subsets by mass: invertivores or vertivores for carnivores, and granivores + nectarivores or folivores + frugivores for herbivores. Using claw morphometrics, we successfully distinguished ground birds, non-raptorial perching birds, and raptorial birds from one another. We were unable to replicate past results isolating subtypes of raptorial behaviour. Mechanical advantage was able to distinguish herbivorous diets with particularly high values of functional indices, and so is useful for identifying these specific diets in fossil taxa, but overall did a poor job of reflecting diet. Finite element analysis effectively separated birds with hard and/or tough diets from those eating foods which are neither, though could not distinguish hard and tough diets from one another. We reconstructed each of these proxies in longipterygids as well, and after synthesising the four lines of evidence, we find all members of the family but *Shengjingornis* (whose diet remains inconclusive) most likely to be invertivores or generalist feeders, with raptorial behaviour likely in *Longipteryx* and *Rapaxavis.*

**Conclusions:**

This study provides a 20% increase in quantitatively supported fossil bird diets, triples the number of diets reconstructed in enantiornithine species, and serves as an important first step in quantitatively investigating the origins of the trophic diversity of living birds. These findings are consistent with past hypotheses that Mesozoic birds occupied low trophic levels.

**Supplementary Information:**

The online version contains supplementary material available at 10.1186/s12915-022-01294-3.

## Background

The diet of most non-avian avialans has been largely speculative so far [1–3]. We use Aves in this paper to refer to crown group birds, and Avialae to refer to crown group birds plus all coelurosaurian theropods closer to them than to either dromaeosaurids or troodontids [4]. Among Enantiornithes, the most diverse and widespread avialans in the Mesozoic, only *Eoalulavis* and *Shenqiornis* have good evidence (i.e. fossilised digestive tract contents or more than one line of quantitative proxy evidence) backing any particular diet [1, 5] out of nearly 100 known species [6]. A more robust understanding of non-avian avialan diet will allow us to test key hypotheses in bird evolution, such as beak evolution allowing for extant bird dietary diversity [7, 8], trophic level reduction driving powered flight development [9], and birds occupying a low-level consumer role in Mesozoic ecosystems [10].

**Fig. 1:**
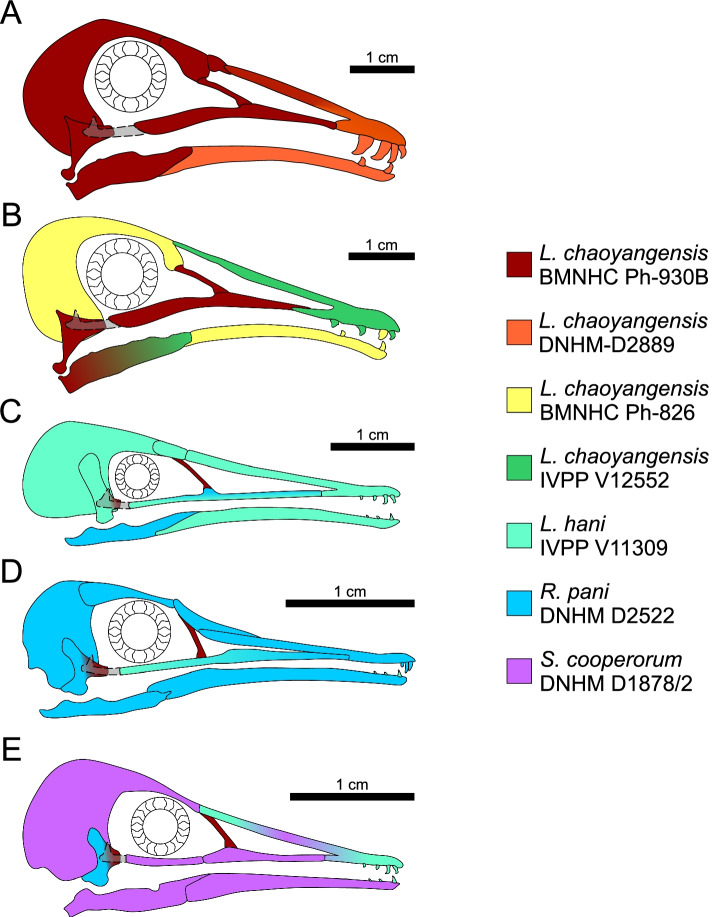
Reconstructions of longipterygid skulls. Reconstructions are of *Longipteryx* morphotypes with large teeth (**A**) and small teeth (**B**), *Longirostravis* (**C**), *Rapaxavis* (**D**), and *Shanweiniao* (**E**). Colours of different bones indicate which specimen that bone is based on. All sclerotic rings are based on BMNHC Ph-930B. Quadratojugal morphology is unknown in any taxon so is drawn in a dotted line. See the “Methods” section for more details on reconstruction. Scale bars are based on DNHM-D2889 (**A**), IVPP V12552 (**B**), IVPP V11309 (**C**), DNHM D2522 (**D**), and DNHM D1878/2 (**E**)

Longipterygidae is a consistently recovered clade within Enantiornithes [11] comprising six genera [11–14] from the Yixian and Jiufotang formations (≈125–120 Ma) [11] of the Jehol Group of north-eastern China. The most conspicuous features of longipterygids are their elongate rostra and rostrally restricted dentition [12]. *Longipteryx* has been suggested to have fed on fish in reference to its large and recurved teeth and its more robust rostrum than other longipterygids [15, 16]. The implied analogy seems to be to longirostrine crocodilians like gharials (*Gavialis gangeticus*) and false gharials (*Tomistoma schlegelii*). *Longirostravis*, *Rapaxavis* and *Shanweiniao* have been proposed as probe feeders (in mud [11, 15] and tree bark [17]) on the basis of their elongate rostra in analogy to those of oystercatchers (*Haematopus*) and shanks (*Tringa*) [15, 17]. Longipterygids preserve more complete skulls than any other enantiornithine family [1], providing the best opportunity for family-level inferences for reconstruction of enantiornithine skull morphology (Fig. 1). With existing hypotheses of diet to test and well-preserved fossils to work with, Longipterygidae is the ideal starting point for investigating enantiornithine diet in detail.

In this study, we investigate the diet of nearly all longipterygid genera: *Longipteryx*, *Longirostravis*, *Rapaxavis*, *Shanweiniao* and *Shengjingornis*. Only *Boluochia* is excluded, due to its poor preservation [11, 13]. We also note two distinct morphotypes within the genus *Longipteryx* and analyse each separately: one morphotype with large teeth and a more robust skull and another with small teeth and a more gracile skull (Fig. 1A, B). We investigate the ecology of these fossil birds using data from four lines of evidence: body mass estimation, traditional morphometrics (TM) of pedal unguals, mechanical advantage (MA) and functional index analysis of upper jaws, and finite element analysis (FEA) of the lower jaws [1].

**Fig. 2:**
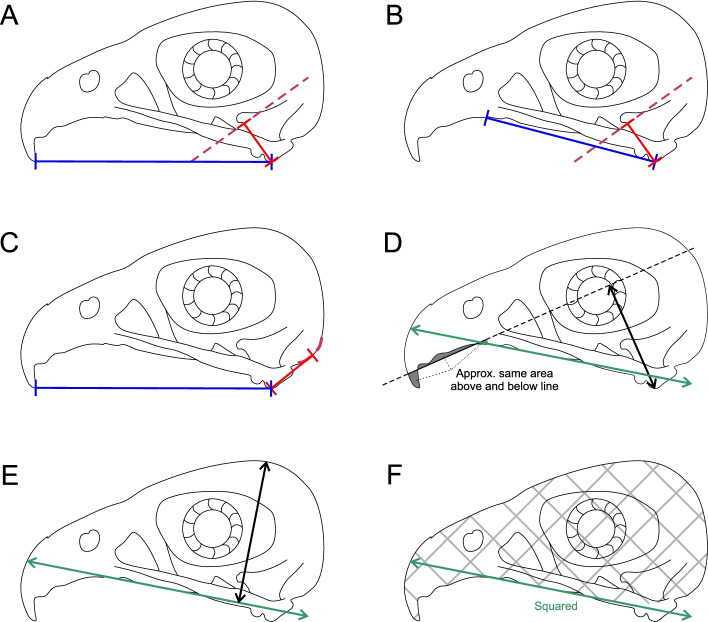
Measurements taken to calculate mechanical advantage and functional indices in this study. All are mapped onto the outline of a skull of *Falco peregrinus*. Measurements are of anterior jaw-opening mechanical advantage AMA (**A**), posterior jaw-opening mechanical advantage PMA (**B**), jaw-opening mechanical advantage OMA (**C**), relative articular offset AO (**D**), relative maximum cranial height MCH (**E**), and relative average cranial height ACH (**F**). Outlevers are drawn in blue, inlevers in red, and skull length in green. Lines of action of *m. adductor mandibulae* (**A**, **B**) and attachment of *m. depressor mandibulae* (**C**) are indicated by a dashed pink line. The crosshatched region in **F** indicates an area measurement. Line drawing based on specimen CM S-14309

Body mass has been found in recent studies [18, 19] to be an effective predictor of bird diet, with developmental [20] or mechanical [1] constraints proposed as explanations. For extinct taxa, mass can be estimated with high accuracy from limb bone measurements [21]. TM is quantitative analysis of measurements believed to be of ecological importance [22]. In this case, TM has proven effective at distinguishing the claws of raptorial birds from non-raptors [23–26] and different styles of raptorial predation from one another [24, 27] based on claw size and curvature. Functional indices are ratio measurements of an animal’s morphology that inform its mechanical properties. The classic functional index measured is MA, treating the jaw as a class 3 lever and seeing if it is adapted to move at high speeds (low MA) or with high force (high MA) [28–30]. Three versions of MA and three other functional indices for the upper jaw have previously been shown effective at discerning diet in extant animals [1, 31], all diagrammed in Fig. 2. FEA is a modelling technique used to simulate how objects respond to a force [32]. In this paper, we investigate how the lower jaws of birds respond to forces generated during a bite. We utilise mesh-weighted arithmetic mean (MWAM) strain [33] for summary statistics of models and the intervals method [34] to quantitatively compare size-scaled FEA models in a more detailed manner. The intervals method splits the range of strain within models into equal intervals and quantifies the percentage of model area under each interval of strain, and these intervals are then subjected to multivariate analysis.

In order to place the results of these analyses into proper ecological context, we gathered data from over 170 extant bird species across 13 diets and 7 pedal ecological categories. Phylogeny is expected to have a major influence on both diet [19, 35] and pedal ecology [25, 36], so we quantify the effect of phylogeny with the *K*_mult_ statistic [37] and test for differences between categories using phylogenetic honest significant differences (HSD; an alternative to repeated ANOVA more appropriate for a large number of categories [38, 39]). Diet categories are based on EltonTraits 1.0 [40], a database of quantitative diet information for bird and mammal species. However, diet categorisation is not simple, as traditional categories will often include both animals that feed exclusively on their primary food source and animals which supplement their primary food source with many other foods [41]. So, in choosing cut-offs for our diet categories (Table 1), we chose to primarily study birds which fed near-exclusively on their primary food source to make trends as clear as possible. We also include secondary analyses including birds whose diets were worse representatives of their category (i.e. less specialised) but increased the phylogenetic breath included in the study, which we dub “semi-specialists”, to see if observed trends were robust to their inclusion. This is particularly important as some specialist diets for birds (e.g. nectarivory) cover very little phylogenetic breadth [35] and past studies of bird diet have found trends to weaken as phylogenetic breadth increased [18, 29, 36, 42]. We also subdivide some diet categories where food has a large range of mechanical properties, see “Methods” for details. TM studies of bird claws [24, 25] have not proven effective at discriminating specific diets, but rather show more general instances of raptorial use of the foot (hereafter “pedal ecology”). Raptorial adaptations would imply some level of carnivory in the diet, and claw data may provide information on prey size [43] or hunting strategies [24]. Following [24], we distinguish between ground birds and perching birds based on their general place of residence and four types of raptorial predation: talons adapted for piercing, restraining, striking, or suffocating prey. Scavenging birds are grouped separately as past studies have been inconsistent in classifying them as ground [24] or perching [43] birds.

**Table 1:** Cut-offs for diets used in this study. Percentages refer to values given in EltonTraits 1.0, with Diet-Tetr being the sum of Diet-Ect and Diet-End (i.e. ectothermic and endothermic tetrapods are combined). Semi-specialists refer to birds that are less strongly aligned with a given diet but greatly increase the taxonomic breadth of the sample for that diet

Diet	Standard Cut-Off	Semi-specialist Cut-Off
Folivore	80+% Diet-PlantO	60+% Diet-PlantO
Frugivore	80+% Diet-Fruit	60+% Diet-Fruit
Generalist	30% or less in any category	40% or less in any category
Granivore	90+% Diet-Seed	70+% Diet-Seed
Invertivore	90+% Diet-Inv	60+% Diet-Inv
Nectarivore	80+% Diet-Nect	60+% Diet-Nect
Piscivore	70+% Diet-Fish	50+% Diet-Fish
Scavenger	100% Diet-Scav	50+% Diet-Scav
Tetrapod Hunter	80+% Diet-Tetr	60+% Diet-Tetr

Once data are gathered, analysed and interpreted for each line of evidence, their interpretations are then synthesised into a final diet assignment for the fossil taxa. Each line of evidence can support or rule out various diets, but by synthesising the four lines of evidence together, we can arrive at more precise inferences. We use this framework to test the current hypotheses of specialised invertivory and piscivory in Longipterygidae. In the course of this study, we both refine the dietary categories among extant birds and collect new ecomorphological and ecophysiological data, which in turn refines our understanding of extant ecosystems as well as extinct ones. This study thus provides a template for future studies of avian ecomorphology.

## Results

### Body mass

**Fig. 3:**
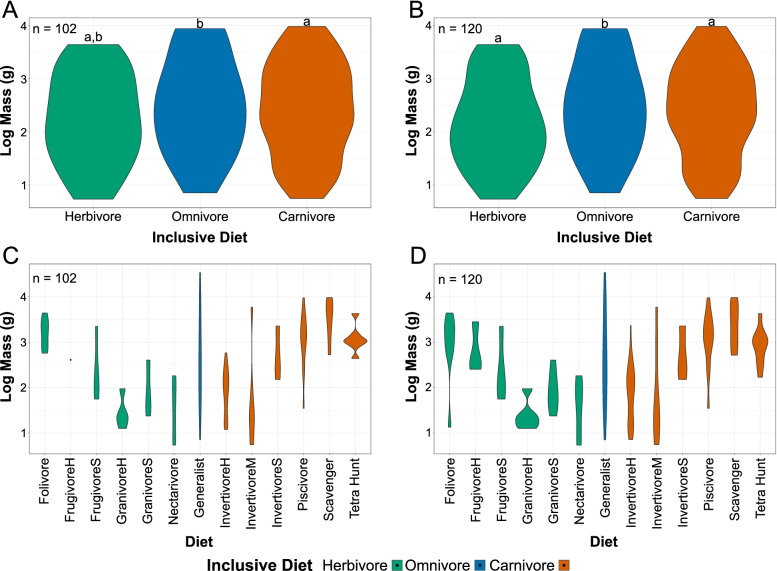
Violin plots of bird body mass, organised by more inclusive diets and the whole range of diets considered. **A**, **B** Bird masses grouped by broad categories of diet, “inclusive diet”, excluding (**A**) and including (**B**) semi-specialists. **C**, **D** Bird masses grouped by the main diet categories in this paper, excluding (**C**) and including (**D**) semi-specialists. In **C**, FrugivoreH is represented by a single taxon, thus is a point. *Dromaius novaehollandiae* is excluded from all graphs as an outlier. Inclusive diets with the same letters above them do not have significantly different average masses in phylogenetic HSD at the *p* = 0.05 level (Table 2). Diet abbreviations: FrugivoreH hard frugivore, FrugivoreS soft frugivore, GranivoreS swallowing granivore, GranivoreH husking granivore, InvertivoreH hard invertivore, InvertivoreM medium invertivore, InvertivoreS soft invertivore, Tetra Hunt tetrapod hunter

Violin plots of body masses organised by diet are provided in Fig. 3. Table 2 provides *p*-values testing if diet means are significantly different with Tukey’s HSD [38] and phylogenetic HSD [39]. Masses of included taxa range from 5 g in the hummingbird *Phaethornis yaruqui* to 34,200 g in the ratite *Dromaius novaehollandiae*, though 70% of taxa are less massive than 1000 g. More inclusive diets (i.e. carnivore, herbivore, omnivore) have similar mass distributions when viewed as a gestalt (Fig. 3A, B). Tukey’s HSD found average masses of more inclusive diets to not be significantly different, though phylogenetic HSD found the mean masses of carnivores and omnivores to be significantly different at the *p* < 0.01 level. While significantly different, the effect size appears to be very small because their mass distributions are nearly identical (Fig. 3A, B). When broken into more precise diets (Fig. 3C, D), categories become more distinct. Granivores, nectarivores, and medium and hard invertivores tend towards smaller body masses (5–5908 g, $\overline{\mathrm{x}}$ = 58 g), whereas folivores, frugivores, soft invertivores, piscivores, tetrapod hunters, and scavengers tend towards larger body masses (35–9625g, $\overline{\mathrm{x}}$ = 1089 g). Generalists occupy the full range of body masses observed roughly evenly (7–8786 g, $\overline{\mathrm{x}}$ = 336 g with *Dromaius* excluded as an outlier at 34,200 g).

**Table 2:** *P*-values for Tukey’s HSD and phylogenetic HSD testing whether the mean mass of birds is different in different among inclusive diets (i.e. combinations of those in Table 1). *p*-values are indicated with one asterisk (*) for significance at the 0.05 level, two at the 0.01 level, and three at the 0.001 level. Diet abbreviations: FolFrug Folivore + Frugivore, GranNect Granivore + Nectarivore

	Comparison	Tukey’s ***p***	Phylogenetic ***p***
**No semi-specialists**	Carnivore vs Herbivore	6.99E−01	8.50E−02
	Carnivore vs Omnivore	9.40E−01	3.00E−03**
	Herbivore vs Omnivore	6.46E−01	8.10E−02
	Vertivore vs Invertivore	1.27E−10***	1.00E−03***
	FolFrug vs GranNect	3.23E−05***	3.60E−02*
**All birds**	Carnivore vs Herbivore	4.36E−01	7.20E−02
	Carnivore vs Omnivore	9.14E−01	1.00E−03***
	Herbivore vs Omnivore	4.44E−01	2.80E−02*
	Vertivore vs Invertivore	1.62E−10***	1.00E−03***
	FolFrug vs GranNect	2.35E−05***	2.00E−03**

**Fig. 4:**
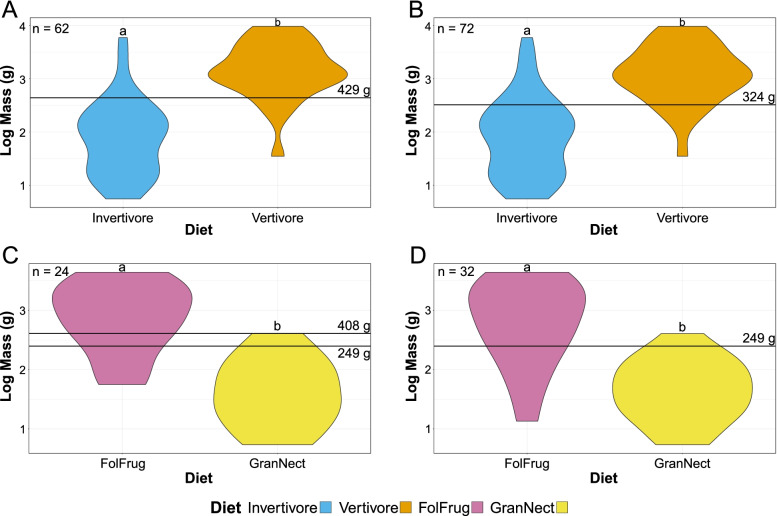
Violin plots of bird body mass, by diet. Masses of carnivores (**A**, **B**) and herbivores (**C**, **D**) are grouped by trends apparent in Fig. 3C, D, excluding (**A**, **C**) and including (**B**, **D**) semi-specialists. Carnivores are split into invertivores and vertivores, herbivores are split into folivores + frugivores (FolFrug) and granivores + nectarivores (GranNect). Cut-off points, calculated using the Youden index (see “Methods”), are labelled with a line. Diets with the same letters above them do not have significantly different average masses in phylogenetic HSD at the *p* = 0.05 level (Table 2)

Among carnivores (Fig. 4A, B), vertivore (piscivore, scavenger, and tetrapod hunter), body mass (35–9625 g, $\overline{\mathrm{x}}$ = 1426 g) tends to be greater than invertivore body mass (6–5908 g, $\overline{\mathrm{x}}$ = 75 g). Tukey’s HSD and phylogenetic HSD find mean masses of invertivores and vertivores to be significantly different at the *p* < 0.01 level with and without semi-specialists. Optimising the Youden Index (a summary measure commonly used to select cut-off points in medicinal diagnostics [44]), mass cut-off points between vertivores and invertivores are calculated at 324 g for all carnivorous birds and at 439 g for carnivorous birds excluding semi-specialists.

Among herbivores (Fig. 4C, D), body mass of folivores (575–4400 g, $\overline{\mathrm{x}}$ =1537 g) and frugivores (56–2333 g, $\overline{\mathrm{x}}$ = 259 g; FolFrug $\overline{\mathrm{x}}$ = 732 g) tends to be greater than that of granivores (13–406 g, $\overline{\mathrm{x}}$ = 45 g) and nectarivores (5–183 g, $\overline{\mathrm{x}}$ = 36 g; GranNect $\overline{\mathrm{x}}$ = 41 g). Tukey’s HSD finds mean masses of folivores and frugivores to be significantly different from granivores and nectarivores at the *p* < 0.01 including and excluding semi-specialists. These results are maintained with phylogenetic HSD, but only at the *p* < 0.05 level. Optimising the Youden Index, the mass cut-off point between folivores + frugivores and granivores + nectarivores is calculated at 249 g for all herbivorous birds. Two cut-off points optimise the Youden Index when excluding herbivorous semi-specialists, 249 g and 408 g.

Statistically significant phylogenetic signal is present in all mass datasets except all diets with semi-specialists included (Table 3). *K* is a univariate statistic measuring the phylogenetic signal relative to a Brownian motion model [45]. Across all diets, *K* is 0.94 when semi-specialists are excluded and 0.42 with semi-specialists included. For carnivores, *K* is 1.49 when semi-specialists are excluded and 1.03 with semi-specialists included. For herbivores, *K* is 1.62 when semi-specialists are excluded and 1.24 with semi-specialists included (Table 3). *Dromaius* is excluded from these calculations as an outlier; it greatly increases the *K* value when included.

**Table 3:** *K*_mult_ [37] values for all datasets analysed in this paper. Values for body mass are technically *K* values but are calculated and interpreted identically. *K*_mult_ = 1.0 indicates a similarity of measured traits expected if traits evolved under Brownian motion, values less than 1 indicate traits more different than expected from Brownian motion and values greater than 1 indicate traits more similar than expected from Brownian motion [45]. *p*-values are indicated with one asterisk (*) for significance at the 0.05 level, two at the 0.01 level, and three at the 0.001 level. Significant *p*-values indicate the presence of phylogenetic signal. Note that the *K*_mult_ function in [37] places a lower limit on the returned *p*-value, so *p*-values reported as 1.00E−03 may be more significant

	***K***_**mult**_	***P***-value
**Mass no semi-specialists**	0.9416885	1.00E−03***
**Mass all birds**	0.4154562	6.70E−02
**Mass carnivores no semi-specialists**	1.409133	1.00E−03***
**Mass all carnivores**	1.029493	1.00E−03***
**Mass herbivores no semi-specialists**	1.616050	1.00E−03***
**Mass all herbivores**	1.236025	1.00E−03***
**TM**	0.6574164	1.00E−03***
**MA no semi-specialists**	0.8710608	1.00E−03***
**MA all birds**	0.7924732	1.00E−03***
**FEA no semi-specialists**	0.4264590	1.21E−01
**FEA all birds**	0.3151973	2.26E−01

Inclusion of semi-specialists most strongly affects the distribution of folivores and tetrapod hunters. The lower extreme of folivore mass is decreased an order of magnitude solely by the inclusion of the semi-specialist pygmy parrot *Micropsitta bruijnii* (Fig. 3B). No folivorous taxa have masses between those of *M. bruijnii* (14g) and *Brotogeris cyanoptera* (56g). *M. bruijnii* likewise is the sole driver of the decreased minimum mass of FolFrug herbivores when excluding semi-specialists (Fig. 4C). Tetrapod hunters appear to have a trimodal distribution when semi-specialists are excluded (Fig. 3B), but this is an artefact of the abundance of taxa with masses near 1 kg. Tetrapod hunter semi-specialists are all below this mass and still unify the entire dataset to a semi-normal distribution (Fig. 3A).

### Traditional morphometrics

TM data are categorised based on use of the talons, in an attempt to identify raptorial behaviour, rather than diet. To ensure parallax was not affecting the inferences from our results, we took multiple photographs at different angles (see “Methods”) and found a range of 0.87° in angle measured from photographs (the standard deviation of all claw angles is 18.73°). Pictures of in-focus grid lines remained orthogonal, so measured image distortion was negligible.

**Fig. 5:**
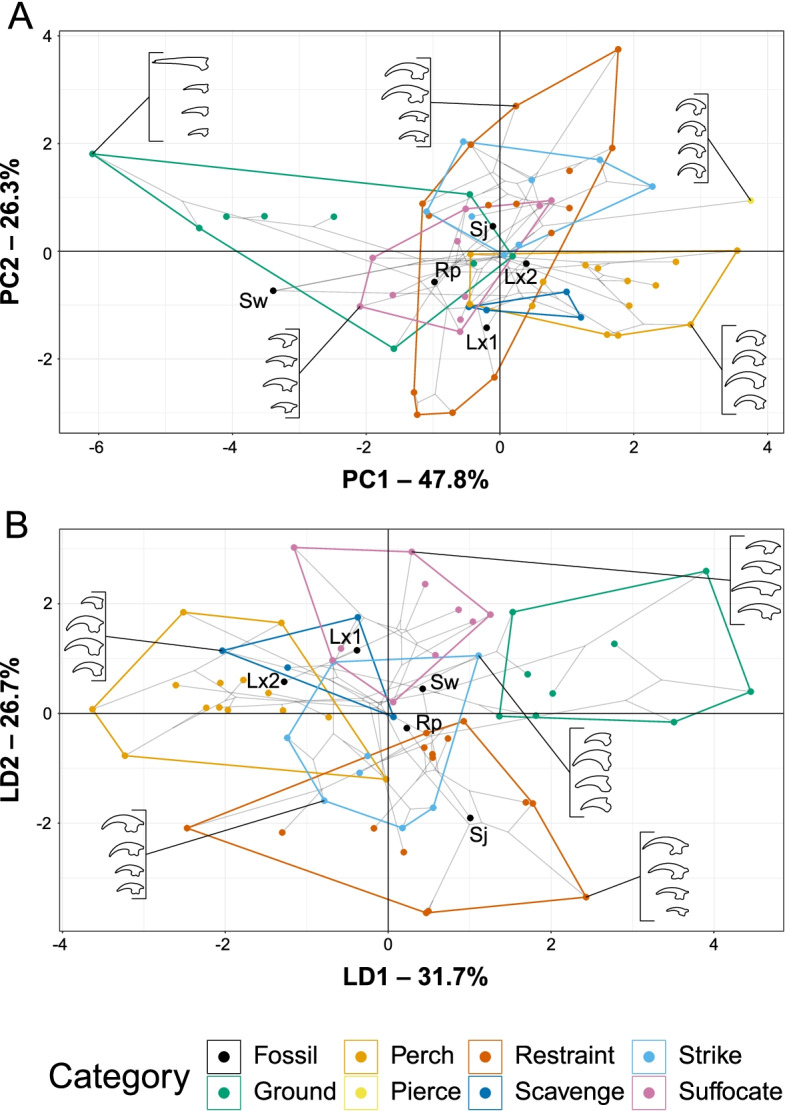
Phylomorphospace of avian and longipterygid unguals, based on traditional morphometrics, grouped by pedal ecology. Grey lines indicate phylogenetic relationships. Line drawings of claws for selected taxa are provided for reference. Data is visualised with PCA (**A**) and LDA (**B**). In PCA (**A**), PC1 describes talon curvature and PC2 describes interdigital size variation. In LDA (**B**), LD1 describes the size ratio of digits II and IV to digit III and LD2 describes the size ratio of digits I and IV to digit III. See Additional file 1: Fig. S1 for precise character loadings. Taxon abbreviations: Lx1 *Longipteryx chaoyangensis*, Lx2 *Longipteryx* sp., Rp *Rapaxavis pani*, Sw *Shanweiniao cooperorum*, Sj *Shenjingornis yangi*

A principal component analysis (PCA) plot of TM data is provided in Fig. 5A with character weights plotted in Additional file 1: Fig. S1A. An interactive graph is available in Additional file 2. PC1 and PC2 explain 74.1% of the total variance. PC1 is driven by the curvature of claws from all four digits. PC2 is driven primarily by the size ratio of DI and DII to DIII with a lesser contribution of the size ratio of DIV to DIII. Groups are generally tightly clustered in PCA, but restraint raptors and ground birds are less so. Restraint raptors form two distinct groups: hawks and eagles plus the forest-falcon *Micrastur semitorquatus* and the helmetshrike *Prionops plumatus* (weakly positive PC1, strongly positive PC2) contrasting with the true shrikes (Laniidae; weakly negative PC1, strongly negative PC2). Ground birds form two clusters: larks and megapodes (strongly negative PC1, moderately positive PC2) versus tinamous and the crane *Balearica pavonina* (clustered near the origin) with the crane *Grus canadensis* as an outlier (weakly negative PC1, moderately negative PC2). While less spread than restraint or ground categories, the suffocate group also forms three distinct clusters: one near restraint and striking groups (made up of the owls *Bubo virginianus*, *Surnia ulula*, *Pulsatrix perspicillata*, and *Ninox novaeseelandiae*), one near perching birds (made up of the owls *Athene brama*, *Glaucidium cuculoides*, and *Strix varia*), and one near the crane *Grus canadensis* (made up of the owls *Strix aluco*, *Tyto alba*, and *Asio otus*). Most longipterygids plot near the origin, a region with representatives from every pedal ecological category except for scavengers and piercing raptors. *Longipteryx chaoyangensis* plots in a more negative region of PC2 closer to scavengers and farther from restraining and striking raptors. *Shanweiniao cooperorum* has claws which plot far from the origin in highly negative PC1, a region inhabited only by ground birds.

A linear discriminant analysis (LDA) plot of TM data is provided in Fig. 5B with character weights plotted in Additional file 1: Fig. S1B. An interactive graph is available in Additional file 3. LD1 and LD2 explain 58.4% of the total variance. LD1 is driven by the size ratio of DII to DIII in the positive direction and the size ratio of DIV to DIII in the negative direction. LD2 is driven by the size ratio of DIV to DIII in the positive direction and size ratio of DI and DII to DIII in the negative direction. Angular measures always have little effect on linear discriminants relative to size ratios, though their effect generally increases at higher (i.e. less influential overall) LDs. Ground birds completely separate in the morphospace (highly positive LD1), and restraint (moderately negative PC2), suffocate (moderately positive PC2), and perching (moderately negative PC1) birds are mostly separate from one another. Scavengers overlap perching and suffocating birds, and striking birds overlap all groups but ground birds. *Rapaxavis pani* and *Shanweiniao cooperorum* plot near the origin. *Longipteryx chaoyangensis* and *Longipteryx* sp. both plot at moderately negative PC1 and weakly positive PC2. *Shengjingornis yangi* plots at moderately positive PC1 and moderately negative PC2. Discriminant predictions (Table 4) find *L*. *chaoyangensis*, *Longipteryx* sp., and *R*. *pani* most likely to be suffocating raptors, though *R*. *pani* also has a notable affinity with ground birds. *S*. *cooperorum* has nearly equal affinity with ground birds and suffocating raptors while *S*. *yangi* was most likely a ground bird. Discriminant analysis of principal components (DAPC; used as a check on the robustness of LDA to broken assumptions—see “Methods”) [46] (Additional file 1: Fig. S2A) and its predictions (Table 4), are consistent with LDA.

**Table 4:**
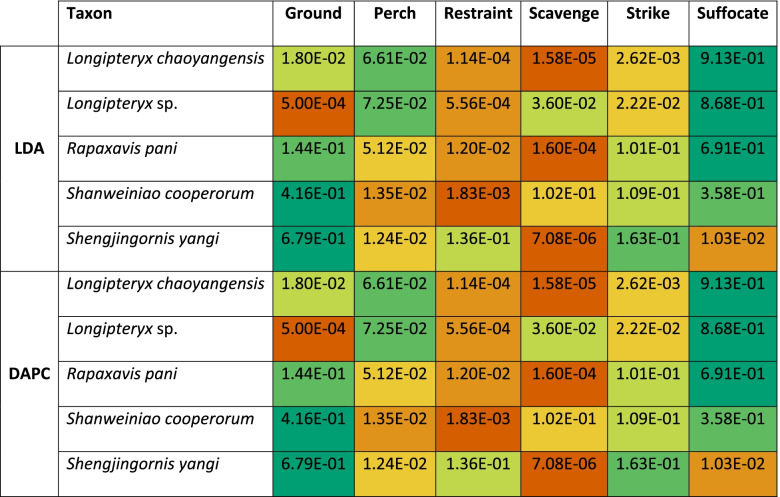
Posterior probabilities predicting longipterygid pedal ecology by LDA and DAPC from TM data from avian unguals. Values with green backgrounds are more likely, values with red backgrounds are less likely. Suffocating raptorial ecology is likely for all longipterygids except *Shengjingornis*. Ground bird ecology is likely for all longipterygids except *Longipteryx* sp. Piercing pedal ecology is not given probability as only one bird, *Pandion haliaetus*, represents that category in our dataset. Note only the large-toothed morphotype of *Longipteryx* had a pes available for measurement

Phylogenetic HSD results comparing extant ecological categories are given in Additional file 1: Table S1. Most pedal ecological categories are indistinct, with the only significant differences being that ground birds are distinct from non-raptorial perching birds and restraining raptors at the *p* = 0.01 level.

Statistically significant phylogenetic signal is present in TM data overall (Table 3) and in each individual input variable (Additional file 1: Table S2). *K*_mult_, a multivariate statistic measuring the phylogenetic signal of an overall phenotype relative to a Brownian motion model [37], is 0.66 (Table 3) for TM data. *K* values for individual TM measurements (Additional file 1: Table S2) range from 0.42 to 0.93.

### Mechanical advantage and functional indices

Univariate comparisons of functional indices (Additional file 1: Fig. S3) show little that is diagnostic between diets. Husking granivores are generally higher in all forms of mechanical advantage (MA) (Additional file 1: Fig. S3A-F) and folivores have uniquely high jaw-opening mechanical advantage (OMA) (Additional file 1: Fig. S3E-F), but otherwise groups broadly overlap.

**Fig. 6:**
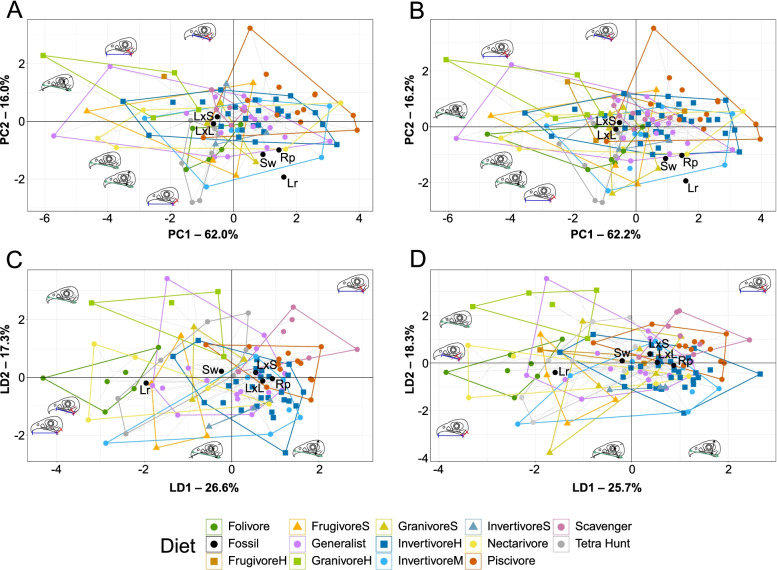
Functional phylomorphospace of avian and longipterygid upper jaws, based on mechanical advantage and functional indices, grouped by diet. Grey lines indicate phylogenetic relationships. Data is visualised with PCA (**A**, **B**) and LDA (**C**, **D**), excluding (**A**, **C**) and including (**B**, **D**) semi-specialists. In PCA (**A**, **B**), PC1 primarily describes AMA, MCH, ACH, and AO and PC2 primarily describes OMA and PMA. In LDA (**C**, **D**), LD1 primarily describes AMA, OMA, and ACH and LD2 primarily describes AMA, PMA, and AO. Diagrams of functional indices from Fig. 2 are included roughly in the orientation they are loaded on the plot. See Additional file 1: Fig. S4 for precise character loadings. Diet abbreviations: FrugivoreH hard frugivore, FrugivoreS soft frugivore, GranivoreS swallowing granivore, GranivoreH husking granivore, InvertivoreH hard invertivore, InvertivoreM medium invertivore, InvertivoreS soft invertivore, Tetra Hunt tetrapod hunter. Taxon abbreviations: Lr *Longirostravis*, LxL large-toothed *Longipteryx*, LxS small-toothed *Longipteryx*, Rp *Rapaxavis*, Sw *Shanweiniao*

PCA plots of MA and functional index data are provided in Fig. 6A, B with character weights plotted in Additional file 1: Fig. S4A-B. Interactive graphs are available in Additional files 4 and 5. PC1 and PC2 explain 78.0% of the total variance. PC1 is driven primarily by relative average cranial height (ACH), anterior jaw-closing mechanical advantage (AMA), relative maximum cranial height (MCH), and relative articular offset (AO) with lesser contributions from OMA and posterior jaw-closing mechanical advantage (PMA) (all in the negative direction). PC2 is driven primarily by PMA in the positive direction and OMA in the negative direction, with lesser contributions from AMA and AO in the positive direction and MCH and ACH in the negative direction. Groups are very poorly resolved in the PCA functional morphospace. Piscivores are mostly constrained to positive PC1 and PC2; husking granivores dominate the negative PC1 and positive PC2 space with hard frugivores also prominent; tetrapod hunters and folivores are mostly constrained to negative PC1 and PC2; and longipterygids other than *Longipteryx* are constrained to the positive PC1 and negative PC2 (both morphotypes of *Longipteryx* plot near the origin). Soft frugivores, generalists, swallowing granivores, invertivores, and nectarivores are all spread across the graph, though all but nectarivores tend to be more concentrated at positive PC1. No diet group occupies a unique region of the functional morphospace. No permutation of PCs 1–5 (representing 98.7% of the variance) isolated any diet in the functional morphospace.

LDA plots of MA and functional index data are provided in Fig. 6C, D with character weights plotted in Additional file 1: Fig. S4C-D. Interactive graphs are available in Additional files 6 and 7. LD1 and LD2 explain 43.9% of the total variance. LD1 is driven by primarily by AMA in the positive direction and OMA and ACH in the negative direction. LD2 is primarily driven by AMA and ACH in the positive direction and MCH in the negative direction. PMA does not have major loading until LD4, and AO is not a major contributor to any LD. Groups are better-resolved than in PCA, but generalists and invertivores still occupy all regions of the functional morphospace. Folivores, nectarivores, and *Longirostravis* are all characterised by negative values of LD1. Soft frugivores inhabit a similar region, but less negative on LD1. Piscivores and scavengers are characterised by positive values of LD1, scavengers tending towards more positive values of LD2 than piscivores. Hard frugivores inhabit a similar region to these two with semi-specialists included, but *Mitu tuberosum* plots at negative LD1. Granivores and tetrapod hunters are characterised by a positive LD2, with granivores trending towards a slightly negative LD1 and tetrapod hunters spreading broadly across LD1. Longipterygids tend to plot in the positive region of LD1 and near zero on LD2, except for *Shanweiniao* which plots near the origin and *Longirostravis* which plots highly negative on LD1. Resolution is poorer when including semi-specialists, in which nectarivores also inhabit all of the functional morphospace and diets separated along LD2 overlap to a greater degree. Discriminant predictions (Table 5) assign longipterygids as either generalists or hard or medium invertivores. None of these assignments are more than 50% confident, and assignment to hard or medium invertivore or generalist reaches a maximum of 90% confidence in the large-toothed *Longipteryx* and *Longirostravis* with semi-specialists excluded, with an average confidence of 87% without semi-specialists and 73% including semi-specialists. DAPC (Additional file 1: Fig. S2C-D) and its predictions (Table 5) are consistent with LDA.

**Table 5:**
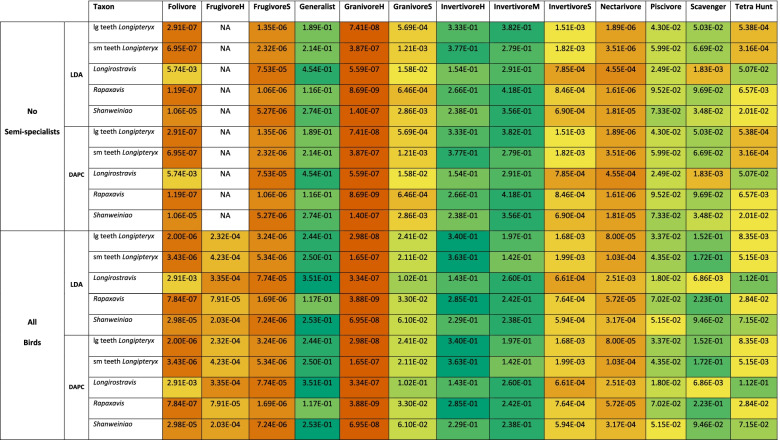
Posterior probabilities predicting longipterygid diet by LDA and DAPC from MA and functional index data from avian upper jaws. Values with green backgrounds are more likely, values with red backgrounds are less likely. Hard/medium invertivory and generalist feeding are likely in all longipterygids, followed by scavenging and piscivory. Hard frugivory is represented only by *Steatornis caripensis* when semi-specialists are excluded, so no predictions are made for FrugivoreH with semi-specialists excluded. lg teeth and sm teeth *Longipteryx* refer to the large-toothed and small-toothed morphotypes of *Longipteryx*, respectively. Diet abbreviations: FrugivoreH hard frugivore, FrugivoreS soft frugivore, GranivoreS swallowing granivore, GranivoreH husking granivore, InvertivoreH hard invertivore, InvertivoreM medium invertivore, InvertivoreS soft invertivore, Tetra Hunt tetrapod hunter

Phylogenetic HSD results comparing MA and functional indices for extant diet categories are given in Additional file 1: Table S3. Skull mechanics of piscivores are significantly different from hard invertivores at the *p* < 0.001 level; from folivores, soft frugivores, generalists, husking granivores, and medium invertivores at the *p* < 0.01 level; and soft invertivores at the *p* < 0.05 level. Nectarivores are significantly different from folivores, soft frugivores, generalists, and husking granivores at the *p* < 0.05 level. Folivores are significantly different from hard invertivores at the *p* < 0.05 level.

Statistically significant phylogenetic signal is present in MA and functional index data overall (Table 3) and in each individual input variable except OMA (Additional file 1: Table S4). *K*_mult_ is 0.87 with semi-specialists excluded and 0.79 with semi-specialists included (Table 3) for MA and functional index data. *K* values for individual MA and functional index measurements (Additional file 1: Table S4) range from 0.49 to 1.34 with semi-specialists excluded and from 0.42 to 1.33 with semi-specialists included.

### Finite element analysis

#### Univariate

**Fig. 7:**
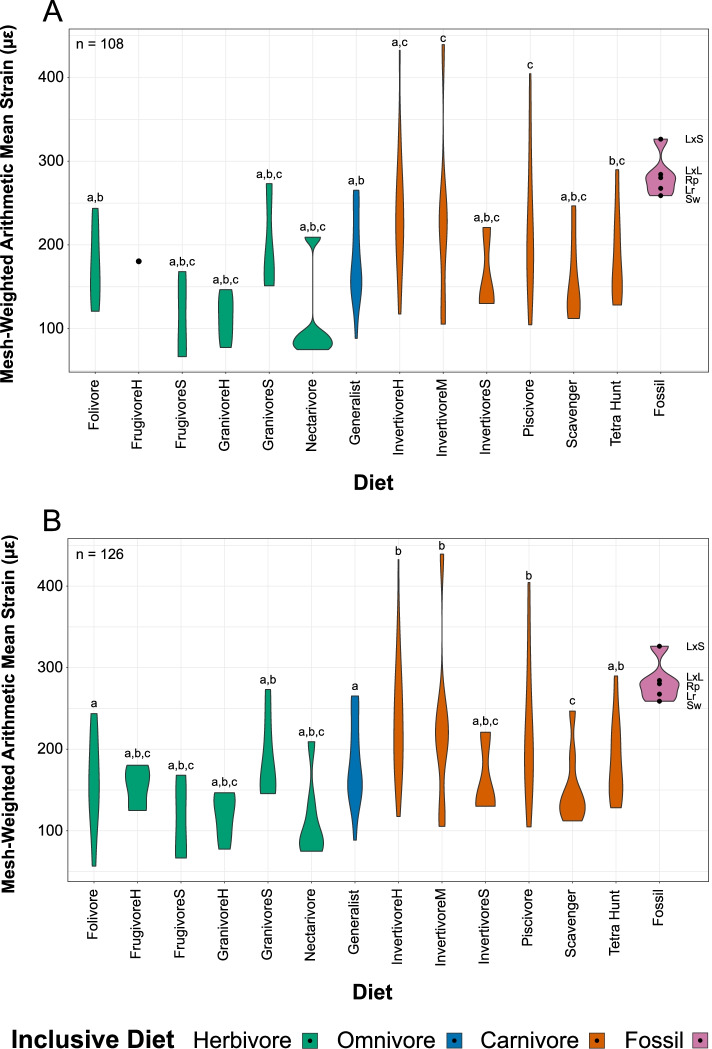
Violin plots of mesh-weighted arithmetic mean (MWAM) strain of bird lower jaw finite element models in this study, organised by more inclusive diets and the whole range of diets considered. Plots are provided excluding (**A**) and including (**B**) semi-specialists. Diets with the same letter above them are not significantly different from one another under phylogenetic HSD of their strain intervals at the *p* = 0.05 level (Additional file 1: Table S5). Diet abbreviations: FrugivoreH hard frugivore, FrugivoreS soft frugivore, GranivoreS swallowing granivore, GranivoreH husking granivore, InvertivoreH hard invertivore, InvertivoreM medium invertivore, InvertivoreS soft invertivore, Tetra Hunt tetrapod hunter. Taxon abbreviations: Lr *Longirostravis*, LxL large-toothed *Longipteryx*, LxS small-toothed *Longipteryx*, Rp *Rapaxavis*, Sw *Shanweiniao*

Mesh-weighted arithmetic mean (MWAM) strain [33] is plotted by diet in Fig. 7. In finite element models that have not been directly validated with experimental strain data, absolute values of performance should be used for comparative purposes only (and then, only among models built from the same assumptions, such as the ones used in this study) [47]. The MWAM values we report here are therefore appropriate for comparing relative performance among our models, but may not be indicative of actual strains in real bone. MWAM strain ranges from 67 to 439 με, with an average of 198 με. The upper limits of MWAM strain in husking granivores are coincident with its lower limit in swallowing granivores. Hard frugivores and soft invertivores have unusually small ranges of MWAM strain, likely due to smaller sample sizes. Nectarivores separate cleanly into psittaciform and non-psittaciform nectarivores, with MWAM strain in other diet groups more evenly spread. Hard and medium invertivores, piscivores, and to a lesser extent tetrapod hunters have representative taxa experiencing higher MWAM strains (με > 270) than any representatives of herbivores, omnivores, scavengers, or soft invertivores. MWAM strain of our models of *Longirostravis*, *Rapaxavis*, and both *Longipteryx* morphotypes fall in this same high-strain region. While some representatives of these carnivorous diets experience higher MWAM strain under loading, most still fall within the range of herbivores and omnivores.

#### Multivariate

For datasets based on the intervals method of interpreting finite element models [34] both including and excluding semi-specialists, PCA, LDA, and DAPC results converge at 75 intervals.

**Fig. 8:**
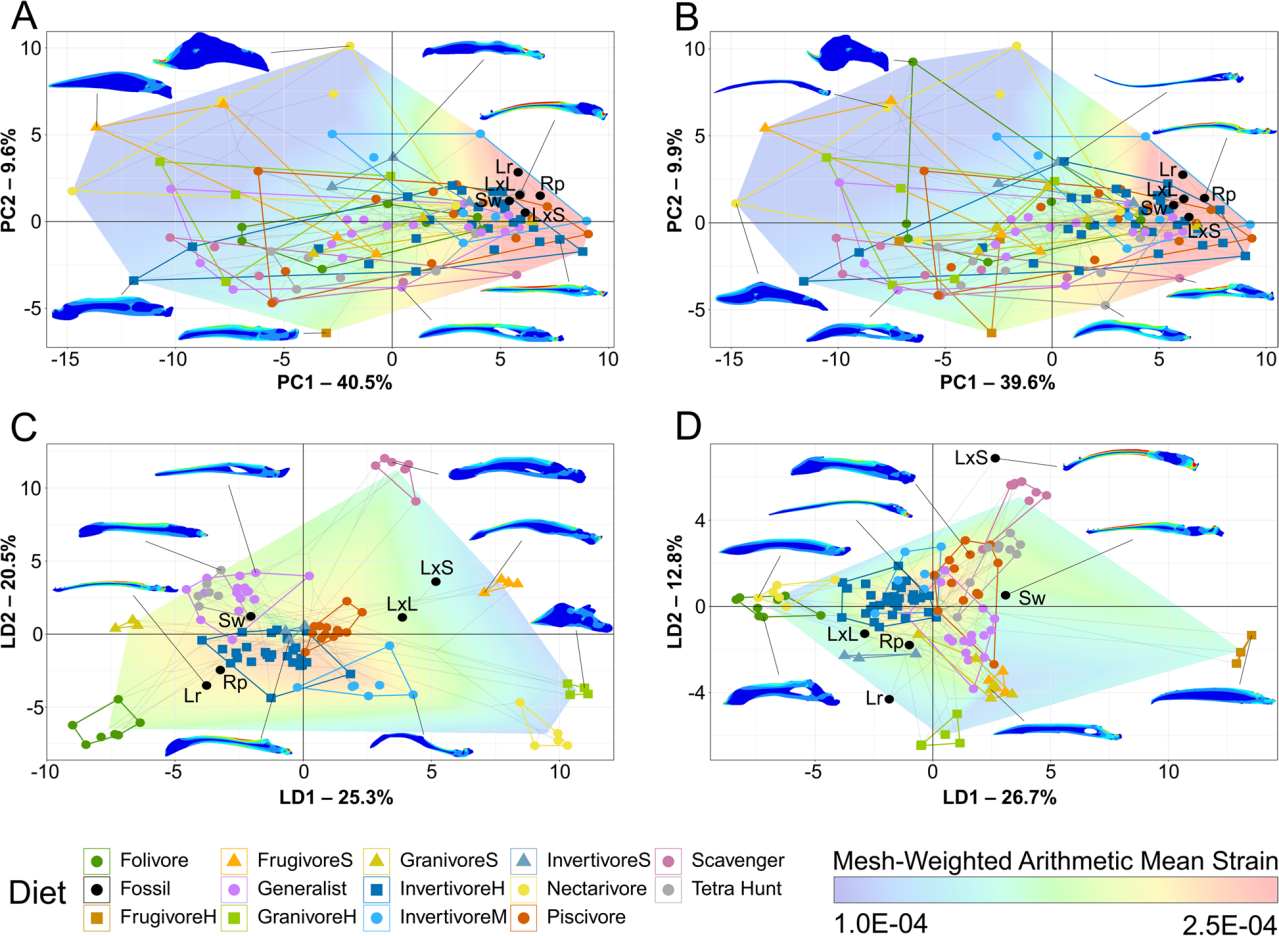
Phylogenetic strain-space of total maximum in-plane principal strain of bird lower jaw finite element models in this study. Grey lines indicate phylogenetic relationships. Contour plots for selected taxa are provided for reference. Results are visualised with PCA (**A**, **B**) and LDA (**C**, **D**), excluding (**A**, **C**) and including (**B**, **D**) semi-specialists. Results are obtained using the intervals method [34] where the percentage of model area under intervals of strain are treated as variables for multivariate analysis. Seventy five intervals were used for PCA and LDA. In PCA (**A**, **B**), overall strain increases along PC1 and unevenness of strain distribution increases along PC2. In LDA (**C**, **D**), LD1 and LD2 have loadings made of various low-strain intervals, with high-strain intervals clustering near the origin. See Additional file 1: Fig. S5 for precise character loadings. Diet abbreviations: FrugivoreH hard frugivore, FrugivoreS soft frugivore, GranivoreS swallowing granivore, GranivoreH husking granivore, InvertivoreH hard invertivore, InvertivoreM medium invertivore, InvertivoreS soft invertivore, Tetra Hunt tetrapod hunter. Taxon abbreviations: Lr *Longirostravis*, LxL large-toothed *Longipteryx*, LxS small-toothed *Longipteryx*, Rp *Rapaxavis*, Sw *Shanweiniao*

PCA plots of FEA intervals data (“strain-space”) are provided in Fig. 8A, B with character weights plotted in Additional file 1: Fig. S5A-B. Interactive graphs are available in Additional files 8 and 9. PC1 and PC2 (Fig. 8A, B) explain 50.1% of the total variance. Plot weightings (Additional file 1: Fig. S5), maps of MWAM strain (Fig. 8), and manual checks of the contour plot trends (Additional files 10 and 11) show that negative PC1 represents areas of low strain (high strength) and positive PC1 areas of high strain (low strength). Contour plots show that in positive PC2 strain tends to be more concentrated (models have areas of very high and very low strain), and in negative PC2 strain tends to be more evenly distributed (model strain is more equal throughout). We investigated PCs 3-24 (along with 1 and 2 representing 91% of explained variance) and found them to have less useful weightings than PC 1 or 2, though including PC3 does better separate some diet groups (Additional files 8 and 9). Dietary groups are poorly resolved in the PCA strain-space. Invertivores and piscivores tend to plot at positive PC1 while herbivores and scavengers tend to plot at negative PC1, nectarivores tend to plot at positive PC2 while tetrapod hunters tend to plot at negative PC2. However, nearly all these groups have members which plot far from their main cluster. The most negative PC1 space (representing the strongest jaws) is inhabited by an undiagnostic mix of carnivores, herbivores, and omnivores. The most positive PC1 space (representing the weakest jaws) is primarily inhabited by carnivorous and omnivorous taxa. All longipterygid jaws plot at positive PC1 and PC2 (weakest jaw area, with slightly more concentrated strain) of the strain-space.

LDA plots of FEA intervals data are provided in Fig. 8C, D with character weights plotted in Additional file 1: Fig. S5C-D. Interactive graphs are available in Additional files 12 and 13. LD1 and LD2 (Fig. 8C, D) explain 45.8% of the variance. LD weightings are disordered: intervals of strain follow no clear pattern along the axes and intervals of nearly equal strain may have completely opposite weightings (Additional file 1: Fig. S5C-D). Instead, jaws with areas under high amounts of strain (weaker jaws; MWAM strain ≈ 230 με) tend to plot near the origin while jaws with large areas under low amounts of strain (stronger jaws; MWAM strain ≈ 110 με) plot farther from the origin (Fig. 8C, D, Additional file 1: Fig. S5C-D, Additional files 14 and 15). We plotted all LDs and this trend persists across them. Most diet groups are very distinct in the plot. The quasi-random spread of the LD loadings (Additional file 1: Fig. S5C-D), though, means that groups far from the origin are less distinct from one another than they appear. Thus, it is safe to say that herbivores and scavengers are distinct from generalists, invertivores, piscivores, and tetrapod hunters. With semi-specialists included resolution decreases with soft frugivores, swallowing granivores, and scavengers partially overlapping with the near-origin clusters and all herbivorous groups showing less separation (though soft invertivores do become more distinct from near-origin groups within the first two LDs). Longipterygid jaws tend to plot near the near-origin clusters except for *Longipteryx* for which both morphotypes plot in an intermediate space with semi-specialists excluded and the small-toothed morphotype plots near herbivores with semi-specialists included. Discriminant predictions with and without semi-specialists (Table 6) find hard invertivory and piscivory most likely for longipterygids followed by tetrapod hunting and generalist feeding. Both morphotypes of *Longipteryx* have lesser affinities for medium invertivory and scavenging and *Longirostravis* and *Rapaxavis* have lesser affinities for swallowing granivory (significant affinities with semi-specialists excluded). The large-toothed morphotype of *Longipteryx* shows an affinity for nectarivory, *Longirostravis* and *Rapaxavis* show an affinity for folivory, and *Shanweiniao* for scavenging only with semi-specialists included. DAPC (Additional file 1: Fig. S2D-E) and its predictions (Table 6) are consistent with LDA.

**Table 6:**
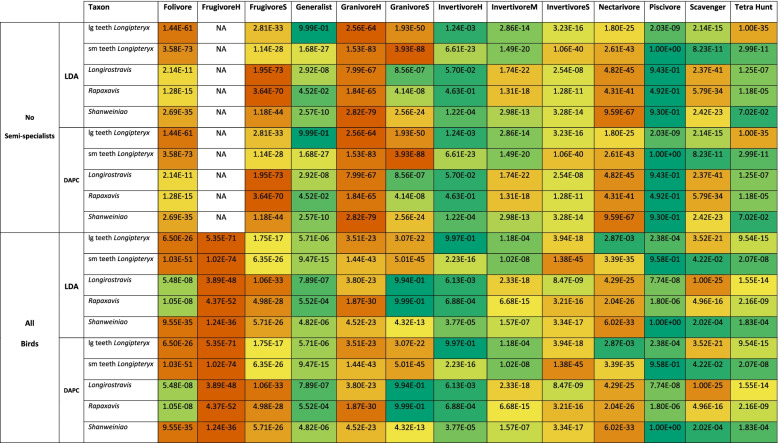
Posterior probabilities predicting longipterygid diet by LDA and DAPC from FEA data from avian lower jaws using the intervals method [34]. Values with green backgrounds are more likely, values with red backgrounds are less likely. Hard invertivory and piscivory are most likely in all longipterygids, followed by tetrapod hunting, generalist feeding, and medium invertivory. Swallowing granivory is consistently likely in *Longirostravis* and *Rapaxavis*. Some predictions are highly confident but inconsistent, e.g. nectarivory in large-toothed *Longipteryx*, which we treat as inconclusive. Hard frugivory is represented only by *Steatornis caripensis* when semi-specialists are excluded, so no predictions are made for FrugivoreH with semi-specialists excluded. lg teeth and sm teeth *Longipteryx* refer to the large-toothed and small-toothed morphotypes of *Longipteryx*, respectively. Diet abbreviations: FrugivoreH hard frugivore, FrugivoreS soft frugivore, GranivoreS swallowing granivore, GranivoreH husking granivore, InvertivoreH hard invertivore, InvertivoreM medium invertivore, InvertivoreS soft invertivore, Tetra Hunt tetrapod hunter

Phylogenetic HSD results comparing strain intervals of extant diet categories are given in Additional file 1: Table S5. Folivores are significantly different from medium invertivores, piscivores, and tetrapod hunters at the *p* < 0.05 level. Generalists are significantly different from hard invertivores at the *p* < 0.01 level and from medium invertivores and piscivores at the *p* < 0.05 level. These differences are noted above the violin plots in Fig. 7.

No statistically significant phylogenetic signal was detected in intervals data with or without semi-specialists included (Table 3). With semi-specialists excluded, *K*_mult_ is 0.41 for the PCA dataset and 0.43 for the LDA dataset (Table 3). With semi-specialists included, *K*_mult_ is 0.33 for the PCA dataset and 0.32 for the LDA dataset (Table 3).

## Discussion

### Body mass

Body masses of all longipterygid species were estimated in past publications [1, 21] using multivariate regression of avian skeletal measurements. For convenience, these are supplied in Table 7 alongside body mass estimations of two undescribed specimens of *Longipteryx* sp. (STM 7-156 and STM 8-117).

**Table 7:** Body masses of longipterygids, based on the regression equations of [21]. Masses are compiled from [1, 21] and converted into grams for easy comparison to the cut-off points in Fig. 4. STM 7-156 and STM 8-117 are newly measured in this study. DNHM D2889 and STM 8-117 represent the large-toothed morphotype of *Longipteryx*, STM 7-156 represents the small-toothed morphotype, IVPP V12325 appears to represent the small-toothed morphotype but the rostral region is too poorly preserved to be sure

Taxon	Specimen	Mean mass estimate (g)	Lower mass estimate (g)	Upper mass estimate (g)
*Longipteryx chaoyangensis*	DNHM D2889	154	124	184
*Longipteryx chaoyangensis*	IVPP V12325	193	155	230
*Longipteryx* sp.	STM 7-156	44	36	53
*Longipteryx* sp.	STM 8-117	206	166	246
*Longirostravis hani*	IVPP V11309	39	32	47
*Rapaxavis pani*	DNHM D2522	47	38	56
*Shanweiniao cooperorum*	DNHM D1878/1, DNHM D1878/2	57	46	68
*Shengjingornis yangi*	PMOL AB00179	340	274	406

Within carnivores and herbivores, body mass appears to be generally conserved phylogenetically. In both groups, excluding and including semi-specialists, *K* statistics are above 1 (Table 3). This means that the masses of species are more similar to their close relatives than expected from random evolution. For mass of birds across all diets, however, K is just below 1 with semi-specialists excluded. There is no significant phylogenetic signal in body mass data with semi-specialists included.

In a previous review [1], we hypothesised that body mass could distinguish between vertivores and invertivores (Fig. 3C, D), with vertivores typically larger than invertivores, and our findings support this hypothesis. Phylogenetic HSD finds masses of vertivores and invertivores to be significantly different (*p* = 1.00E−3). We determine a mass cut-off point between vertivores and invertivores of either 324 g or 439 g depending on the dataset. To be conservative, this discussion will treat masses of 300 to 450 g as indeterminate for distinguishing between carnivores, with invertivores predicted below 300 g and vertivores above 450 g. Our more precise breakdown of diets (Fig. 3B) provides preliminary support for our previous hypothesis [1] that separation of predators by body mass occurs based on prey body mass. Tetrapod-hunting birds have a relatively narrow peak body mass (standard deviation = 2.0 g) while fish-hunting (standard deviation = 4.3 g) and invertebrate-hunting birds (standard deviation = 4.9 g) have a wider, flatter distribution. Accordingly, mass distributions of tetrapods tend to have narrower peaks than those of non-tetrapod fish [48]. While large-scale data for invertebrate mass distribution is not available, local distributions of invertebrate mass [49] and global distributions of insect length [50] seem to imply a broad peak of invertebrate masses as well. Together these imply that, at least on macroecological scales, predatory bird masses increase with prey body mass. However, future studies with more precise records of bird prey items and their masses are needed to fully validate this hypothesis. Soft invertivores, mostly filter feeders in this study (*sensu* [51], see “Diet assignment” in “Methods”), oppose this trend by being, on average, as massive as vertivores. Our sample size of filter feeding birds is small (*n* = 2), so further work is needed to determine if this persists in larger samples. We expect it will, as other aquatic filter feeders including baleen whales, whale sharks, and pachycormiform fish all convergently evolved gigantism [52], presumably to increase the volume of water/sediment sieved in each pumping cycle. Scavengers, the carnivores with the largest average body masses in this study, have no need to kill prey and no obligation to lift it. Thus, we would not expect their mass to scale to prey mass. Instead, they are believed to experience selective pressures towards larger body size to avoid kleptoparasitism [53], have larger fat stores, and search for carrion more efficiently [54].

When plotting body mass by diet (Fig. 3A, B), we noticed an apparent separation by diet in herbivores as well. While less significantly so than in carnivores (phylogenetic HSD *p* = 3.60E−02 for herbivores, *p* = 1.00E−3 for carnivores), folivores and frugivores tend to be more massive than granivores and nectarivores (Fig. 4A, B). Two cut-off points are calculated as optimal: 249 g and 408 g. As above, for this discussion, herbivores below 250 g will be assumed as granivores or nectarivores, those between 250 g and 400 g as indeterminate, and those above 400 g as folivores or frugivores. There is heavy phylogenetic influence on this split, however, signified by phylogenetic HSD and Tukey’s HSD returning very different *p*-values when comparing the two (Table 2). Food size can explain some of the mass trends once again: seeds are housed inside fruits, so fruits of a given plant species are necessarily larger than their seeds. This is exacerbated by semantic biases, where small fruits are often called seeds. For instance, grains, from whence “granivore” takes its name, are botanically fruits [55]. In other words, the mass distinction between frugivores and granivores seems to be based on the size of food taken, but differences between the botanical definition of seeds and fruits and their operational definitions in diet studies could artificially reinforce this distinction. Nectarivore and folivore masses have more established explanations. Nectarivore mass has long been known to be constrained by the small amounts of nutrient-poor nectar in flowers [56] and increasing specialisation for nectar consumption accompanies a reduction in body mass [57, 58]. Folivory is believed to necessarily increase gut mass and gut retention time, increasing body mass to the point that may impede flight [59]. Indeed, most folivorous taxa in our study are terrestrial (Anhimidae, Phasianidae) or aquatic (Anatidae, Rallidae). Of the two arboreal folivorous taxa studied, *Opisthocomus* (the hoatzin) is a poor flier [60] and *Micropsitta* (a pygmy parrot) is a lichen-eating specialist [61] whose assignment as a folivore is debatable.

Predicted body masses for longipterygids range from 32 g as a lower estimate for *Longirostravis* [21] and 406 g as an upper estimate for *Shengjingornis* [1] (Table 7). *Shengjingornis* is unusually large for the clade, with the next largest taxa, *Longipteryx* and *Shanweiniao*, having upper mass estimates of 246 and 68 g [21] (Table 7), respectively. *Shengjingornis* falls within the indeterminate range (i.e. between the two cut-off points calculated) for both carnivore and herbivore masses, so there is no evidence of its diet from mass. Masses for all other longipterygids fall below cut-off point values for carnivores and herbivores, so no longipterygid other than *Shengjingornis* is likely to be a vertivore, folivore, or frugivore.

### Traditional morphometrics

We find pedal TM generally effective at distinguishing raptorial birds, ground birds, and perching birds. This is congruent with several previous studies [26, 62–64]. Ground birds tend to have very straight claws with relatively large DII, raptorial birds tend to have very curved claws with relatively large DI and DII, and perching birds tend to have very curved claws with relatively small DI and DII. However, the heavy overlap of some groups in PCA (e.g. scavenger and perch, strike, and restrain; see Fig. 5A) and distinct clusters within some groups (see “Results”) highlights the subjectivity of our ecological categories and indicates that some may need to be split or merged. If so, this can introduce artificial trends into LDA and DAPC, so we will primarily refer to PCA results when interpreting these data.

Unlike past studies [1, 24, 27], we find raptorial pedal morphologies do not separate cleanly in the morphospace. Striking and restraining raptors other than shrikes overlap almost completely in PCA (Fig. 5A), restraint is the category which striking overlaps with most heavily in LDA (Fig. 5B), and the two are not significantly different in phylogenetic HSD (Additional file 1: Table S1). Suffocation specialists partially overlap with these two groups in PCA, though three of the four taxa that overlap (*Bubo virginianus*, *Pulsatrix perspicillata*, and *Ninox novaeseelandiae*) are known to occasionally hunt large mammals [65, 66] or, in the case of *N*. *novaeseelandiae*, mammals much larger than its typical insect prey [67]. Thus, it is possible that this region of the morphospace, representing curved claws and enlarged DI and DII, more generally represents adaptations for taking large prey (*sensu* [24]) rather than any specific raptorial style. The region inhabited by the remaining owls, with claws roughly equal in size and less recurved than most perching birds, might therefore be specialised for hunting only small prey. Shrikes, however, have straight claws and reduced DI and DII, an even more extreme divergence from birds taking large prey despite shrikes taking prey nearly as large as they are [68, 69]. The “reduction” in DI and DII appears to be artificial from choosing DIII as the reference digit, as DIII is enlarged in shrikes (DIII/DIV ALo average 1.73 in shrikes, DII/DIV ALo average 1.61 in other restraint predators) and DIII likely performs the pinning role DII has previously been reported playing in raptors [24]. When size ratios are made to digit IV (Additional file 1: Fig. S6), shrikes cluster with other restraint raptors. Why shrike claws are so straight is less clear. One explanation would be a difference in handling time, as prey handling time for the loggerhead shrike [68] is on average 300 times shorter than in traditional raptors [70]. Another possibility is that a flatter foot may increase stability during the distinct vertebrate-prey-shaking behaviour true shrikes use on large prey like mice [71]. This behaviour is not reported in the similarly sized helmetshrike *Prionops plumatus* [72] whose claws plot alongside hawks and eagles.

Previous studies which find resolution between types of raptorial predation in pedal morphometrics [1, 24, 27] incorporate toe lengths as well as claw measurements. Thus, their exclusion here may be the reason for our lack of resolution. Einoder and Richardson [27] found talon measurements to describe raptorial specialisation better than any other hindlimb measurements including those of toes (compare their table 2 and fig. 2, 3, 5, and 8), undermining this possibility. Toe length does play a prominent role in the analyses of [1, 24], however. In particular, claw size ratios to toe lengths play a prominent role in separating restraining raptors from striking raptors along CA2/PC2 and suffocating raptors from all other birds along CA1/PC1 (compare fig. S1 in [1] to Additional file 1: Fig. S1; CA refers to the axes in Correspondence Analysis used by [1, 24, 73]). Future work may seek to focus on skeletal specimens in which toes are articulated due to incomplete maceration. All previous studies which successfully discriminate raptorial subtypes use data from fresh carcasses or skins rather than skeletal specimens, focus heavily on raptorial birds, and have sample sizes just over half that of the current study. It therefore remains unclear if the lack of resolution between raptors is due to the exclusion of toe measurements, use of skeletal material, or simply from more complete sampling capturing greater morphological diversity. It is noteworthy that the study of avian pedal morphometrics with the largest sample size [25] also found some of the greatest overlap between the pedal ecological groups studied.

Hedrick et al. ([36] pg. 11553) have recently proposed that morphospace grouping of raptorial bird claws reflects phylogeny rather than function. Significant phylogenetic signal is present in the TM data overall (Table 3) and each individual variable (Additional file 1: Table S2), and phylogenetic HSD finds most groups not significantly different (Additional file 1: Table S1). Taxonomic affiliation also explains some clusters in PCA (see “Results”). Most notably, the ground birds are split into megapodes and passerines (specifically larks) with very straight claws and cranes and tinamous with weakly recurved claws. We suspect this relates to habitat differences, as cranes and tinamous tend to inhabit wet floodplains or swamps while larks and megapodes are more known to inhabit dry scrub areas. The large separation of cranes along PC2 may reflect sexual dimorphism, with a female *Grus canadensis* having a much smaller and less recurved DII than a male *Balearica pavonina*. Alonso et al. [74] found male *Grus grus* to have a longer DIII than females, proposing the difference as related to elevated rates of combat in males. We suspect this extends to the highly recurved DII in cranes as well, but have been unable to confirm this in the literature. Our results also show most phylogenetic groups of perching birds being distinct from one another, but all are enveloped within the parrot morphospace. Despite this, however, we believe phylogeny does a worse job of explaining the data than pedal ecology (Additional file 1: Fig. S7). Passerine birds cover nearly the entire morphospace, and restraint predatory passerines have far more recurved claws and more interdigital size variation than ground bird passerines. Despite some being distinct, all subgroups of perching birds inhabit a region of the morphospace shared only with scavengers (who, in turn, mainly use their feet for perching). Most telling, though, is the clustering of macropredatory raptors (Accipitriformes, Strigiformes, Falconiformes), the seriema *Cariama cristata* (Cariamiformes), and the helmetshrike *Prionops plumatus* (Passeriformes) (Additional file 1: Fig. S7) representing five orders across Telluraves [75]. Also, while phylogenetic signal is present in all of the TM data, *K*_mult_ (= 0.66) and all *K* values (0.42–0.93, $\overline{\mathrm{x}}$ = 0.69) are less than 1, meaning claw morphometrics are less similar than expected under a random evolution model. This in turn implies the pes is evolving under adaptive evolutionary pressures [45]. Given these polyphyletic clusters and homoplastic signal, we suggest that our pedal morphometric data reflects pedal ecology despite the effects of phylogeny.

Longipterygids generally have intermediate levels of claw curvature and little interdigital size variation. The major exception to this is *Shanweiniao*, whose very low claw curvature makes it by far the most similar to extant ground birds. *Shengjingornis* seems at first glance to have similar proportions to extant pinning and striking raptors, though these seem driven more by a noticeably reduced DIII than an enlargement of DI and DII [13]. As *Shengjingornis* plots in regions of heavy overlap in all graphs, we consider pedal morphometrics to be inconclusive in this taxon. *Rapaxavis* and both *Longipteryx* specimens plot in a central region of the morphospace due to their relatively equal claw sizes and intermediate curvatures. While this region is undiagnostic when DIII is used as a reference (Fig. 5), PCA with DIV used as the reference digit (Additional file 1: Fig. S6) and LDA predictions (Table 4) find these taxa most similar to suffocating raptors (in this study exclusively owls)*. Longipteryx* has higher claw curvature than any other longipterygid genus (average 91° in *Longipteryx*, 78° in other longipterygids), driving its affinity towards non-raptorial perching birds (Table 4), with *Rapaxavis*, *Shanweiniao*, and *Shengjingornis* having more affinity with ground birds. The results for *Rapaxavis* run counter to the findings of Morschhauser et al. [17] who found *Rapaxavis* to be arboreal based on non-ungual phalangeal proportions, though as owls are mainly arboreal this possibility cannot be ruled out. We thus find *Shanweiniao* most likely to be a ground-dwelling bird and *Rapaxavis* and *Longipteryx* likely to display raptorial behaviour with small animals (*sensu* [24]). Results for *Shengjingornis* remain ambiguous due to its pedal proportions overlapping with most pedal ecological groups.

It is possible that the ratio of curvature between the ungual and keratin sheath is radically different in avians than non-avian avialans, meaning unguals may not accurately represent the whole claw used by longipterygids. Impressions resembling keratin sheaths are present alongside the right DI and DII unguals of *Longipteryx* DNHM D2889 [16], and the ratio of the sheath angle to the ungual angle (respectively 1.3 and 1.9) is akin to that previously reported for DI and DII in eagles ($\overline{\mathrm{x}}$ = 1.4 (table S1 in 24)) and DIII across birds ($\overline{\mathrm{x}}$ = 1.2 (table S1 in [26])). *Rapaxavis*, in contrast, preserves what appears to be a disarticulated keratin sheath [17, 76] less curved than the unguals. While it cannot be accurately measured due to loss of the base, a reasonable range of sheath to ungual curvature (~0.6–0.7) falls below that reported in any extant bird (0.9 in DIII of tinamou *Rhynchotus rufescens* (table S1 in [26])). Future work using techniques designed to identify soft tissue remains, e.g. laser-stimulated fluorescence [77], could help to evaluate the authenticity of these sheath impressions. While only small datasets for lepidosaurs have been examined, it does generally seem that birds and lepidosaurs have similar differences between ungual and claw sheath curvature (fig. 4 in [26]), meaning unguals should be reliable predictors of claw shape for a large extant phylogenetic bracket around Longipterygidae. For this reason, in lieu of thorough examinations of non-avian avialan claw sheath impressions, we consider our TM results ecologically informative of Longipterygidae.

### Mechanical advantage and functional indices

We find mechanical advantage and the additional functional indices which seemed most promising in our previous review [1] to be very poor at predicting diet across birds. All three forms of measured mechanical advantage have low *K* values (Additional file 1: Table S4) which would predict that these have some adaptive significance [45, 78]. ACH, in contrast, has a high *K* value (Additional file 1: Table S4) which would imply some evolutionary constraint [78]. MA being undiagnostic is in line with the findings of Navalón et al. [18]. However, while some groups (generalists, invertivores, often frugivores and nectarivores) span the whole functional morphospace and thus can never be ruled out, several diet categories do display consistent mechanical traits that, if absent in fossil taxa, can be used to rule them out as possible diets.

Husking granivores, as noted previously [18, 29], have especially high jaw-closing mechanical advantage (AMA and PMA; Fig. 6, Additional file 1: Fig. S3A-D). This allows them to exert greater forces on seeds, which can allow for faster seed husking and more efficient feeding [42]. We do not, however, find AMA or PMA to be high in herbivores and low in invertivores, as previously reported [29, 79]. This is likely due to the large phylogenetic breadth of our sample. Miller and Pittman [1], reanalysing the work of Corbin et al. [29], found a moderate correlation between AMA and plant matter consumption in passerine birds, but nearly no correlation when a single columbiform data point was added. No bird diet groups have diagnostically low jaw-closing mechanical advantage.

Folivores are characterised by a high jaw-opening mechanical advantage (OMA; Fig. 6, Additional file 1: S3E-F). Why folivores require jaws that open slowly but powerfully, however, is unclear. Piscivores have relatively low OMA combined with a low relative articular offset (OA; Fig. 6, Additional file 1: S3E-H), to the point that they are the most commonly significantly different diet in phylogenetic HSD (Additional file 1: Table S3). We suspect both are adaptations to maintaining grip on slippery, muscular fish. A low relative articular offset causes the upper and lower jaws to meet in a scissor-like fashion, pushing fish in them forwards. Many piscivorous birds (e.g. *Gavia stellate*, *Mergus serrator*, *Morus bassanus*) have a rostral hook to the bill, which this scissor-like occlusion pushes the fish towards. This causes the bill to essentially wrap around the slippery prey, increasing contact area. Should the fish slip out of position, a low OMA allows the bird to quickly open its jaw and adjust position to maintain the grip. This may also aid in rapid swallowing, as kleptoparasitism is particularly common among seabirds [80] which are usually piscivorous.

Swallowing granivores, tetrapod hunters, scavengers, and soft frugivores separate in the functional morphospace to a lesser degree (scavengers only in LDA), and the driving forces of their separation are more complex than the diets above. Swallowing granivores and tetrapod hunters inhabit similar regions across multivariate analysis (Fig. 6, Additional files 4, 5, 6 and 7), driven mainly by a mixture of a below average AMA and PMA, relatively high OMA (but below that of folivores), and arguably a high MCH (neither group has particularly tall skulls, but they lack representatives with short skulls; Additional file 1: Fig. S3). One possible explanation is that the similarities stem from requirements of sensation rather than feeding (with all four indices able to be caused by an expansion of the cranial region), as locating both animal prey and ripened seeds require keen eyesight and advanced visual processing. Scavengers do not display strikingly high or low values of any functional index, and thus plot in a relatively tight cluster near the origin in PCA (Fig. 6A, B). They do, however, have less range in functional indices than other groups (Additional file 1: Fig. S3) which we suspect reflects tight constraints on what, mechanically, makes a successful scavenging bird. As Hertel [81] points out, scavenging birds often have little control over their food sources and abundant competition, so they need to feed particularly efficiently in order to survive.

Longipterygids’ MA and functional indices give no clear diagnosis of their diet, as one may expect from the poor predictive power in avians. Hard/medium invertivory and generalist feeding are recovered as the most likely diets of longipterygids (Table 5), but these are also the diets which spread farthest across the functional morphospace and so likely represent a “default” prediction for this analysis. Scavenging and piscivory are also recovered as somewhat likely but their relative likelihood is sensitive to the position of the quadrate. When the quadrate is placed to the extreme posterior, scavenging becomes the most likely longipterygid diet and piscivory enters the top three (Additional file 1: Table S6). Some of the particularly distinct diets mentioned above, however, can be ruled out. Longipterygids plot far from husking granivores in PCA (Fig. 6A, B) and GranivoreH is assigned the lowest probability for longipterygids by discriminant equations (Table 5). Most longipterygids also plot far from folivores and soft frugivores, though the unusually high OMA of *Longirostravis* (0.24, $\overline{\mathrm{x}}$ = 0.17 for other longipterygids; Additional file 1: Fig. S3E-F) gives it an unusually high affinity with these groups. This involves some uncertainty, however, discussed in the following paragraph. In short, functional indices of the skull indicate that longipterygids were unlikely to be husking granivores, soft frugivores, or folivores (except possibly *Longirostravis*, see below).

The unusually high OMA of *Longirostravis* may be a by-product of the limits of our reconstruction (see “Methods”). The cranium of the only described specimen of *Longirostravis* (IVPP V11309) is crushed and indistinct with a prominent crack running through it ([82] pg. 86), meaning its caudal extent is obscure. Our initial reconstruction of *Longirostravis* which used the full extent of the skull had an unusually long cranium, and even after a roughly 30% reduction in its length to create the version in Fig. 1C, it is still more elongate than other longipterygids. It may be that the cranial bones of IVPP V11309 were sheared caudally or spread apart by cracks in the slab to a greater extent than we believe, and we thus overestimate its OMA. It is of note that in the results of sensitivity analysis of our reconstructions (Additional file 1: Table S6) folivory assignment is highly sensitive to the placement of the quadrate (with a rostral shift of the quadrate increasing OMA and folivore likelihood), and in fact folivory becomes the most likely assignment for *Longirostravis* at our anterior extreme for the error of the quadrate placement. If this is not an artefact of reconstruction, the increased OMA in *Longirostravis* may represent a unique feeding adaptation. *Longirostravis* has a uniquely narrow snout tip among longipterygids, which has led previous studies to propose it as a probing feeder [15, 17]. However, unlike modern probing feeders who insert and remove their beaks from substrate while remaining nearly closed [83], we propose the higher jaw-opening mechanical advantage in *Longirostravis* (if truly present) could have allowed it to open its jaws after inserting them into substrate, increasing its tactile range and allowing it to more efficiently remove larger prey items from the substrate. If true, one would also expect a large retroarticular process on the lower jaw for increased attachment area of jaw-opening muscles [84], though the holotype does not preserve this part of the jaw [15] (Fig. 1C).

### Finite element analysis

We find FEA to be overall ineffective at isolating specific diets, but very effective at separating swathes of diets. The large dietary overlap in MWAM strain (Fig. 7) and the first three principal components of intervals data (Fig. 8A, B, Additional files 8 and 9) suggests the overall structure of the data is not driven by diet. A reasonable null hypothesis would be that the data is driven by phylogeny, but *K*_mult_ tests of intervals data find no significant phylogenetic signal (Table 3). The partial separation of herbivores and carnivores in strain-space (Fig. 8A, B, Additional files 8 and 9), high separation when plotting LDA (Fig. 8C, D), and high confidence of LDA predictions (Table 6) all indicate that diet information can be reliably extracted from FEA results. LDA in particular is effective at splitting birds into two dietary groups. However these groups are not, as expected [1], those feeding on hard foods (in which initiating a crack via puncture is difficult) and soft foods (in which initiating a crack via puncture is easy). Nor does LDA discriminate between birds consuming hard foods or tough foods (in which propagating a crack is difficult, regardless of the mode or ease with which the initial crack forms). Instead, generalists and birds specialising in foods that are neither hard nor tough tend to have weak jaws (high strain when loaded). Specialists feeding on hard foods, tough foods, or foods both hard and tough tend to have stronger jaws (low strain when loaded).

Husking granivores and scavengers have the most consistently strong jaws. Both tend to have low MWAM strains ($\overline{\mathrm{x}}$ = 110 and 160 με respectively; Fig. 7), partially separate out in PCA (Fig. 8; scavenger separation clearer in Additional files 8 and 9), and plot in low-strain regions in LDA (Fig. 8C, D). This is unsurprising for husking granivores, which need to break through seed coats when feeding and have been found to have high jaw strength in past studies [85, 86]. High jaw strength in scavenging birds has less clear reasoning. While mammals which heavily scavenge have been recorded as having high bite forces [87], this is associated with bone-crushing, which avian scavengers do not perform (aside from the bearded vulture *Gypaetus barbatus* [88] which had the weakest jaw of non-semi-specialist scavengers and typically uses tools to aid in crushing). There may be phylogenetic bias at play, as most scavenging birds are members of Accipitrimorphae (*sensu* [89]), but *Phalcoboenus australis* (Falconidae) and *Leucophaeus scoresbii* (Laridae) both plot alongside them. The vultures themselves are also ecologically diverse, with representatives of rippers, gulpers, and scrappers in the sample [90, 91]. We hypothesise, then, that scavenging birds need strong jaws to remove the tough, potentially desiccated, and hardened flesh of carcasses regardless of ecological specialty. This would explain why the semi-specialist scavenger *Chionis minor* has a weaker jaw than other avian scavengers, as it chiefly steals freshly caught food from other birds [92]. Hard frugivores arguably belong here as well, as they follow similar trends when *Mitu tuberosum* (which only a juvenile specimen was available to represent) is removed. This would not be surprising, as mechanically their feeding is similar to husking granivores.

Folivores, soft frugivores, swallowing granivores, and soft invertivores have the next most distinctly strong jaws. While they do not separate cleanly in PCA, they have generally low MWAM strains ($\overline{\mathrm{x}}$ = 170, 110, 200, and 160 με respectively; Fig. 7) and plot in low-strain regions in LDA (Fig. 8C, D; note soft invertivores separate more when semi-specialists are included and plot very far from the origin along LD9 with them excluded [LD9 has weightings similar to the weightings which separate soft invertivores in the LDA plot with semi-specialists included]). As explained in the “Methods” section, two of the three soft invertivores included in this study are filter feeders. While trends are only tenuous given a small sample size, it would make sense for filter feeders to have jaws adapted for the unique demands of forcefully sieving water through their mouths [51, 93]. Alternatively, as discussed in the “Body mass” section, filter feeders face selection for larger body size and particularly larger head size to increase the volume of material filtered. It may be particularly easy to do this by increasing the depth of the jaw, which strengthens the jaw during biting as a side effect by increasing the second moment of area. Folivores and soft frugivores are both feeding on food that is tough but not hard [94]. So, while high jaw strength is not required to process their food directly, their food is tough enough that greater strength is needed to acquire and/or disassemble it. This aspect of disassembly likely explains the higher strength adaptations in swallowing granivores as well, as while they are not crushing hard food in their jaws, they are often still detaching them from plants housing the seeds. Thus, it appears that, in avian jaws, FEA has trouble distinguishing between toughness and hardness of foods in the diet.

Unexpectedly, the final diet group with “strong” jaws is nectarivorous birds. Nectarivorous birds rarely bite down on anything, feeding on nectar by rapidly inserting and removing their tongues from flowers [95]. Thus, one would expect their jaws to experience high strains when loaded as if biting, but the opposite is true. Nectarivores in this study have some of the lowest MWAM strains ($\overline{\mathrm{x}}$ = 110 με; Fig. 7) and consistently plot in multivariate regions of low strain. Some of this can be explained by phylogenetic inertia. Parrots (Psittaciformes) are known to have strong jaws and strong evolutionary control on skull shape [96], and make up roughly half of the nectarivores studied. The other half, hummingbirds (Trochilidae), are more surprising to recover having jaws with low strain when loaded. We attribute this to the unique muscle attachment in the group. The *m. adductor mandibulae externus* (MAME) in hummingbirds is situated at the extreme posterior of the jaw [97], presumably to allow the jaw to quickly open and close as they feed. Thus, when we loaded the finite element model, the leverage on the jaw overall was much lower than in birds with a more typical MAME placement. Thus, we contend that hummingbirds do not have strong jaws, but rather have weak jaws with muscles positioned to put them under less strain. Nectarivores in the fossil record, then, may be particularly hard to identify with FEA given the uncertain placement of muscles onto fossil taxa.

Generalists, medium and hard invertivores, piscivores, and tetrapod hunters all have consistently weak jaws. They all plot in the upper right of PCA (Fig. 8A, B) and close to the origin in LDA (Fig. 8C, D), the areas of weakest jaws. Generalists and tetrapod hunters seem slightly stronger than the other three, never reaching the same values of MWAM strain ($\overline{\mathrm{x}}$ = 180 and 190 με respectively) as medium ($\overline{\mathrm{x}}$ = 230 με) and hard invertivores ($\overline{\mathrm{x}}$ = 240 με) or piscivores ($\overline{\mathrm{x}}$ = 220 με; Fig. 7) and plotting slightly farther from the origin in LDA (Fig. 8C, D). Generalists presumably have stronger jaws due to more frequent consumption of one of the foods listed above, while tetrapod hunters still need to tear apart prey as scavengers do but are working with softer and less tough meat. Of the remaining specialists, all of these foods taken are neither hard (compared to seeds and nuts) nor tough (compared to leaves or fruit rind), so relatively weak jaws are to be expected. Thus, it appears that while FEA is ineffective at discriminating between hard and tough diets for birds, it is effective at isolating diets that are neither hard nor tough. There is some nuance which bears discussing, however. Why piscivores would feel less selection towards strong jaws than tetrapod hunters is not immediately clear. We suspect this comes from a combination of piscivorous birds often taking smaller prey (relative to predator size) than tetrapod hunters, and the lessened ability of fish to struggle when removed from the water by a piscivorous bird, making disassembly easier. There is also the question of why some medium invertivores have unusually strong jaws (plotting at an origin distance similar to tetrapod hunters). We note that these are mainly swifts (Apodidae) and jacamars (Galbulidae), families known for flying fast and catching insects in mid-air (“hawking”) [98]. These taxa may require a stronger jaw to withstand the high-speed, high-force action of hawking capture.

While the FEA results for extant birds are rather complicated, the diagnosis for longipterygids is relatively simple. Longipterygids have consistently weak jaws, with MWAM strain greater than most groups ($\overline{\mathrm{x}}$ = 290 με, Fig. 7) and plotting in the weakest region of PCA (Fig. 8A, B). They also remain in the weak region of LDA with semi-specialists excluded (Fig. 8C), but (presumably due to a quirk of the LD2 loadings) the small-toothed *Longipteryx* enters a quite strong region of the space with semi-specialists included. LDA and DAPC predictions (Table 6) find all longipterygids likely to be piscivores or hard invertivores with lower likelihoods of being generalists, tetrapod hunters, or medium invertivores (presumably due to the stronger-jawed hawking-adapted forms mentioned above). *Longirostravis* and *Rapaxavis* have a lower but consistent likelihood of being swallowing granivores as well. There are a few odd inconsistencies: *Longirostravis* and *Rapaxavis* are recovered as likely to be folivores with semi-specialists included, but unlikely to be folivores with them excluded. Large-tooth *Longipteryx* is considered extremely likely to be a nectarivore with semi-specialists included, but unlikely with semi-specialists excluded. We regard these inconsistencies as inconclusive. Overall, FEA finds the jaw strength of all longipterygids to be most consistent with hard invertivory and piscivory. The jaws of *Longirostravis* and *Rapaxavis* are also consistent with swallowing granivory in strain-space, though their MWAM strain (280 and 380 με, respectively) is higher than any studied swallowing granivore (max 270 με).

### Longipterygid ecology and evolution

We reconstruct longipterygids other than *Shengjingornis* (whose skull is too fragmentary for reconstruction and whose claws are indeterminate in morphology) as likely to be invertivorous or generalist feeders. This is because while FEA reveals they all have weak lower jaws (consistent with invertivory, generalist feeding, piscivory, and tetrapod hunting), low body mass estimates make vertivory (and thus piscivory and tetrapod hunting) unlikely in longipterygids. While MA and functional index results are less conclusive than the other lines of evidence, they are consistent with this dietary reconstruction as well, particularly in that they also find invertivory and generalist feeding the most likely diagnoses.

**Fig. 9:**
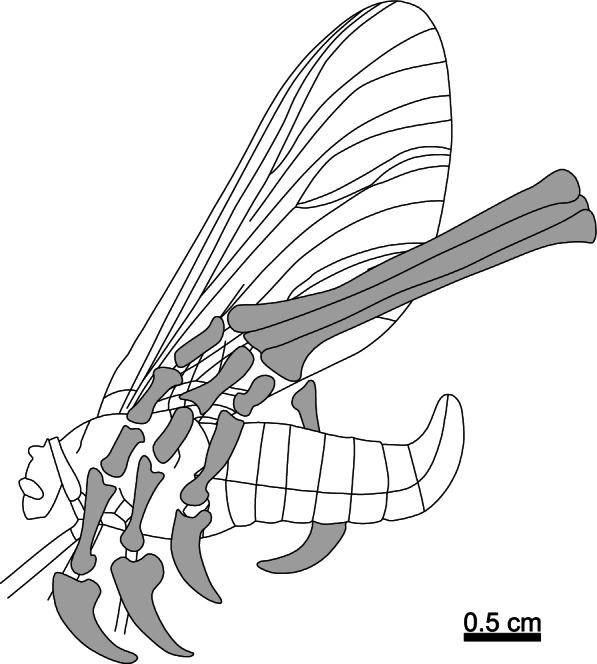
Reconstruction of the pes of large-toothed morphotype of *Longipteryx* gripping the mayfly *Epicharmeropsis hexavenulosus* from the same geological formation. Note that the mayfly is just large enough to be completely encircled by *Longipteryx*’s toes, as is typical for prey of extant owls [24]. The hindwing of *E*. *hexavenulosus* is excluded to better show the position of digit I. *Longipteryx* pes redrawn from specimen DNHM-D2889 [16], *E*. *hexavenulosus* redrawn from specimen CNU-E-YX-2007004 [99]

Using TM, we find that owl-like raptorial behaviour, where the talons are adapted to completely encircle the prey, was likely in *Longipteryx* and *Rapaxavis*. As explained above, this affinity seems to more represent the size of prey taken rather than the manner of killing per se. Owls usually hunt animals that their talons can fully encircle [24], and some of the large insects of the Jehol Biota [99] would be exactly this size for *Longipteryx* (Fig. 9). It is common for extant small owls [100], falconets [101], and shrikes [102] to capture insect prey with the hindlimb and use the pes for manipulation during disassembly of insect prey.

We find the hypothesis that *Longipteryx* is a specialist piscivore [15, 16] to be unlikely, mainly on grounds that all longipterygids are smaller than extant piscivores. It is noteworthy that the one kingfisher in our dataset, *Alcedo atthis*, does fall within the body mass range of longipterygids [103], and longipterygid piscivory has specifically been proposed as similar to that of kingfishers [13]. However, we still consider specialised piscivory unlikely for two reasons. First, the jaw of *Alcedo atthis* is stronger than that of any longipterygid, with MWAM strain comparable to hawking invertivores (160 με in *A. atthis*, minimum 260 με in longipterygids; Fig. 7). Second, *Alcedo atthis* is unusual among kingfishers in relying so much on fish. Only 15% of kingfishers have fish as the majority of their diet versus 60% of kingfishers with invertebrates as the majority of their diet [40]. So longipterygid jaw strength renders kingfisher-like behaviour unlikely, and even if this were not the case, simply being kingfisher-like would not be a strong argument for piscivory.

Swallowing granivory is recovered as likely for *Longirostravis* and *Rapaxavis* by FEA intervals (Table 6) and is consistent with their small body masses. However, we do not consider swallowing granivory likely in these taxa. The MWAM strain of their jaws (280 and 380 με, respectively) are higher than any swallowing granivores analysed (max 270 με; Fig. 7C, D). Additionally, two qualitative factors render swallowing granivory less likely in these taxa. Firstly, swallowing granivory in extant birds is aided by the gastric mill, which is believed to be absent in enantiornithines [3]. Secondly, elongated rostra would seem, if anything, to make it more difficult to channel whole seeds to the throat. This assumption is supported by past research finding granivorous birds to generally plot in morphospace regions without elongate beaks (fig. 4 in [18]).

The diet of *Shengjingornis* remains obscure, largely due to its skull being so poorly preserved. Its body mass falls in the indeterminate range for carnivores and herbivores and its unguals are undiagnostic. *Shengjingornis* is much larger than other longipterygids, appears to have a different shaped rostrum, and has an unusually reduced DIII ungual. This suggests that it inhabited a niche distinct from other longipterygids, though what this niche may have been remains unclear.

Diet appears rather conservative in Longipterygidae, with all taxa studied most adapted for invertivory or generalist feeding (except for *Shengjingornis*, whose diet is indeterminate). This conservation is consistent with previous propositions that adaptations for taking easy-to-acquire foods are ancestral in Avialae [104]. Avialans are generally reconstructed as generalist mid-order consumers in Jehol ecosystems [10], and our results do not contradict this. This contrasts with extant avians, which serve as the full range from primary to apex consumers across the clade [35] and may even do so within a single ecosystem [105]. It should be kept in mind, however, that the necessity of incorporating elements of other members of the family into reconstructions (see “Methods”) may be artificially increasing their similarity in analyses that use them (MA and FEA). However, the small-toothed morphotype of *Longipteryx* is the only reconstruction with elements from another taxon that affect both MA and FEA, so the consistent similarity of taxa seems unlikely to be driven by this factor.

If invertivory is ancestral to Enantiornithes, as has been suggested ([2] pg. 191), propositions that rostral elongation in Longipterygidae represent a trophic specialisation [11, 15] are called into question. Rostral elongation may have increased longipterygid feeding efficiency on specific families of invertebrates, creating a sub-niche specialised in feeding on that group or using it as a fallback food [106]. Longipterygids first appear during a time when insects diversified in the Jehol Biota [107], so specialisation may have been in response to this. The most likely insects that were eaten by longipterygids are mayflies (Ephemeroptera), phantom midges (Chaoboridae), or shore bugs (Saldidae) as these groups dominate the insect assemblages in the Jehol Group (Yixian and Jiufotang formations) [107]. The large size disparity between *Longipteryx* and other longipterygids (Table 7) may represent niche partitioning among longipterygids and parallel size disparities in coeval insects, e.g. large longipterygids pursuing the larger Jehol mayflies (~25mm long [99]) and smaller longipterygids the smaller phantom midges (~10mm long [108]) or shore bugs (~5 mm long [109]). Pedal morphometric differences, with *Shanweiniao* distinctly terrestrial, *Longipteryx* distinctly raptorial, and *Rapaxavis* somewhere in between, also support this picture of niche partitioning. Alternatively, rostral elongation may be unrelated to foraging at all, serving some heretofore unproposed purpose. Possibilities include sensation, thermoregulation, or display [1]. Multiple examples of feather ectoparasites are known from similar ecosystems in Myanmar during this time period [110, 111], and one could see an elongate rostrum aid in removing ectoparasites on the posterior or in elongate rectrices [11]. However, these alternatives are purely speculative and would require further investigation to be seriously considered as alternate hypotheses to dietary specialisation.

### Effectiveness of the framework

The quantitative framework we created in [1] is effective at parsing the diet of extant birds and greatly narrowing the dietary possibilities of fossil birds, even excluding isotope, microwear, and cervical muscle data. We found body mass to be highly predictive of bird diet, as in previous studies [18, 19], though not very specific in its predictions. TM of unguals is most effective at separating ground birds from other categories, but raptorial birds pursuing large prey do inhabit a mostly distinct region of the morphospace, overall consistent with [24]. Contrary to [24] and consistent with several other avian TM analyses [23, 25], we do not find differentiation of striking and restraining raptorial styles nor between suffocating raptorial style, perching, and scavenging birds in the morphospace. MA and functional indices generally did a poor job at discriminating between diets in our study. It may be that the avian skull is not primarily selected for dietary mechanical efficiency, as suggested previously [18], though it is also possible the selection is more pronounced in the lower jaw [31, 112]. Future works may wish to investigate this system instead. FEA appears capable of separating birds whose diets are hard (difficult to initiate cracking), tough (where initiated cracks do not readily propagate), or both hard and tough from those whose diets are neither hard nor tough but not, as expected [1], those with hard diets from those with tough diets. This is still very helpful, particularly as dental microwear is expected to delineate between hard and tough foods [1, 113]. The validity of MA and FEA results is contingent on reconstructions of fossil avialan skulls being accurate, a process which itself incorporates many uncertainties which need to be taken into account [114, 115]. In our study, phylogeny has a significant effect on all proxies except FEA (Table 3). We traced the nature of this effect with the *K*_mult_ statistic and by mapping phylogenies onto multivariate spaces of the proxies. We used these tools to determine that diet or pedal ecology better explained the observed trends than phylogeny. We recommend applying these methods to future studies of diet and pedal ecology to account for the effect of phylogeny on the results. The importance of synthesising results from multiple lines of evidence is also emphasised, as each diet-focused approach narrowed possibilities much less (6 possibilities for body mass, 10 for MA, 5 for FEA) than their consensus (3 possibilities, with hawking strategies ruled out for medium invertivory). Overall, this subset of the framework proposed in [1] is effective at delineating bird diet, though MA methods in particular would benefit from further refinement.

## Conclusions

We reconstruct the diet of longipterygid enantiornithines using 4 lines of evidence: body mass estimation, traditional morphometrics of pedal unguals, mechanical advantage and functional index analysis of upper jaws, and finite element analysis of lower jaws. These provide detailed evidence that refines the dietary reconstruction of four of the six published longipterygid genera. This shows that the long-snouted and rostrally toothed longipterygids retained a conservative diet likely dependent on invertebrates and/or generalist feeding. The Jehol Biota had a speciose and ecologically diverse invertebrate fauna [107], so the unusual rostral morphology of longipterygids still may represent dietary specialisation, as previously proposed [11, 15–17], to take advantage of a particular subset of the invertebrate fauna. The greater disparity in longipterygid body mass and pedal morphology may also represent subdivision of the invertivore or generalist niche. This is consistent with past reconstructions of avialans as mid-order consumers in Jehol ecosystems [10], and distinct from the extant breadth of avian trophic levels [35].

This study increases our knowledge of non-avian avialan diet by nearly 20% and triples the number of quantitatively supported enantiornithine diets. It lends credence to the hypothesis that enantiornithine birds were largely invertivorous ([2] pg. 191), and it is possible that enantiornithines’ successful radiation parallels widespread invertivory in Aves [35, 40]. We would thus predict future work to recover invertivory as the ancestral enantiornithine diet, and for invertivory to be less common outside of Ornithothoraces (as evidence already seems to show [1]). These data will prove invaluable in future analyses of avialan dietary evolution and palaeoecology and demonstrate the framework proposed in [1] to be an invaluable tool in collecting palaeodietary information. The revised methodologies herein will doubtless be useful in future studies of avian ecomorphology as well, particularly those with a morphometric [19, 24] and/or mechanical [18, 86] focus.

## Methods

### Taxonomic reference

Within the main text of this paper, we refer to extant taxa based on their genus and species in the Birds of the World database for consistency [116]. Within data files, taxa are noted based on the data source (Skullsite Bird Skull Collection [117] or museum specimen designation). We note via comments in data files where these identifications differ from Birds of the World or the bird diet database EltonTraits 1.0 [40]. Designations and relationships of fossil clades are based on [4].

### Phylogenetic tree topologies

When phylogenetic trees were used in this study for phylogenetic mapping or phylogenetic correction, the avian portion was taken from birdtree.org [118]. The tree in [118] is time-scaled using Bayesian uncorrelated relaxed molecular clock data from 15 genes in 6663 extant bird species constrained by seven fossil taxa. Longipterygid branches were then grafted onto this tree following the topology of [4] (*Shengjingornis*, not included in their phylogeny, was assumed to form a polytomy with the [*Rapaxavis* + *Longirostravis*] clade as it was recovered sister to the latter in the describing study [13]). We chose to place the Ornithothoraces node at 131 Ma given the age of the oldest known ornithothoracine taxa [119], though the taxa this old are diverse enough that the split likely occurred earlier. The oldest longipterygid, *Shanweiniao*, is known from the Dawangzhangzi Beds of the Yixian Formation [11], formed approximately 122 Ma [120]. The most recent possible age of the Longipterygidae node was estimated using this datum. All species were placed at the age of their oldest discovery with species divergences taking 10,000 years. All grafted lengths were scaled linearly so that the total length of the avian portion of the tree was equal to 94 Ma after the estimate of [121].

### Specimen selection

#### Extant skulls

Extant skull specimens (used in MA and FEA) were primarily taken as images in lateral view from the repository Skullsite. Cameras and focal lengths used to take photos on Skullsite vary (Jan Jansen pers com. 2021), though from our sampling roughly half the images have exif data with camera type and settings embedded. Generally, birds with known diets from EltonTraits 1.0 were searched for on Skullsite, and those with good lateral images (judged as those with very little of the dorsal surface of the skull showing) were used in the study. Several additional radiographs were taken from the literature [122–126], primarily as tests of whether realistic modelling of rhamphotheca thickness affected the results. Coraciiform radiographs mentioned but not pictured in [126] were provided by Kathryn C. Gamble. Finally, a CT scan (Nikon Metrology XT H 225 ST, 130 kV, 400 mA) of the mandible of *Anser fabalis* was obtained from Bjarnason and Benson [127] to help cover for the underrepresentation of Anseriformes in the dataset (only a lower jaw was available so it is not included in MA analysis). All skull specimens are adults except that of *Mitu tuberosum*, for which only a juvenile specimen was available and which has no record of ontogenetic diet shifts [128]. In total, this study incorporates skulls from 121 extant taxa.

#### Extant claws

Extant claw ungual specimens used in TM were measured and photographed in person using a Google Pixel 2 cell phone at Carnegie Museum of Natural History and Florida Museum of Natural History from their skeletal bird collections. All claws measured lacked a keratin sheath to allow comparison to ungual bones of fossil taxa. As predation or lack thereof is the main focus of this approach, sampling focused primarily on traditional raptors (Accipitriformes, Strigiformes, Falconidae) and birds known to be macrocarnivorous but not traditionally considered raptorial (Cathartidae, Laniidae, Malaconotidae, Vangidae, Aegypiinae, Gypaetinae). Parrots (Psittaciformes) were also favoured in sampling due to their talons’ superficial resemblance to those of traditional raptors, to avoid false positives for raptorial behaviour. Several other non-carnivorous perching taxa (Cuculidae, Musophagidae, *Opisthocomus*; all of which primarily live and feed in trees) and ground birds (Alaudidae, Gruidae, Megapodiidae, Tinamidae; all of which primarily live and feed on the ground) were sampled as well. All claws were checked for unusually high porosity, a pathological state common in captive birds with improper flooring (David Steadman pers. com. 2020; see also [129]). In total, the study incorporates claws from 61 extant taxa. Nine of these are from the same species as a sample in the skull dataset, though none are from the same specimen.

#### Fossils

Published longipterygid specimens were incorporated as scale photos from the literature [11, 13, 15–17, 114, 130]. Skull reconstructions used in MA and FEA (Fig. 1) can only be to approximate scale because they are composites that combine several individuals/species of different sizes, so unscaled photographs from [82] were also used in their creation. All data taken from the skull are size-independent so this should not present analytical issues. Claw measurements for the large-toothed morphotype of *Longipteryx* (see Longipterygid skull reconstruction for MA and FEA), *Rapaxavis*, *Shanweiniao*, and *Shengjingornis* were taken from scale photos from the above literature as well. Two previously undescribed specimens of *Longipteryx* sp. from the Shandong Tianyu Museum of Nature (STM 7-156, STM 8-117) were included in body mass measurements with one (STM 8-117) also providing claw measurements, though their skulls were too poorly preserved to aid in skull reconstruction.

#### Diet assignment

Bird diet was assigned based on the EltonTraits 1.0 database [40], a database recording the diet of over 9000 bird species broken down into different food categories in intervals of 10%. We then placed birds with over a certain threshold percentage of their diet from one category (Table 1) into qualitative diet categories that are similar to but more specific than those in EltonTraits 1.0. EltonTraits 1.0 tallies endotherm and ectotherm tetrapod contributions to diet separately, but due to previous studies finding no difference in hunting requirements for the two [131, 132], we merged them. The “Unknown Vertebrate” category from EltonTraits 1.0 was split evenly between tetrapods and fish (this only affected *Chionis minor* and *Grus japonensis*. *Chionis minor* was reassigned as a scavenger due to the Birds of the World database, on which EltonTraits 1.0 is based, noting that vertebrates were kleptoparasited rather than hunted). For convenience, we refer to the group which feeds primarily on non-reproductive plant tissue (“other plants” in EltonTraits 1.0) as “folivores”. While folivory refers specifically to the consumption of leaves, non-reproductive tissues of plants universally are low in nutrients and contain defensive chemicals or structures that should lead herbivores to adapt similarly to consuming them [133]. The arguable exception are the energy-rich tubers which are defended structurally and chemically [134], but are only consumed by ducks to our knowledge [135]. The only bird which consumes tubers in our dataset is *Anser fabalis* [136].

To ensure dietary signal would be as clear as possible, high thresholds were set for diet assignment. No single cut-off could be used, however, as some dietary categories tend to have a stronger domination of the primary food type than others (e.g. granivores often subsist entirely on seeds while piscivores typically supplement their diets with foods other than fish). Some species were marked as “semi-specialists” if a lower percentage of their diet consisted of the relevant food source than most members of the group, but their inclusion increased the phylogenetic breadth of the group dramatically. Generally, semi-specialists were allowed an additional 20% of their diet to be from other food sources, but expanding this to 30% in invertivores allowed inclusion of Strigiformes and expanding to 50% in scavengers was required to include more than one taxon that was not a vulture. Seeing if trends persist when including semi-specialists helps ensure dietary results are not simply recapitulating phylogeny. Cut-offs for diet assignment are given in Table 1.

The Diet-Fruit category of EltonTraits 1.0 [40] includes food items that are very mechanically different, from soft-fleshed papaya to rock-solid palm nuts. Thus, we split this diet category into Soft Frugivores (FrugivoreS) and Hard Frugivores (FrugivoreH). While “hard” fruits have traditionally been operationalised as having a puncture resistance above about 0.25 kgmm^−2^ [137, 138], this precision is not possible with our current data on bird diet and food material properties. Plants fed on by birds are typically accurate only to the family level [116], while puncture resistance can vary by more than 0.25 kgmm^−2^ even at the genus level [139] meaning many families contain both hard and soft fruits. As a compromise, we used the Birds of the World database to catalogue the families of fruits eaten by the frugivores in this study (Arecaceae, Burseraceae, Caricaceae, Celastraceae, Fabaceae, Lauraceae, Moraceae, Myristicaceae, Myrtaceae, Oleaceae, Podocarpaceae, Primulaceae, Putranjivaceae, Sapotaceae, and Urticaceae) and identified two that are known for having particularly hard fruits (Arecaceae [140] and Myristicaceae [141, 142]). Taxa that fed on fruits from these two families were assigned to FrugivoreH, others to FrugivoreS. *Mitu tuberosum* was also assigned to FrugivoreH due to specific note of it eating fruits of *Lecointea amazonica* [143] which are on record as hard fruits [144].

The Diet-Seed category of EltonTraits 1.0 includes two distinct foraging styles: those that remove the hard outer coating of seeds (de-husk) and those that swallow them whole. These styles appear mutually exclusive among most birds [145] and require different adaptations of the skull, so we see fit to separate granivores into Granivores that Husk (GranivoreH) and Granivores that Swallow seeds whole (GranivoreS). We determined which studied granivorous taxa de-husk seeds and which do not based on notes and videos in the Birds of the World database. De-husking in *Sporophila crassirostris* is inferred from *Sporophila telasco* and was observed directly by CVM in *Nymphicus hollandicus*. Swallowing seeds in *Pterocles orientalis* is inferred from *Pterocles lichtensteinii*.

The Diet-Inv category of EltonTraits 1.0 [40] also includes foods with a wide range of mechanical properties, from fragile butterflies to rugged crabs. Bestwick et al. [146] compiled literature on the properties of invertebrate exoskeletons and defined three mechanical groups: Soft Invertebrates (invertebrate larvae, lepidopterans, spiders, and myriapods), Medium Invertebrates (orthopterans, formicid hymenopterans, and odonatans), and Hard Invertebrates (coleopterans, crustaceans, and shelled gastropods). We also assign springtails (Collembola) to Soft and flies (Diptera) and termites (Isoptera) to Medium Invertebrates based on indentation hardness of their body parts compared to the above groups (compare [147–149]). Krill are very tentatively assigned to Medium as they lack the calcified shells found in other crustaceans ( [150], but see [151]). Using these divisions, we split invertivores into InvertivoreH, InvertivoreM, and InvertivoreS depending on the hardest group the bird consumes according to the Birds of the World database and references therein. We split them using the hardest group consumed as birds not sufficiently adapted to the hardest item they regularly consume would experience frequent failure in feeding and be at an evolutionary disadvantage.

Some specialised feeding styles require further explanation. *Rostrhamus sociabilis* feeds on shelled gastropods but does not consume their shell, instead pulling the snails out through the shell aperture [152]. Because it only consumes soft portions of the invertebrate, it was coded as InvertivoreS. *Phoenicopterus chilensis* uses a unique form of filter feeding in which tiny organisms are sieved from water and sediment and then swallowed [51]. Because no crushing of the prey takes place in the jaw, we code the taxon as InvertivoreS, but realistically the stresses of sieving sediment in the mouth likely impose pressures on *Phoenicopterus* unique from most avian taxa. *Pelecanoides urinatrix* also feeds principally on planktonic crustaceans, and so is coded as InvertivoreS for the same reason. It is worth noting that, because most invertivores take a variety of invertebrates, these three specialists are the only taxa assigned to InvertivoreS.

Kruuk [153] defined three convergent feeding guilds for vultures (which make up the majority of our Scavenger diet), each of which feeds on carrion in a distinct manner. We included a mixture of guilds to get the full range of scavenging behaviour: two rippers (vultures which initially tear open carcasses; *Aegypius monachus* and *Sarcoramphus papa*), one gulper (vultures which swallow soft viscera; *Gyps ruppellii*), and one scrapper (vultures which glean small bits of meat in and around a carcass; *Cathartes aura*) [90, 91]. Also included is *Gypaetus barbatus*, whose skull strongly resembles those of gulpers [90] but whose postcranial skeleton is more similar to rippers [91] (though it generally occupies a unique region of the morphospace (fig. 3 and 4 in 91)).

Occasionally, birds are categorised using more inclusive dietary categories in order to make graphs easier to read. Herbivores include folivores, frugivores, granivores, and nectarivores. Carnivores include invertivores, piscivores, scavengers, and tetrapod hunters. For our purposes, omnivores and generalists are synonymous. These newly defined dietary categories allow for greater precision in identifying bird diet than the previous five category system used in EltonTraits 1.0 [40] and most studies based off of it, while also having clear quantitative definitions building upon the highly specific categories of Lopes et al. [154].

In total, this study includes the following: eight folivores, three hard frugivores, four soft frugivores, 17 generalists, five husking granivores, six swallowing granivores, 31 hard invertivores, eight medium invertivores, three soft invertivores, six nectarivores, 13 piscivores, eight scavengers, and nine tetrapod hunters.

#### Body mass

Body mass estimation for the fossil specimens follows the measurements of [21], with the revisions to the regression equation noted in (table 2 in [1]):

$$\mathrm{ENAN}:-2.626+1.528\ \mathrm{HL}+0.34\ \mathrm{bcL}+0.828\ \mathrm{dHW}-1.451\ \mathrm{UL}+0.811\ \mathrm{dUW}+0.378\ \mathrm{TL}$$

Body mass correction factors were back-calculated from [21]; all were very close to 1. See [21] for diagram of landmarks for measurements. Abbreviations are given in full at the end of this work.

Prior to the current study, body mass estimates for the holotypes of *Longipteryx chaoyangensis* and *Longirostravis hani* were made from direct linear measurements [21], and estimates for *Rapaxavis pani* [21], *Shanweiniao cooperorum*, and an additional specimen of *L*. *chaoyangensis* [1] were made from scaled photographs. In this study, two previously undescribed specimens of *Longipteryx* sp. from the Shandong Tianyu Museum of Nature (STM 7-156, STM 8-117) were also measured and their body mass estimated using the ENAN regression equation in [1]. These calculated masses are provided in Table 7.

Average adult body masses for extant birds were taken from [103]. Taxa investigated were restricted to the 120 used in MA, as we had already ensured their diet information was accurate. When masses for males and females were given, these were averaged (sex data is not recorded in EltonTraits 1.0 or Skullsite). When masses for multiple subspecies or populations were given, these were averaged weighted by the number of samples for each (assuming this paralleled their natural abundance). When only maximum and minimum masses were provided, we assumed a mean mass as their midpoint. These averaged masses were used in subsequent analysis. All calculations were made on Log_10_-transformed mass as is standard [155]. Mass for *Chalcopsitta duivenbodei* was based on *Chalcopsitta scintillata* and mass for *Sitta azurea* was based on *Sitta pusilla*.

### Traditional morphometrics

#### Ecological category assignment

Ecological categories for raptorial birds and their assignment generally follow [24]: Restraint—hawks and eagles (Accipitridae) use their talons for prolonged prey restraint while they kill large prey slowly; Strike—falcons (Falconini) strike large prey concussively with their feet before killing quickly with their jaws; Suffocation—owls (Strigidae) are specialised to suffocate small prey within their toes, mainly using talons to extend their reach; Pierce—ospreys (*Pandion*) pierce their talons into fish to aid in gripping as they extract them from the water. We sought to broaden the membership in these categories to better account for phylogenetic constraint. Forest-falcons (*Micrastur)*, while members of Falconidae, will feed on large pinned prey while it is still living ( [156]; Dorival Lima, Falcoaria e Voo Livre, pers. comm. 2021) akin to hawks and eagles, so we assigned this genus to the restraint category. Shrikes (Laniidae) and Helmetshrikes (Vangidae) are both placed in the restraint category due to their prolonged manipulation and transport of prey prior to impalement [68], though it should be noted that large prey may be paralysed or killed without the use of the feet prior to impalement [71, 102]. Secretarybirds (*Sagittarius*) and seriemas (Cariamidae) are both well-known for kicking with their hindlimbs, and so were added to the strike category. It should be noted, however, that while secretarybirds use kicks as their main hunting strategy [157], seriemas mainly forage with their beaks and kick during interspecific combat [158].

Among non-raptorial birds, acquisition of food with the pes is rare and manipulation of it is limited [159], so non-raptorial ecological categories characterise habitat rather than diet. Birds in the Ground and Perching categories are somewhat self-explanatory: birds that spend the vast majority of their time on the ground or perched on a branch, respectively. We strived for a broad phylogenetic breadth for each group: tinamous (Tinamidae), megapodes (Megapodiidae), cranes (Gruiformes), and larks (Alaudidae) represent ground birds; hoatzins (*Opisthocomus*), cuckoos (Cuculidae), turacos (Musophagidae), and parrots (Psittaciformes) represent perching birds. We separated out new-world vultures (Cathartidae) as Scavengers to see if this lifestyle had any diagnostic characteristics in the talons, despite their talons plotting with ground [24] or perching [43] birds in past analyses.

In total, this study includes nine ground birds, 14 non-raptorial perching birds, one piercing raptor, 15 restraint raptors, four scavengers, eight striking raptors, and ten suffocating raptors.

#### Measurements

Linear measures of extant claws and tibiotarsi were taken with a tape measure to avoid damaging the fragile specimens, with callipers (digital at Carnegie, dial at Florida due to technical difficulties) used on any claws less than 1 cm in proximodistal length. Angular measures were taken from photos taken in lateral view imported into CorelDraw X8 and measured as in [24] using the “Angular Dimension” tool. If digit identifications were ever in doubt, the claws were compared to taxidermy specimens and identified based on relative size and curvature.

Landmarks for TM follow [24] with two deviations. We figure these landmarks in a previous work (fig. 1 and 2 in [1]). First, the bone cores of claws were measured rather than keratinous sheaths. This allowed direct comparison to fossils which rarely preserve an outline of the keratin sheath. Even if an outline of the keratinous sheath was preserved in a fossil, it could be deformed with no indication of the original structure [160]. Measuring bone cores obscures inner-curvature landmarks (based largely on the transition from the flesh to the claw sheath) so we affirm the use of outer-curvature landmarks (i.e. the proximal landmark at the dorsal base of the extensor tubercle) as has become standard in avian pedal TM [24, 36, 64, 161]. Second, use of extant skeletal specimens usually prohibits inclusion of toe lengths, as toes are disarticulated with no reliable way to be reassembled in extant skeletal specimens. Reliance on toe measurements would also exclude entire groups of fossil avialans from future study (most notably Avisauridae [162]), so we see this limitation as a necessary eventuality when studying fossil bird diet. Thus, the final measures used in TM were outer arc curvature (Oo) and ratios of outer arc length (ALo) (*sensu* [24]) for each digit.

As angle measurements were taken from photos, we tested the effect of parallax by comparing a perfectly level photograph to those taken at 5° tilts in each orthogonal direction. 5° was considered the maximum reasonable deviation from level, as the camera had an accelerometer-based level whose crosshairs decouple near 5° of tilt. Parallax was found to have little effect, consistent with previous studies [163]. We also took photographs of a grid with the camera and affirmed the gird lines remained orthogonal.

Both digit III (DIII) and digit IV (DIV) were investigated as reference digits, i.e. as the denominator of size ratios. LDA models based on each were almost identically accurate when re-classifying extant taxa (Fleiss’ Kappa = 0.7177 DIII, 0.7182 DIV; see “Multivariate” analysis). Visual comparison of PCA plots found better separation when using DIII as a reference digit, so DIII is used in this paper. Graphs of results where DIV is used as a reference digit are available in Additional file 1: Fig. S6.

### Longipterygid skull reconstruction for MA and FEA

Final longipterygid skull reconstructions are pictured in Fig. 1. Because no longipterygid skull is complete, extrapolation of bones was necessary to create reconstructions. While not ideal due to intra- and interspecific morphological variation, this practice is common and necessary to create workable biomechanical models [115]. The ontogeny of enantiornithines remains largely uncertain so ontogenetic effects cannot be fully accounted for, though juvenile enantiornithines tend to have more gracile long bones and relatively larger orbits than any specimen used in this study [164]. *Longipteryx* specimen IVPP V12552 has been proposed as a juvenile due to several of its bones being unfused [76], though subsequent work has shown that the pattern of skeletal fusion in enantiornithines is highly variable [165]. Previous work reconstructed enantiornithine skulls based on the general morphology at the level of Enantiornithes [114]. We sought to improve on these reconstructions by leveraging newly published specimens, restricting extrapolated material to the family level (Longipterygidae), and making explicit what areas are reconstructed and where extrapolated material comes from.

Published images [2, 11, 13, 15, 16, 76, 82, 114, 130] were imported into CorelDraw X8. All skulls studied are preserved in lateral view. Skulls were then scaled to all have the same length (from tip of the rostrum to rear of the cranium). If a skull was disarticulated, its best-preserved bone was scaled to the same size at its closest phylogenetic relative per [4]. Once scaled, each distinct bone or set of bones (e.g. premaxilla + nasal with no clear suture preserved) in each skull was outlined and named according to its identification and source specimen. In every specimen, most individual bones of the cranium were indistinct, so a general “cranium” outline was made as well. The articular region between the upper and lower jaws was not clear in any studied specimen of *Longipteryx*, so descriptions from [84] were used to refine this area.

The most complete skull of a given genus was used as the base for reconstruction. Copies of its bone outlines were made and isolated, with upper and lower jaws moved into articulation. Missing or incomplete bones were then taken from the closest relative preserving the bone. These outlines were copied as well and placed between or over top of the existing bones, attempting to meet articulated bones cleanly and align with as many edges as possible of overlapping bones (akin to the process [115] recommends for 3D reconstruction). Once complete, new outlines were made by tracing over the composite of bones to make edges and articulations cleaner. If bone edges overlapped, those of the genus being reconstructed were favoured over its relatives, and among relatives those with the best preservation were favoured. Sutures were not intuited in bone sets so as to not overestimate the precision of the reconstruction. Finally, bones and bone sets were coloured based on the specimen they came from. Bones or bone sets that are amalgams of multiple specimens were given gradient fills approximating the regions with greatest contribution from a given specimen.

The sclerotic ring and lacrimal are only preserved in *Longipteryx* (BMNHC Ph-930B), the earliest-diverging member of the clade. Reconstruction of the sclerotic ring followed avian examples in [166], from which it appears shape in lateral view is conserved phylogenetically. We therefore believe the shape and arrangement of ossicles in our reconstructions are adequate, though their size and placement (based solely on BMNHC Ph-930B) are only tentative. The lacrimal is positioned in each skull as connecting the dorsal process of the maxilla to the frontal (except in *Rapaxavis*, where the maxillary process nearly contacts the dorsal premaxilla and the frontal begins more cranially than in other longipterygids), as in BMNHC Ph-930B. This often required removing one or both ends of the lacrimal as it appears in BMNHC Ph-930B and significant slimming of the bone for it to be similar in aspect ratio to the other bones in longipterygid skulls other than *Longipteryx*. As such, we note our reconstruction of the lacrimal as highly speculative. Additionally, the quadratojugal is not distinct in any enantiornithine except for *Dapingfangornis* [167] in which it is a short cranially forked rod of bone contacting the jugal, quadrate, and squamosal, so we incorporate a similar morphology into these reconstructions using a dotted line. These bones do not affect any measurements taken so their uncertainties have no effect on any quantitative analyses in this work.

The position of the quadrate in enantiornithines is highly uncertain. An in situ quadrate has only been pictured in *Pengornis houi* [114], and while the quadrate of *Longipteryx* specimen IVPP V21702 is reported as articulated only an extreme close-up of the quadrate is figured [84]. We believe the cranial structure abutting the surangular in *Longipteryx* specimen BMNHC Ph-930B is also an in situ quadrate, and so use its positioning in this study. BMNHC Ph-930B preserves the orbital process of the quadrate as a broad anterior projection, common among non-avian avialans [84], so this feature is assumed to be present but unpreserved in other longipterygids. The quadrate is situated more cranially in BMNHC Ph-930B than in *Pengornis* and past skull reconstructions [114], which we attempt to replicate in other longipterygid skull reconstructions. This is attenuated by the depressions in the surangular of *Rapaxavis* and *Shanweiniao* which require the quadrate to be more rostrally positioned. As such, when placing the quadrate, we aligned the front of the upper and lower jaws and situated the quadrate as cranially as reasonable while still articulating with the surangular. As the articular condyle (located on the quadrate) is a landmark for every type of jaw mechanical advantage, this placement should, in theory, heavily influence the results of mechanical advantage calculation. However, a sensitivity analysis placing the articular condyle at biologically improbable cranial and rostral extremes (Additional file 1: Fig. S8) found dietary assignments to be overall robust to quadrate position (Additional file 1: Table S6).

In our reconstruction work, we noticed two distinct morphotypes of *Longipteryx chaoyangensis*: those with large teeth and more robust jaws (BMNHC Ph-930B, DNHM D2889, HGM-41HIII0319, SG2005-B1) and those with smaller teeth and more gracile jaws (BMNHC Ph-826, IVPP V12552, possibly BMNHC Ph-1071 and IVPP V12325). The difference may be interspecific or ontogenetic (IVPP V12552 has been identified as a subadult [2], but see above), but in this study, we err on the side of caution by classifying the two as “large-toothed *Longipteryx*” and “small-toothed *Longipteryx*”, since this difference requires further study. In phylogenetic trees, these morphotypes were given the smallest branch length possible [10] for their divergence.

### Mechanical advantage and functional index calculation

As all known longipterygid fossils are only preserved as slab specimens, MA and functional indices are measured in two dimensions as well. Measurements were made on the upper jaw due to [168] finding the upper jaw to have a stronger influence on overall MA than the lower jaw. Terminology follows [31]: anterior jaw-closing mechanical advantage is abbreviated AMA, posterior jaw-closing mechanical advantage PMA, jaw-opening mechanical advantage OMA, and relative articular offset AO. Maximum cranial height is abbreviated MCH and average cranial height ACH, equivalent to MMH and AMH in [31]. A diagram of these modified measurements are provided in fig. 4 in [1] on an enantiornithine and in Fig. 2 on an avian.

Differing from [31], for AMA and PMA the inlever is calculated as in [18, 168, 169] as the perpendicular distance between the line of action of the jaw adductor muscles and the articular condyle (most notably because attachments are more spread-out and more uncertain on the upper jaw than the lower jaw). Because the *m. depressor mandibulae* attaches to the jaw over a relatively small lateral area, the distance from its attachment point to the articular condyle is used as the inlever for OMA. All other mechanical advantage landmarks follow those of [31]. This is a noticeable divergence from past work on avians in [18]; Pittman et al. [31] define the outlever of PMA as ending at the cranialmost point of rhamphotheca in beaked specimens, whereas Navalón et al. [18] use the midpoint of the rhamphotheca ventral arc. All measurements were taken of images in CorelDrawX8 using the “Parallel Dimension” tool. While images on Skullsite are unscaled, absolute scale is unnecessary for this study because only ratios are investigated here.

When measuring skull length, to which most functional indices are normalised, length was measured subparallel to the ventral edge of the maxilla or, in Psittaciformes, the jugal. In groups with curved maxillae (most notably Cathartidae and Apodidae), the portion most parallel to the coronal plane was used. For AO in toothed specimens, the occlusal margin ignores teeth that are recessed relative to the others (e.g. the first premaxillary tooth of *Longipteryx*). For beaked specimens, a line is drawn between the rostral and cranial extremes of the occlusal surface and moved dorsally until the area ventral to the line would fill the empty space dorsal to the line (except in *Ephippiorhynchus* and *Recurvirostra*, where directions are reversed). See the note in Fig. 2D for an illustration of this.

In translating the measurements of [31], it was unclear which “heights” were appropriate to parallel average and maximum mandibular height. We took measurements of the maximum heights of the rostrum (measured perpendicular to the ventral edge of the maxilla up to the frontal/nasal contact) and whole skull (measured perpendicular to the maxilla or jugal to the most distant point of the skull), as well as the area of the rostrum (rostral to the frontal/nasal contact) and of the whole skull (excluding area ventral to the jugal, as that area is uncertain in enantiornithines) with and without holes (orbit, naris, antorbital fenestra). We then subjected extant data sets using rostral measures, cranial measures with holes, and cranial measures without holes to LDA. The resulting equations were used to re-predict extant bird diets, and the set with the greatest agreement with known diets (tested via Fleiss’ Kappa, see “Multivariate” analysis) was selected. We found cranial height and area without holes to have the strongest predictive power and used it in all subsequent analyses.

### Finite element analysis

#### Modelling

FEA comparisons are based on the principles set forth by [170], in which a structure is relatively weaker if it experiences higher levels of a failure criterion under the same relative load. We chose to use total maximum principal strain as a failure criterion rather than Von Mises stress for reasons explained in [1]. In short, total maximum principal strain has proven more effective than other criteria at predicting the force required for bone to fail and the place of bone failure in in vitro studies [171, 172] and is used in medical practice to evaluate in vivo bone strength [173]. We also chose to study birds’ lower jaws to remove cranial kinesis and suture properties as confounding variables [1]. The mandibular suture was not modelled as the material properties of bird sutures are poorly constrained [1, 174, 175] and the mandibular suture is fully fused in most adult extant birds [47, 176]. Because all longipterygid fossils are preserved in only two dimensions, all finite element models were made two dimensional. Plane strain assumptions were made for all models with relative loading for a constant strain state based on [177], making all results model-size-independent. This allows us to compare models based on their relative strength, i.e. which shape and muscle arrangement accrues the most and least strain under equal loading [170], rather than invoking a failure state. This is necessary because it would be inappropriate to make any conclusions based on failure criteria in any finite element model that has not undergone experimental validation [47]. All models used homogeneous, isotropic material properties for the skull and rhamphotheca found to produce results similar to in vitro strain gauge data in ostriches by [174] (*E* = 7000 MPa, *ν* = 0.35 for bone; *E* = 3000 MPa, *ν* = 0.35 for rhamphotheca).

Rhamphotheca thickness is not visible from external pictures and varies greatly between birds (compare [122, 123]). Sensitivity analysis (Additional file 1: Figs S9, S10, Table S7) found models with a dorsoventral thickness of 20% rhamphotheca and 80% bone to most closely mimic those built using the true rhamphotheca thickness from a radiograph, as in a previous study [178]. Final avian models were thus constructed using true rhamphotheca thickness if a radiograph was available and with a dorsoventral thickness of 20% rhamphotheca and 80% bone if unavailable.

Loads were applied using the muscle simulation method of [179] to recreate the *m. adductor mandibulae externus* (MAME). Attachment and orientation of the MAME was based on dissections and dissection diagrams of extant birds [180–195] using the phylogenetic group closest to the modelled taxon. In fossil taxa, muscles were reconstructed based on the proposed non-avian dinosaur attachment sites and orientations [196] with preference for inferences based on birds over crocodilians. When in doubt, the MAME was assumed to insert ventral to the coronoid process of the mandible (or, when coronoid process was unclear, dorsal to the mandibular fenestra) and to be oriented roughly 45° from the coronal plane, as this appears to be the most common condition in archosaurs [194]. Load magnitude at the MAME attachment point was arbitrarily chosen to be 6N for the smallest model (*Regulus regulus*). Loads for all other models were scaled from this one using the Stress State Constant/Plane Strain equation in [177]. This effectively makes model strains size-independent, allowing for comparison of the effects of jaw shape and muscle attachment in isolation. Models were constrained from translation in all axes at the articular glenoid. Models also were constrained dorsoventrally at the rostral tip of the rhamphotheca in beaked taxa and at the apex of the first tooth in toothed taxa. All models were created and solved within HyperWorks 2019 Student Edition (*HyperMesh* and *Optistruct*, Altair Engineering, Inc., USA).

#### Intervals method

To compare the outputs of finite element models in a quantitative manner, we utilise the intervals method for comparing finite element outputs [34]. Conceptually, this technique is an extension of comparing contour plots, but allows them to be much more detailed than a subjective comparison. We split the full range of strain for all models into a number of equally sized intervals (analogous to colours on a contour plot), and the percent area of each model under each interval of strain is quantified. The outcome is akin to visually comparing the area of contour plots that are certain colours, but allows us to make these comparisons in a quantitative way. Convergence testing was used to determine what number of intervals was optimal. Deviating from [34], we transformed the raw intervals data matrix as it is compositional data, which cannot be used as-is in multivariate analyses [197]. We imputed zeroes using expected value multiplicative lognormal replacement [198] with the multLN function in R package zCompositions [199] version 1.3.4 before applying an isometric log ratio transformation [200] (ilr function in R package compositions [201] version 2.0-2) to the primary use FEA data and a centred log ratio transformation [197] (clr function in R package compositions [201] version 2.0-2) to the data used for character weight plotting. Imputation is necessary as the logarithm of zero is undefined. Isometric log ratio transformation more completely removes compositional effects from the data [200], while a centred log ratio transformation makes it much easier to interpret character weightings [197]. After transformation, FEA intervals data was subjected to multivariate analysis as described below.

### Data analysis

All analyses of the data were performed in R version 4.1.2 [202], with scripts available from [203]. Additional files 2, 3, 4, 5, 6, 7, 8, 9, 10, 11, 12, 13, 14 and 15 also include interactive HTML-based three-dimensional graphs of all multivariate analysis results and two-dimensional multivariate FEA graphs annotated with contour plots of each jaw. Both were made using a package from Plotly (Plotly, Canada) for R [204], version 4.9.4.1. Univariate results in this study are compared in violin plots, a series of rotated and mirrored kernel density plots. When comparing subsets of carnivore and herbivore masses, we determined diagnostic cut-off values to compare fossil bird masses to using the R package OptimalCutpoints [205] version 1.1-5 (function optimal.cutpoints, optimised using Youden Index [44]). We performed two initial analyses on each multivariate dataset: principal component analysis (PCA; base R function prcomp) and linear discriminant analysis (LDA; caret package for R [206] version 6.0-90 function lda). Both seek to reduce dimensionality of data into a space easier to interpret, but do so by different means. PCA maximises the variance explained by each axis while LDA maximises the separation of predefined groups [197] (in this instance, diet or pedal ecological categories). In this sense, PCA can be seen as a more “objective” view of the data while LDA is more effective at identifying otherwise minor factors which distinguish groups. All PCAs in this study used the correlation matrix, which scales inputs to constant variance, removing effects of units and scale. All fossil data points were projected independently into multivariate space (i.e. they were not used in calculating the rotation of the data).

LDA has many more assumptions than PCA, the most troublesome of which is that all variables are uncorrelated. In biological systems, where mechanical traits are linked by a variety of developmental and evolutionary relationships that are often poorly understood, uncorrelated variables are difficult to isolate. As such, LDA performed in this study will inevitably defy this requirement for LDA. To account for this, we also incorporated discriminant analysis of principal components (DAPC) [46]. In essence, DAPC combines PCA and LDA into a single analysis. PCA de-correlates the input variables, and then LDA is performed on the principal components. Unfortunately, DAPC plots are very difficult to interpret. Each linear discriminant is made up of a combination of principal components, which are in turn made up of a combination of input variables. As such, we primarily use DAPC as a check on LDA. If LDA and DAPC plots of a given dataset look similar then we consider the LDA robust to the uncorrelated assumption, but if they were to differ then we would attempt to back-interpret the DAPC plot as best as possible. All LDA and DAPC outputs in this study are identical (compare relevant LDA results to Additional file 1: Fig. S2), so back-interpretation was not necessary.

When variable choice was in question (e.g. using digit III or IV as a reference digit in TM, how to measure skull area for MA, or the number of intervals to use in multivariate FEA), we compared sets in LDA using Fleiss’ Kappa [207] (obtained using caret package for R [206] version 6.0-90 function confusionMatrix). Fleiss’ Kappa is used to test the agreement of two observers in categorising data. We treated the a priori diet/ecology classification of birds used in this study as one observer and compared it to the LDA equations’ prediction of categories for said birds. Higher Fleiss’ Kappa means more agreement of the LDA predictions with reality, and thus a dataset producing more accurate results.

Extant groups with more than one member were compared in terms of TM variables (Additional file 1: Table S1), MA variables (Additional file 1: Table S3), and FEA intervals (Additional file 1: Table S5) using the pairwise() function in the RRPP package for R [39] (version 1.1.2) to test if they were distinct. In total, 1000 permutations were used by convention, with sensitivity analyses finding *p*-values to converge before this point. This test mimics the function of Tukey’s honestly significant differences (HSD) test [38] when comparing means, a nonparametric test for significant difference between each pairwise comparison within a large set of groups (in this case pedal ecological category or diet). It effectively differs from Tukey’s HSD in that it is able to incorporate and correct for phylogenetic signal in the data. As the authors of [39] did not provide a concise name for the output of the pairwise() function when comparing means, we refer to the results of this test herein as “phylogenetic HSD”.

In HSD, LDA, and DAPC of non-semi-specialist datasets for mass, MA, and FEA, *Steatornis caripensis* was removed from the analysis as it was the only representative of FrugivoreH. Single-member groups are not compatible with HSD and tend to interfere with LDA results, plotting at extreme values of a single discriminant and warping the remaining data. For the same reason, *Pandion haliaetus* was removed from phylogenetic HSD, LDA, and DAPC of TM datasets as the only representative of the Pierce category.

Phylogenetic signal was investigated in each dataset using the *K*_mult_ statistic [37] (function R code given in the paper), a summary statistic comparing the differences in high-dimensional traits across a given tree. A total of 1000 permutations were used by convention. A *K*_mult_ value of 1 indicates traits evolved under a Brownian motion model, i.e. random changes with no selection. Values less than 1 indicate taxa are more different from one another than in a Brownian motion model, values greater than 1 indicate taxa are more similar than expected [45]. The test also provides a *p*-value for the presence of phylogenetic signal (null hypothesis of no phylogenetic signal). Per the recommendation of Adams and Collyer [78] when *K*_mult_ was less than 1 but statistically significant phylogenetic signal was detected we also took *K* values for each individual input variable (Additional file 1: Tables S2 and S4). We used the same code to calculate *K*_mult_ and *K* values (the equivalent for univariate systems like body mass) as Adams [37] demonstrated *K*_mult_ = *K* for one-dimensional data.

## Supplementary Information

**Additional file 1: FigS1.** Plot of TM character weights. **FigS2.** All DAPC plots. **FigS3.** Violin plots of MA/functional indices. **FigS4.** Plot of MA/functional indices character weights. **FigS5.** Plot of FEA character weights. **FigS6.** TM phylomorphospace with digit IV as reference. **FigS7.** TM phylomorphospace with phylogeny-based hulls. **FigS8.** Longipterygid skull reconstructions for MA/functional indices sensitivity analysis. **FigS9.** Dot plot of MWAM strain for FEA sensitivity analysis. **FigS10.** Intervals method PCA for FEA sensitivity analysis. **Table S1.** TM phylogenetic HSD results. **Table S2.** K values for individual TM variables. **Table S3.** MA/functional indices phylogenetic HSD results. **Table S4.** K values for individual MA/functional indices variables. **Table S5.** FEA phylogenetic HSD results. **Table S6.** LDA predictions from MA/functional indices sensitivity analysis. **Table S7.** Comparison of MWAM strain from FEA sensitivity analysis.

**Additional file 2.** Interactive 3D PCA graph of TM data. Based on same data as Fig. 5A.

**Additional file 3.** Interactive 3D LDA graph of TM data. Based on same data as Fig. 5B.

**Additional file 4.** Interactive 3D PCA graph of MA/functional indices data, excluding semi-specialists. Based on same data as Fig. 6A.

**Additional file 5.** Interactive 3D PCA graph of MA/functional indices data, including semi-specialists. Based on same data as Fig. 6B.

**Additional file 6.** Interactive 3D LDA graph of MA/functional indices data, excluding semi-specialists. Based on same data as Fig. 6C.

**Additional file 7.** Interactive 3D LDA graph of MA/functional indices data, including semi-specialists. Based on same data as Fig. 6D.

**Additional file 8.** Interactive 3D PCA graph of FEA intervals data, excluding semi-specialists. Based on same data as Fig. 8A.

**Additional file 9.** Interactive 3D PCA graph of FEA intervals data, including semi-specialists. Based on same data as Fig. 8B.

**Additional file 10.** Interactive FEA PCA graph showing contour plots on hover, excluding semi-specialists. Based on same data as Fig. 8A.

**Additional file 11.** Interactive FEA PCA graph showing contour plots on hover, including semi-specialists. Based on same data as Fig. 8B.

**Additional file 12.** Interactive 3D LDA graph of FEA intervals data, excluding semi-specialists. Based on same data as Fig. 8C.

**Additional file 13.** Interactive 3D LDA graph of FEA intervals data, including semi-specialists. Based on same data as Fig. 8D.

**Additional file 14.** Interactive FEA LDA graph showing contour plots on hover, excluding semi-specialists. Based on same data as Fig. 8C.

**Additional file 15.** Interactive FEA LDA graph showing contour plots on hover, including semi-specialists. Based on same data as Fig. 8D.

## Data Availability

All data and computer code used in this study is stored at Mendeley Data, DOI 10.17632/w3c8p5w3hn.1 [203].

## Abbreviations

ACH: Relative average cranial height
AMA: Anterior jaw-closing mechanical advantage
ANOVA: Analysis of variance
bcL: Length of bicipital crest
BMNHC: Beijing Museum of Natural History, Beijing, China
CM: Carnegie Museum of Natural History, Pittsburgh, USA
CNU: Capital Normal University, Beijing, China
dHW: Dorsoventral width at midshaft of humerus
dUW: Craniocaudal width at midshaft of ulna
DAPC: Discriminant analysis of principal components
DNHM: Dalian Natural History Museum, Dalian, China
FEA: Finite element analysis
FolFrug: Folivores and frugivores
FrugivoreH: Hard frugivroe
FrugivoreS: Soft frugivore
GranivoreH: Husking granivore
GranivoreS: Swallowing granivore
GranNect: Granivores and nectarivores
HL: Length of humerus
HSD: Honest significant difference
InvertivoreH: Hard invertivore
InvertivoreM: Medium invertivore
InvertivoreS: Soft invertivore
IVPP: Institute of Vertebrate Paleontology and Paleoanthropology, Beijing, China
LD: Linear discriminant
LDA: Linear discriminant analysis
MA: Mechanical advantage
MCH: Relative maximum cranial height
MWAM: Mesh-weighted arithmetic mean
OA: Relative articular offset
OMA: Jaw-opening mechanical advantage
PC: Principal component
PCA: Principal component analysis
PMA: Posterior jaw-closing mechanical advantage
STM: Shandong Tianyu Museum of Nature, Pingyi, China
TL: Length of tibiotarsus
TM: Traditional morphometrics
UL: Length of ulna
$$\overline{\mathrm{x}}$$: Mean value
με: Miscrostrain
