# Supplementary material for: Diet of Mesozoic toothed birds (Longipterygidae) inferred from quantitative analysis of extant avian diet proxies
Source: BMC Biol. 2022 May 12;20:101. doi: 10.1186/s12915-022-01294-3 (PMC9097364; doi:10.1186/s12915-022-01294-3)
Supplement: Supplementary file 1 — Additional file 1: FigS1. Plot of TM character weights. FigS2. All DAPC plots. FigS3. Violin plots of MA/functional indices. FigS4. Plot of MA/functional indices character weights. FigS5. Plot of FEA character weights. FigS6. TM phylomorphospace with digit IV as reference. FigS7. TM phylomorphospace with phylogeny-based hulls. FigS8. Longipterygid skull reconstructions for MA/functional indices sensitivity analysis. FigS9. Dot plot of MWAM strain for FEA sensitivity analysis. FigS10. Intervals method PCA for FEA sensitivity analysis. Table S1. TM phylogenetic HSD results. Table S2. K values for individual TM variables. Table S3. MA/functional indices phylogenetic HSD results. Table S4. K values for individual MA/functional indices variables. Table S5. FEA phylogenetic HSD results. Table S6. LDA predictions from MA/functional indices sensitivity analysis. Table S7. Comparison of MWAM strain from FEA sensitivity analysis. [file 12915_2022_1294_MOESM1_ESM.docx]

Additional File 1:

Supplemental Figures and Tables

This document accompanies the BMC Biology paper “Diet of exemplary Mesozoic toothed birds (Longipterygidae) from quantitative analysis of extant avian diet proxies” by Miller, Pittman, Wang, Zheng, and Bright. It contains 10 supplementary figures and 7 supplementary tables.

# Supplemental Figures


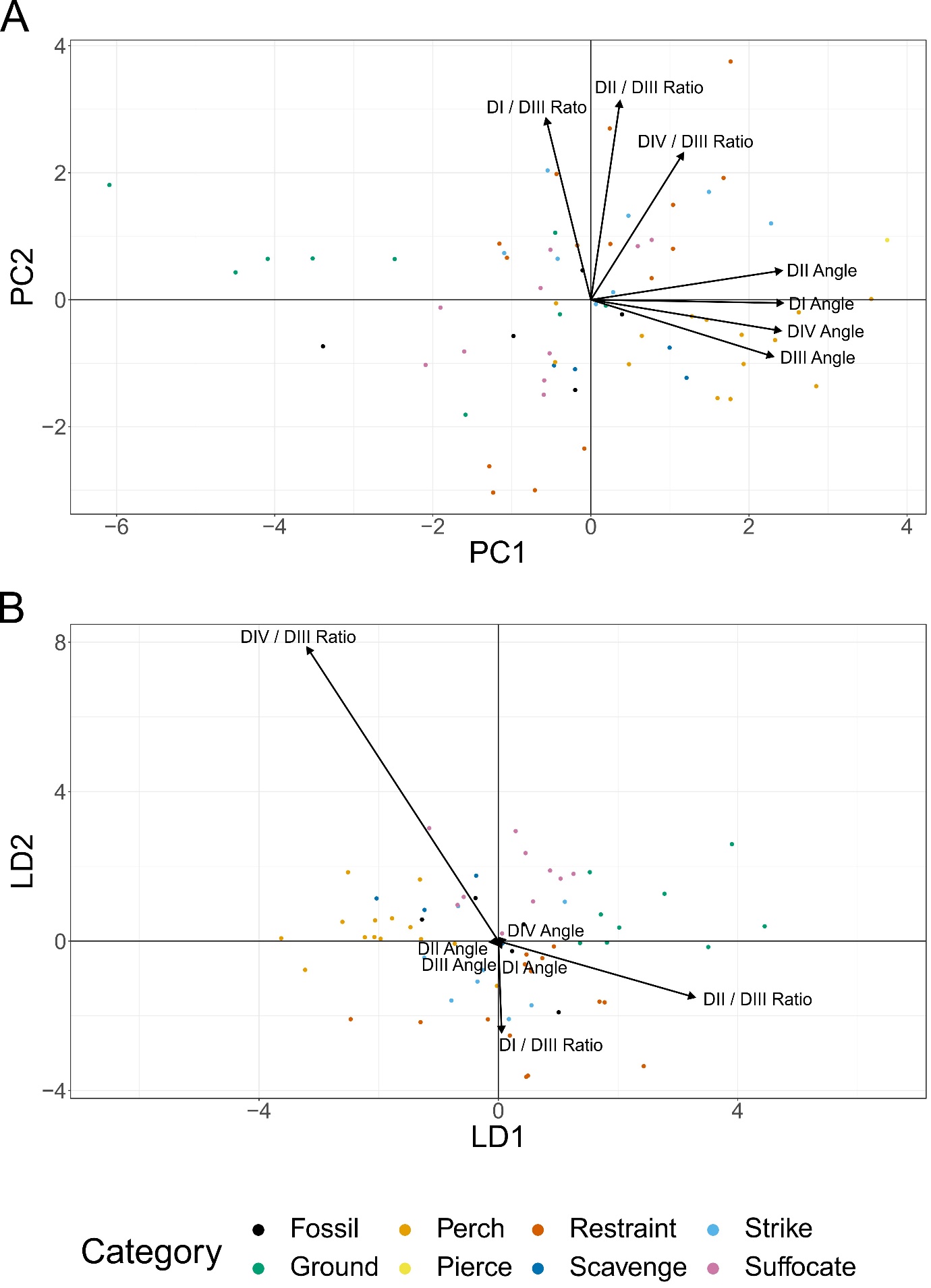


## Figure S1

Plot of character weightings for the graphs in Figure 5 and Figure S7. Plots are provided for PCA (A) and LDA (B).


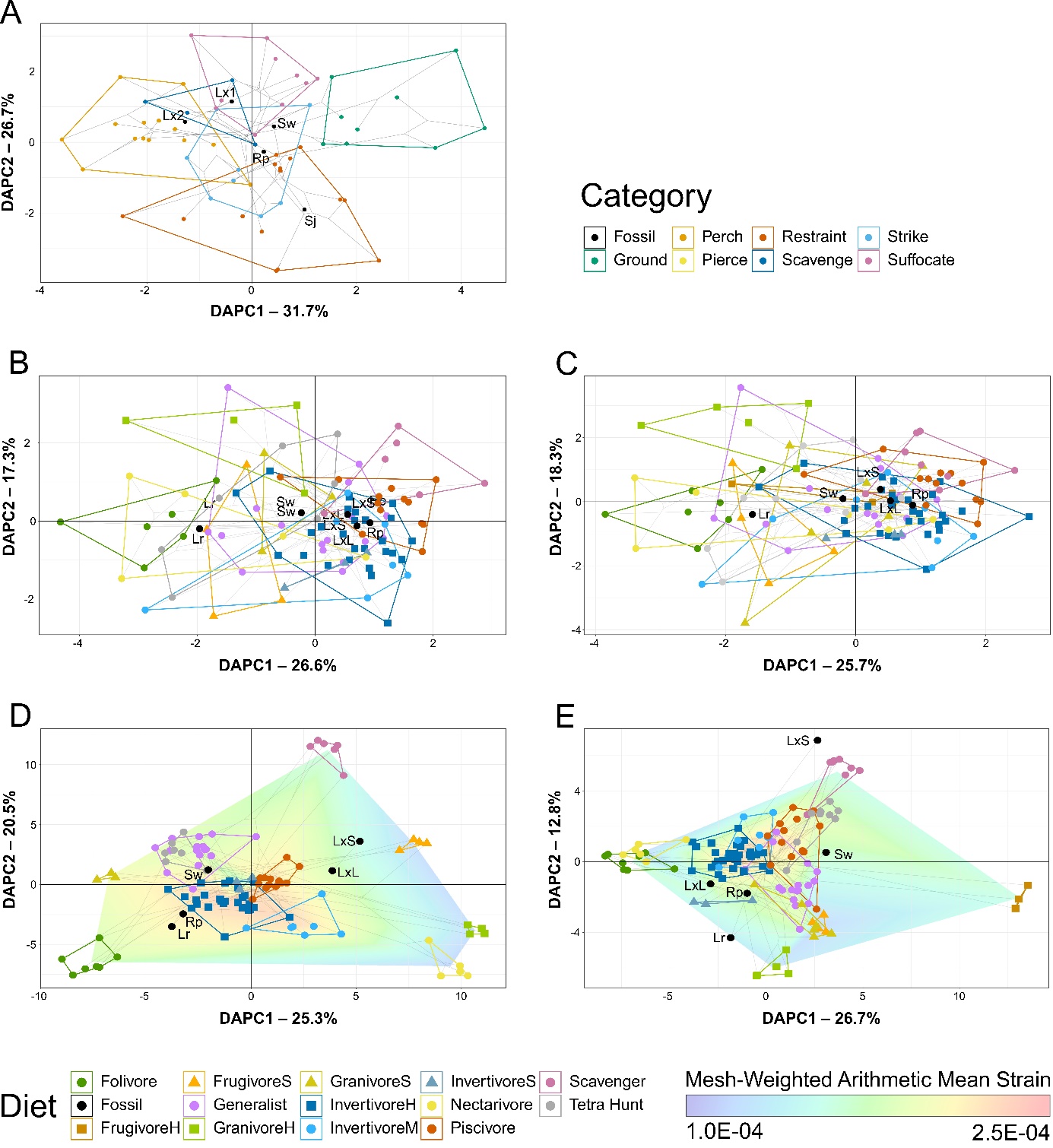


## Figure S2

Discriminant analysis of principal components (DAPC) plots of data analysed in this study. Grey lines indicate phylogenetic relationships. Results are given for pedal traditional morphometrics (A; compare to Figure 5B), upper jaw mechanical advantage and functional indices with semi-specialists excluded (B; compare to Figure 6C) and included (C; compare to Figure 6D), and lower jaw finite element analysis with semi-specialists excluded (D; compare to Figure 8C) and included (E; compare to Figure 8D). Each is identical to its corresponding LDA graph, meaning LDA work in this study is robust to the assumption that input variables are uncorrelated (see Methods). Diet abbreviations: FrugivoreH hard frugivore, FrugivoreS soft frugivore, GranivoreS swallowing granivore, GranivoreH husking granivore, InvertivoreH hard invertivore, InvertivoreM medium invertivore, InvertivoreS soft invertivore, Tetra Hunt tetrapod hunter. Taxon abbreviations: Lr *Longirostravis*, Lx1 *Longipteryx* chaoyangensis, Lx2 *Longipteryx* sp., LxL large-toothed *Longipteryx*, LxS small-toothed *Longipteryx*, Rp *Rapaxavis*, Sw *Shanweiniao*, Sj *Shenjingornis*.


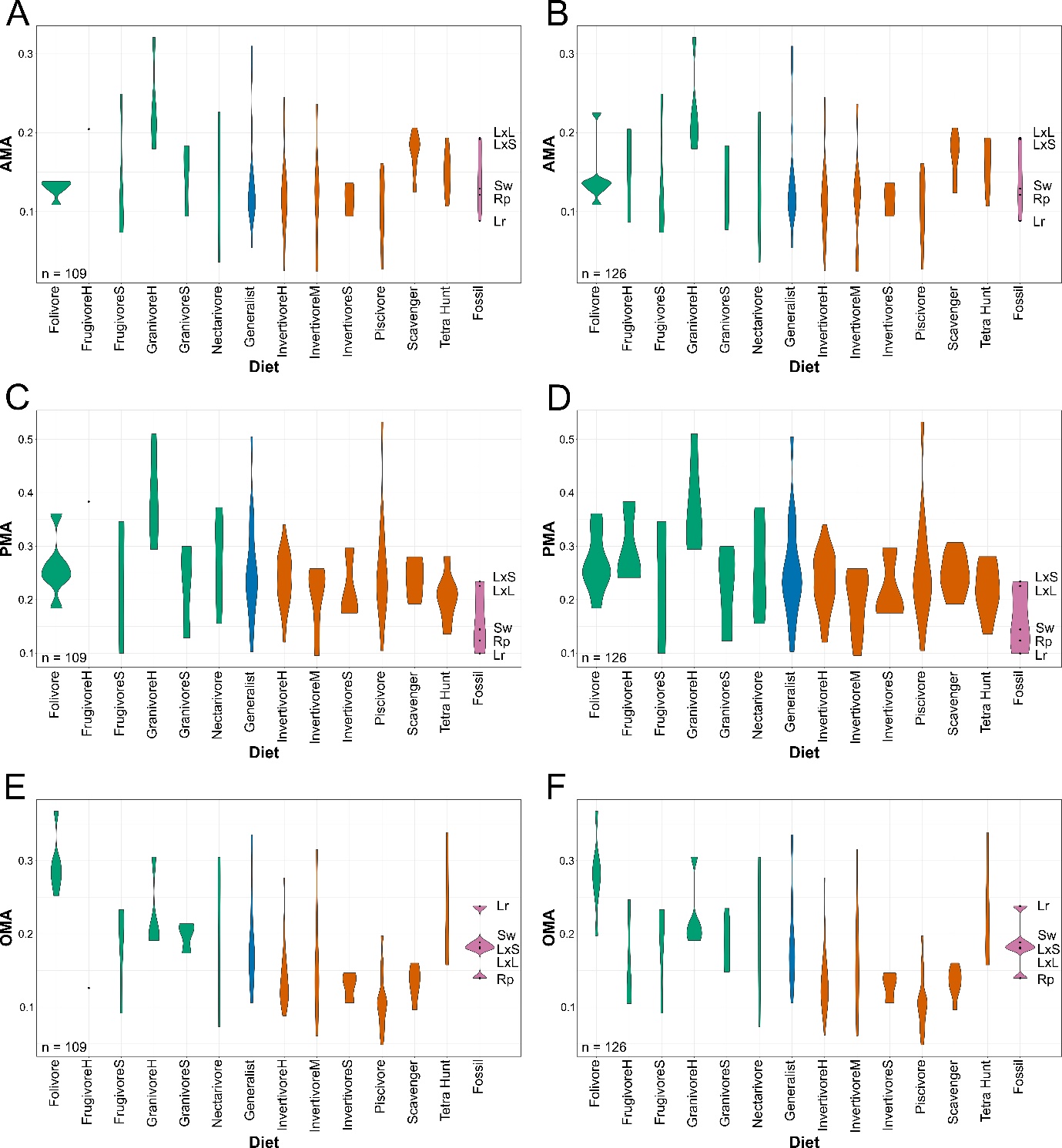


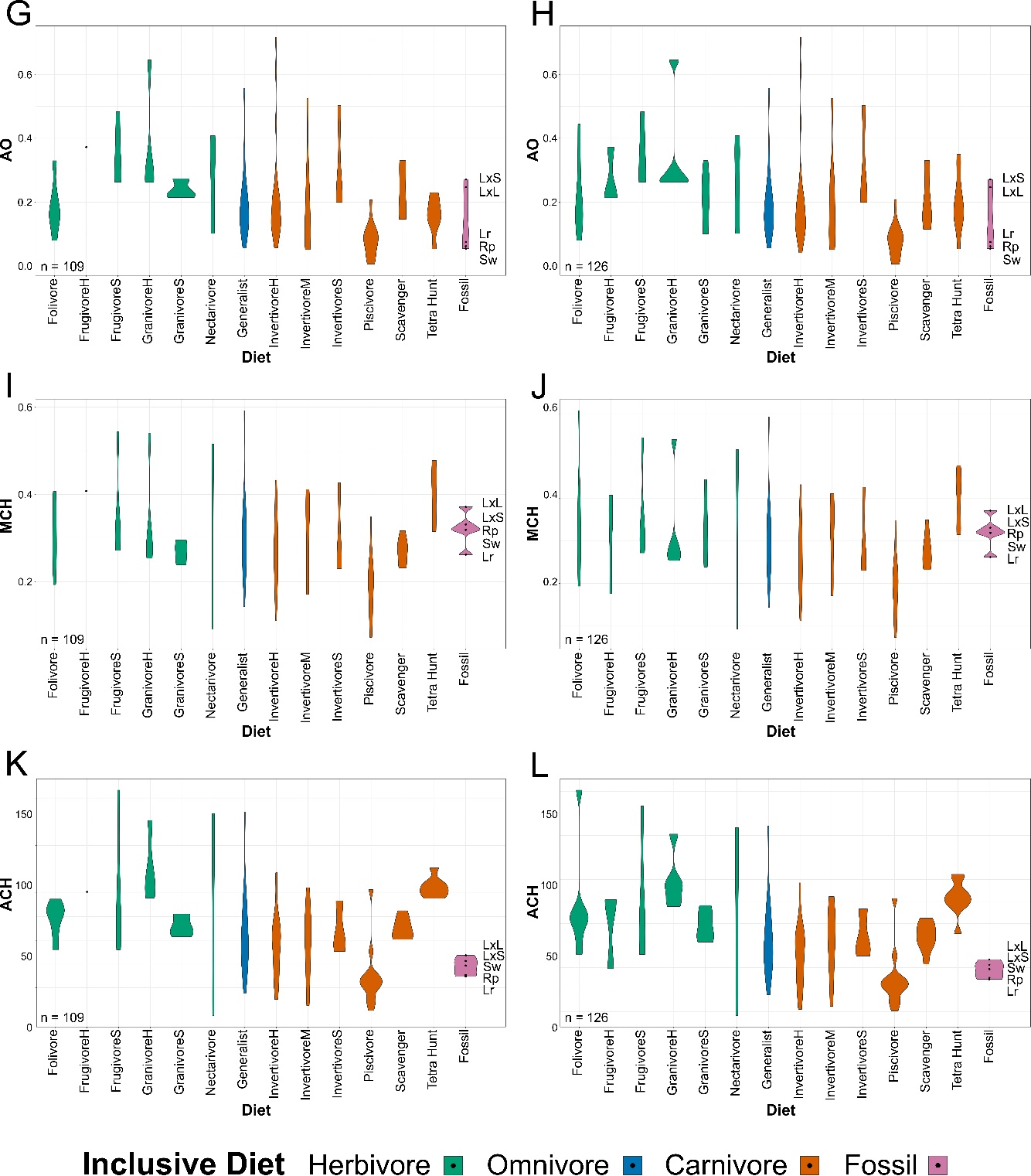


## Figure S3

Violin plots of individual functional indices used in this study. Indices include AMA (A-B), PMA (C-D), OMA (E-F), AO (G-H), MCH (I-J), and ACH (K-L) with data either excluding (A,C,E,G,I,K) or including (B,D,F,H,J,L) semi-specialists. See Methods section of the main paper for explanation of abbreviations. Diet abbreviations: FrugivoreH hard frugivore, FrugivoreS soft frugivore, GranivoreS swallowing granivore, GranivoreH husking granivore, InvertivoreH hard invertivore, InvertivoreM medium invertivore, InvertivoreS soft invertivore, Tetra Hunt tetrapod hunter. Taxon abbreviations: Lr *Longirostravis*, LxL large-toothed *Longipteryx*, LxS small-toothed *Longipteryx*, Rp *Rapaxavis*, Sw *Shanweiniao*.


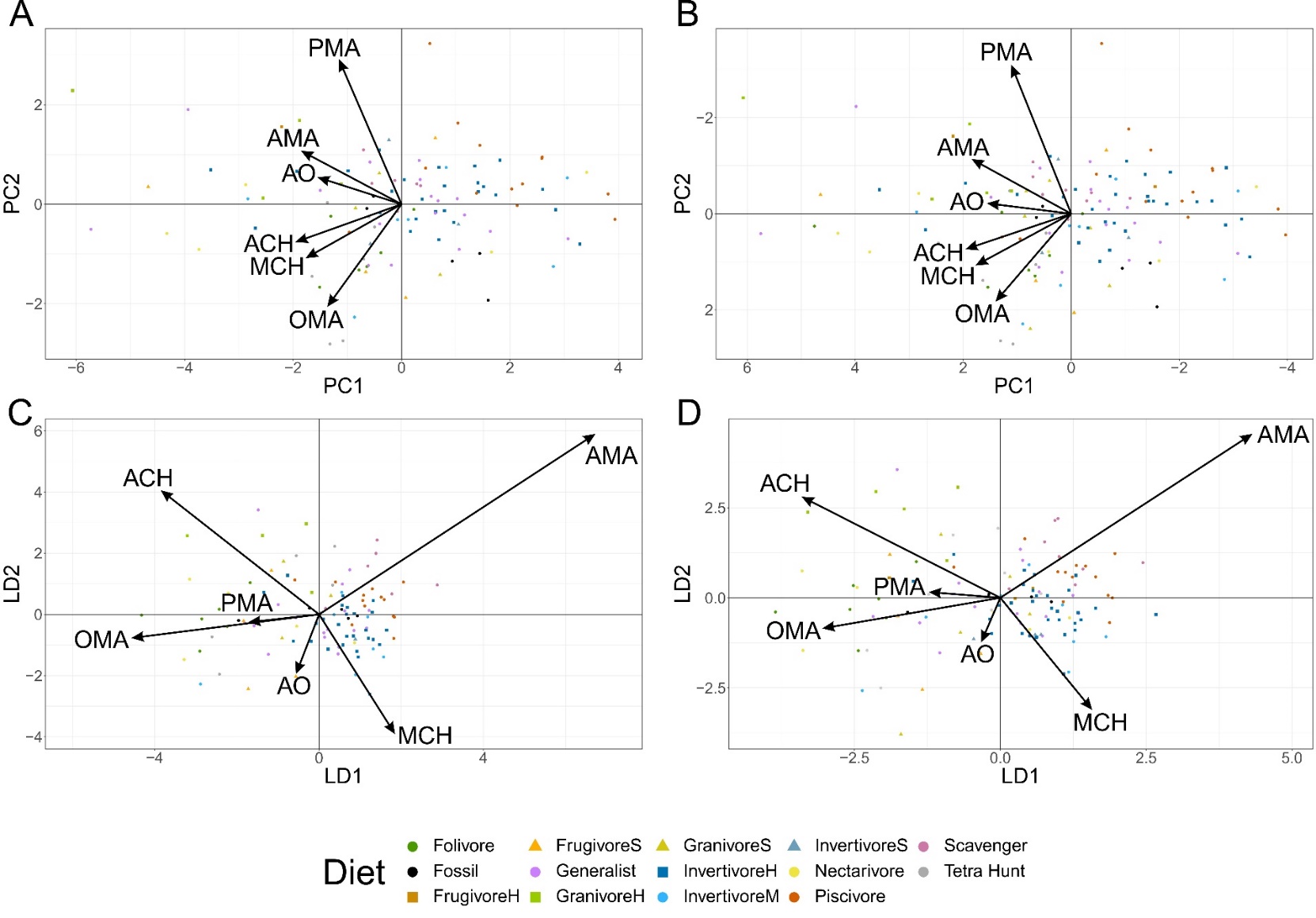


## Figure S4

Plot of character weightings for the graphs in Figure 6. Plots are provided for PCA (A-B) excluding (A) and including (B) semi-specialists, and LDA (C-D) excluding (C) and including (D) semi-specialists.


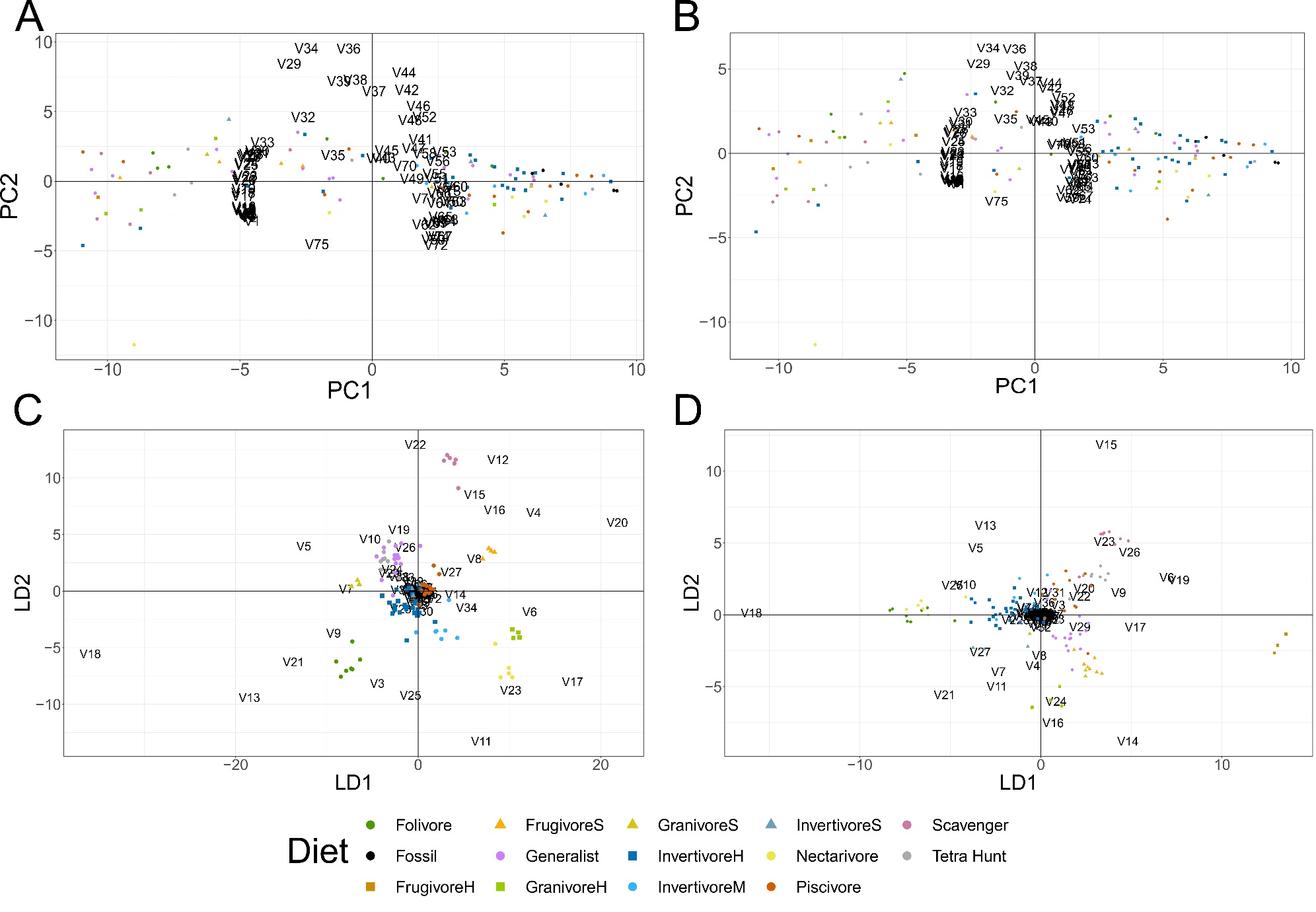


## Figure S5

Plot of character weightings for the graphs in Figure 8. Plots are provided for PCA (A-B) excluding (A) and including (B) semi-specialists, and LDA (C-D) excluding (C) and including (D) semi-specialists. V1 is the interval of lowest strain in all graphs. Note that Figure 8 uses an isometric log-ratio transformation, this plot uses a more easily-interpreted centred log-ratio transformation of the intervals data, so point positions will differ slightly. See the Methods section of the main paper for additional details.


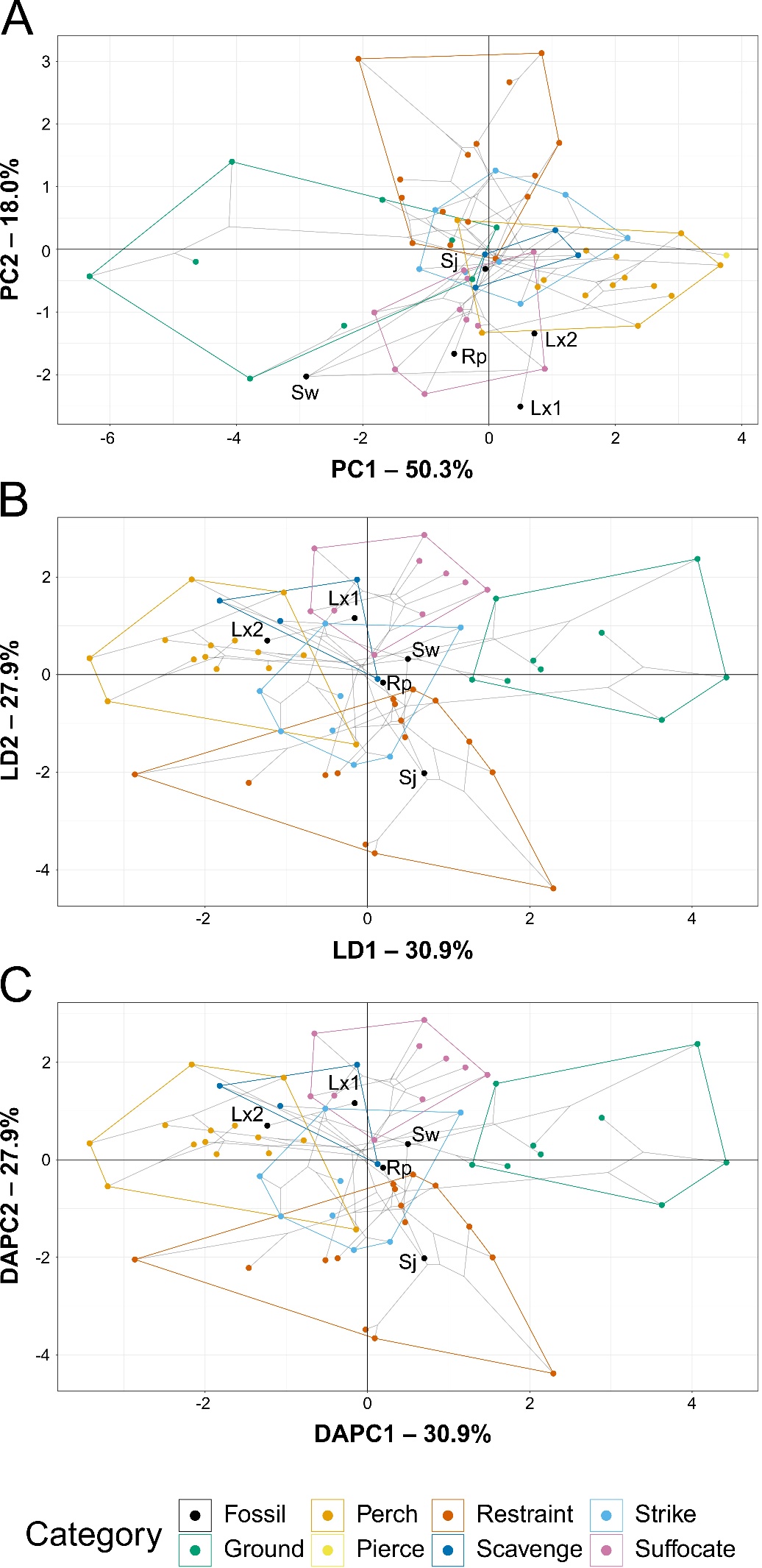


## Figure S6

Phylomorphospace of avian and longipterygid unguals, based on traditional morphometrics, grouped by ecological category. Grey lines indicate phylogenetic relationships. These graphs differ from Figure 5 because their claw size ratios use digit IV as a reference digit rather than digit III. Data is visualised with PCA (A), LDA (B), and DAPC (C). In PCA (A), PC1 describes talon curvature and PC2 describes interdigital size variation. In LDA (B), LD1 describes the size ratio of digit II to digit IV and LD2 describes the size ratio of digits I and III to digit IV. Taxon abbreviations: Lx1 *Longipteryx chaoyangensis*, Lx2 *Longipteryx* sp., Rp *Rapaxavis pani*, Sw *Shanweiniao cooperorum*, Sj *Shenjingornis yangi*.


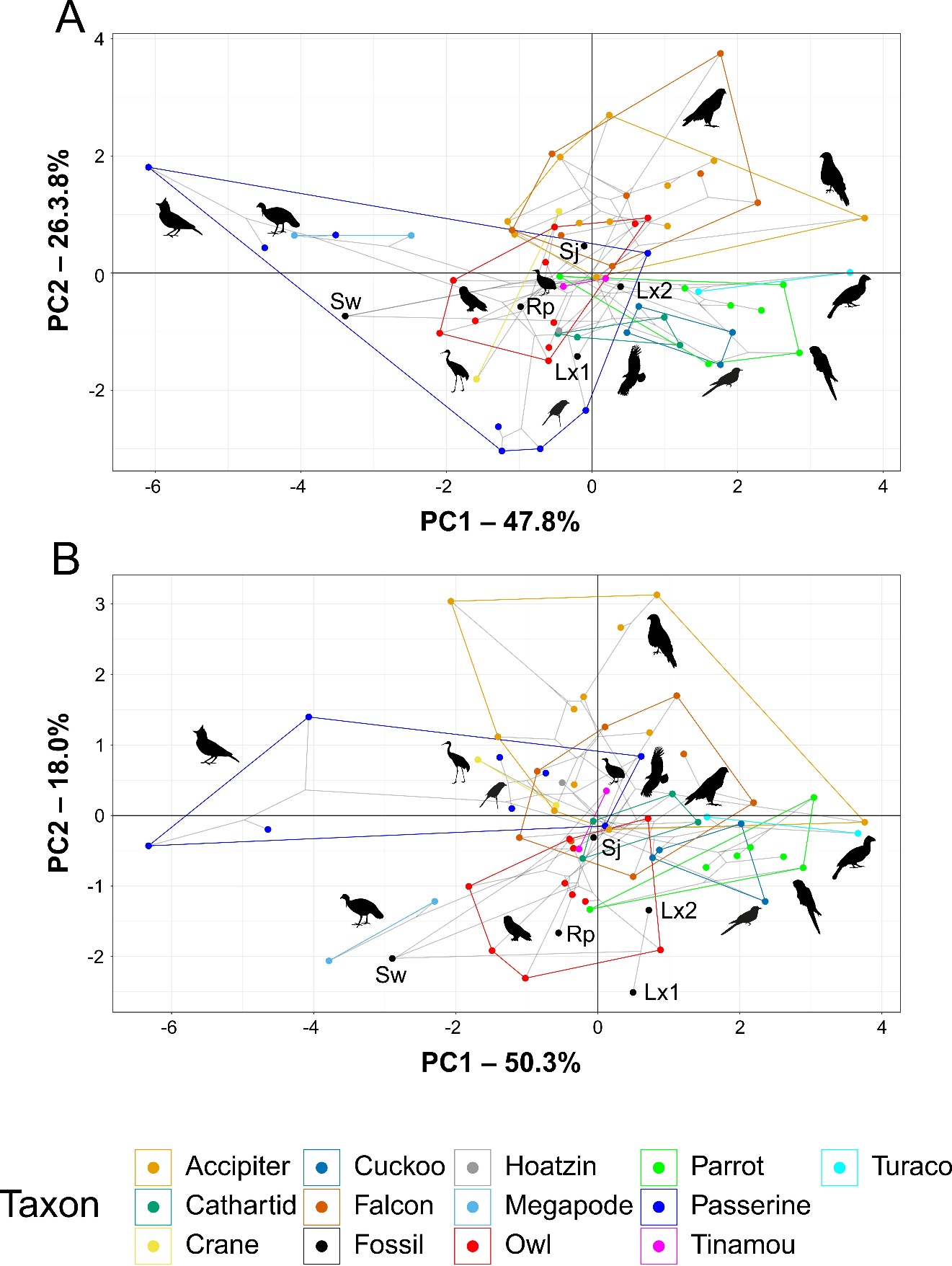


## Figure S7

Phylomorphospace of avian and longipterygid unguals, based on traditional morphometrics, grouped by broad phylogenetic clade. Grey lines indicate phylogenetic relationships. Data is visualised with PCA using digit III as a reference digit (A) and digit IV as a reference digit (B). In both PC1 describes talon curvature and PC2 describes interdigital size variation. See Figure S1 for precise character loadings. Images for each clade are public domain from phylopic.org. Taxon abbreviations: Lx1 *Longipteryx chaoyangensis*, Lx2 *Longipteryx* sp., Rp *Rapaxavis pani*, Sw *Shanweiniao cooperorum*, Sj *Shenjingornis yangi*.


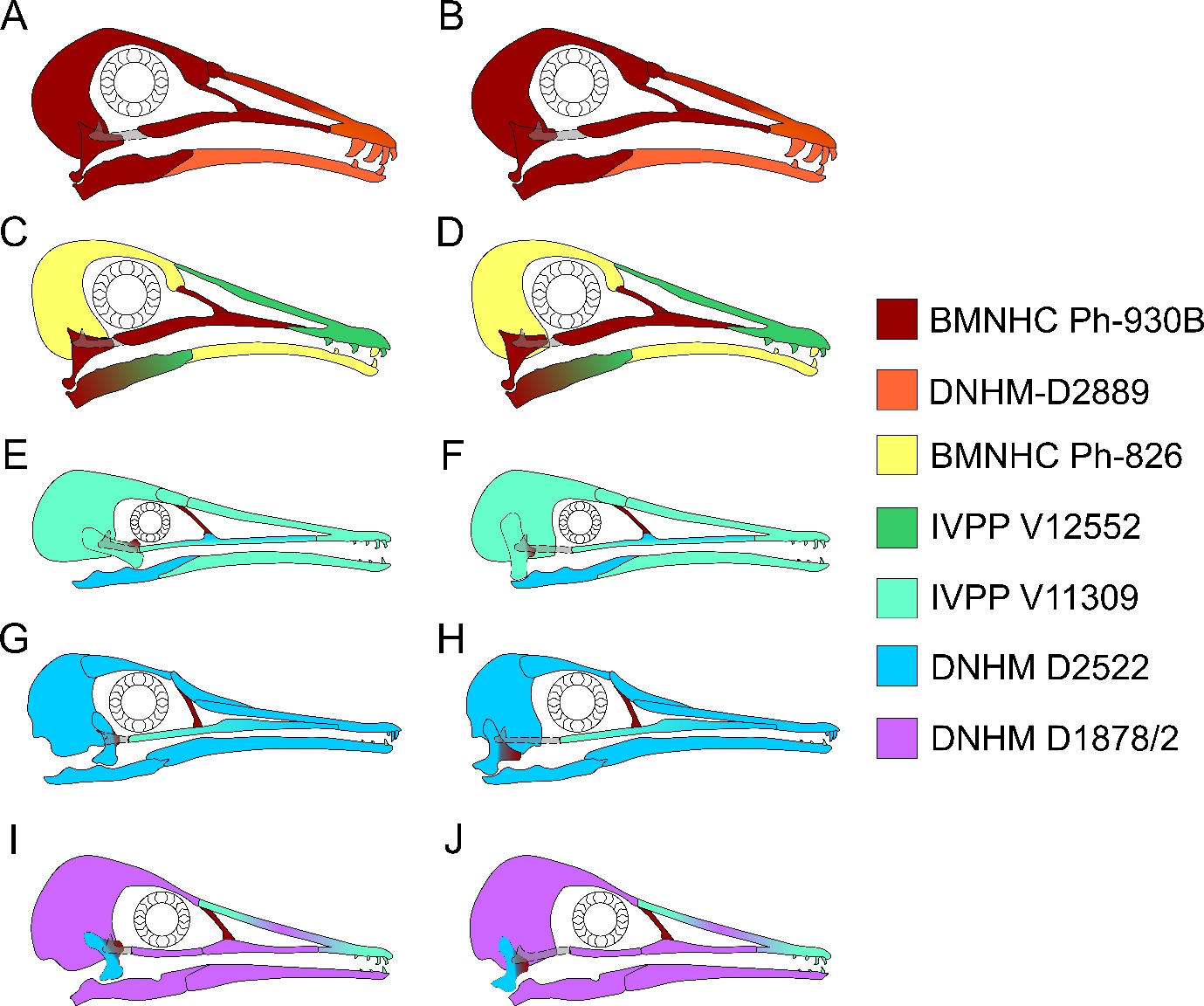


## Figure S8

Reconstructions of longipterygid skulls for jaw joint sensitivity analysis. Quadrates are shifted as far anteriorly (A, C, E, G, I) or posteriorly (B, D, F, H, J) as biologically possible to test the effect of their position on MA and functional index results. The quadrate appears *in situ* in BMNHC Ph-930B, so quadrate shifting in *Longipteryx* (A-D) is more limited. Reconstructions are of *Longipteryx* morphotypes with large teeth (A-B) and small teeth (C-D), *Longirostravis* (E-F), *Rapaxavis* (G-H), and *Shanweiniao* (I-J). Colours of different bones indicate which specimen that bone is based on. All sclerotic rings are based on BMNHC Ph-930B. See the Methods section and Figure 1 for more details on reconstruction.


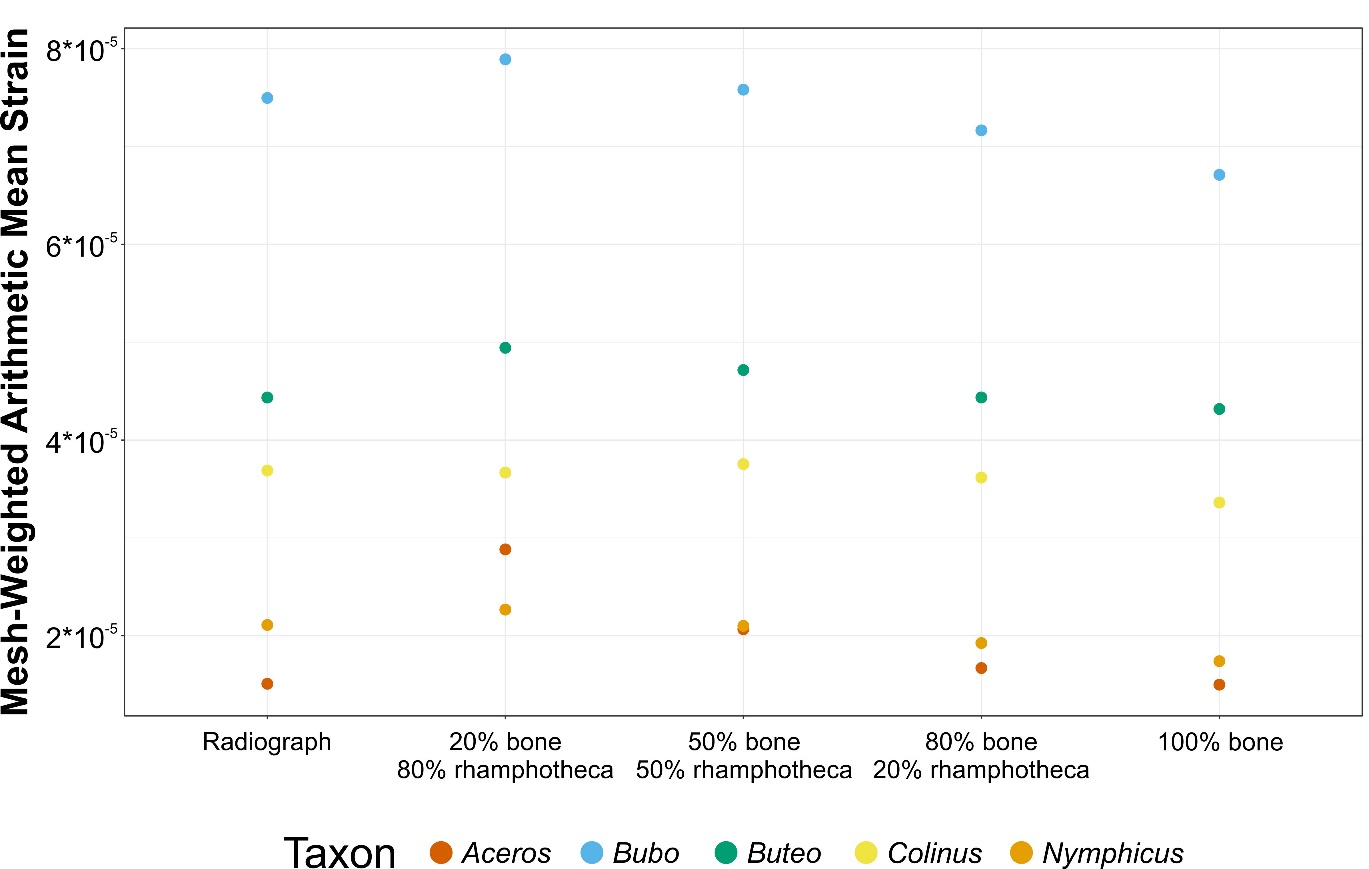


## Figure S9

MWAM strain results of rhamphotheca thickness sensitivity analysis. Models were created based on the true thickness of rhamphotheca as seen in radiographs of bird skulls and compared to models with varying dorsoventral thickness of bone and rhamphotheca (with bone in the centre and rhamphotheca thickness evenly split between the dorsal and ventral sides). Note that the 80% bone 20% rhamphotheca models, the assumption used for unknown thicknesses in this study, are most similar overall to the radiograph models (quantified in Table S7). *Aceros* model is based on an unpublished radiograph of *Aceros undulatus* provided by Kathryn C. Gamble; see Specimen Selection for publications with radiographs of other taxa.


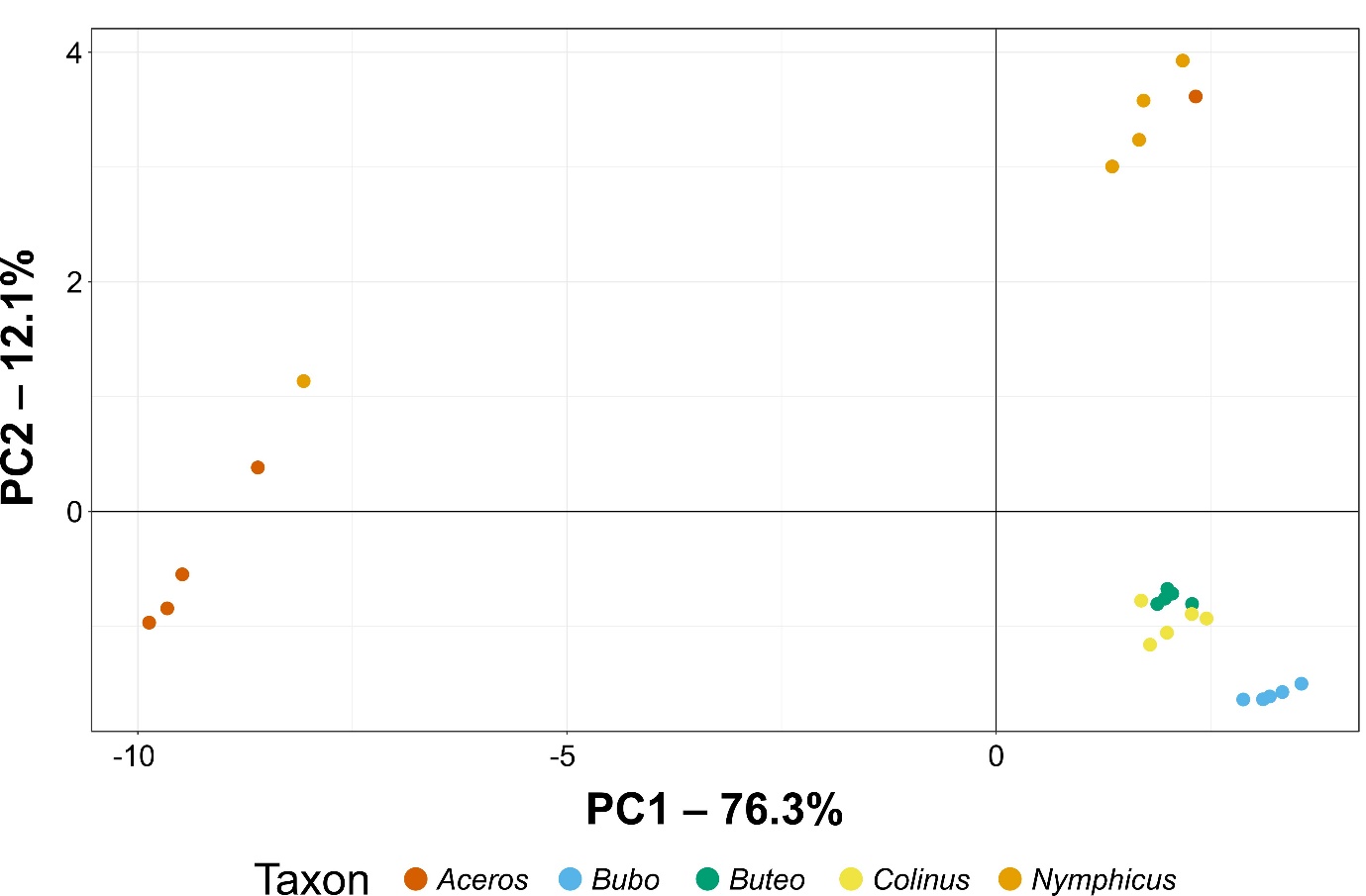


## Figure S10

Strain-space of results of rhamphotheca thickness sensitivity analysis. Overall, models of the same taxon all cluster together, showing similar trends in strain across assumptions of rhamphotheca thickness. The exceptions are the 20% bone 80% rhamphotheca model of *Aceros* and the 100% bone model of *Nymphicus*, which cluster alongside models of the other bird. This is not necessarily surprising, as these are the models most different from the true rhamphotheca thickness (*Aceros* has a very thin rhamphotheca, *Nymphicus* has a very thick rhamphotheca) and these models have MWAM strains more similar to those they cluster with (Figure S9). In all cases the thickness assumption used in this study, 80% bone 20% rhamphotheca, clustered with the radiograph-based model. *Aceros* model is based on an unpublished radiograph of *Aceros undulatus* provided by Kathryn C. Gamble; see Specimen Selection for publications with radiographs of other taxa.

# Supplemental Tables

## Table S1

p-values for phylogenetic HSD testing whether unguals of extant birds with different ecological groups have different shape via traditional morphometrics. Piercing raptors are excluded as only a single taxon represents this group. p-values are indicated with one asterisk (*) for significance at the 0.05 level, two at the 0.01 level, and three at the 0.001 level. Note that the pairwise() function in RRPP [42] places a lower limit on the returned p-value, so p-values reported as 1.00E-03 may be more significant.

|  | **Ground** | **Perch** | **Restraint** | **Scavenge** | **Strike** | **Suffocate** |
| --- | --- | --- | --- | --- | --- | --- |
| **Ground** |  | 1.00E-03*** | 6.00E-03** | 1.60E-01 | 9.60E-02 | 5.45E-01 |
| **Perch** | 1.00E-03*** |  | 9.07E-01 | 7.09E-01 | 2.97E-01 | 1.65E-01 |
| **Restraint** | 6.00E-03** | 9.07E-01 |  | 8.39E-01 | 5.96E-01 | 2.98E-01 |
| **Scavenge** | 1.60E-01 | 7.09E-01 | 8.39E-01 |  | 7.56E-01 | 5.93E-01 |
| **Strike** | 9.60E-02 | 2.97E-01 | 5.96E-01 | 7.56E-01 |  | 6.92E-01 |
| **Suffocate** | 5.45E-01 | 1.65E-01 | 2.98E-01 | 5.93E-01 | 6.92E-01 |  |

## Table S2

K statistics [44] for individual variables used in TM analyses. K = 1 indicates a similarity of measured traits expected if traits evolved under Brownian motion, values less than 1 indicate traits more different than expected from Brownian motion and values greater than 1 indicate traits more similar than expected from Brownian motion. p-values are indicated with one asterisk (*) for significance at the 0.05 level, two at the 0.01 level, and three at the 0.001 level. Significant p-values indicate the presence of phylogenetic signal. Note that the K_mult_ function in [40] places a lower limit on the returned p-value, so p-values reported as 1.00E-03 may be more significant.

|  | **K** | **p-value** |
| --- | --- | --- |
| **DI/DIII Ratio** | 0.843968 | 1.00E-03*** |
| **DII/DIII Ratio** | 0.72738 | 1.00E-03*** |
| **DIV/DIII Ratio** | 0.537743 | 1.00E-03*** |
| **DI Angle** | 0.833498 | 1.00E-03*** |
| **DII Angle** | 0.926368 | 1.00E-03*** |
| **DIII Angle** | 0.553193 | 1.00E-03*** |
| **DIV Angle** | 0.424729 | 8.00E-03** |

## Table S3

p-values for phylogenetic HSD testing whether skulls of extant birds with different diets are mechanically different via mechanical advantage and functional indices. Values are given with semi-specialists excluded (A) and included (B). FrugivoreH is excluded from A as only a single taxon represents this group. p-values are indicated with one asterisk (*) for significance at the 0.05 level, two at the 0.01 level, and three at the 0.001 level. Note that the pairwise() function in RRPP [42] places a lower limit on the returned p-value, so p-values reported as 1.00E-03 may be more significant.

| **A** | **Foli-vore** | **Frugi-voreS** | **General-ist** | **Grani-voreH** | **Grani-voreS** | **Inverti-voreH** | **Inverti-voreM** | **Inverti-voreS** | **Nectari-vore** | **Piscivore** | **Scavenger** | **Tetra Hunt** |
| --- | --- | --- | --- | --- | --- | --- | --- | --- | --- | --- | --- | --- |
| **Folivore** |  | 1.12E-01 | 1.53E-01 | 1.76E-01 | 3.72E-01 | 9.00E-03** | 7.60E-02 | 7.10E-02 | 2.10E-02* | 2.00E-03** | 7.70E-02 | 1.69E-01 |
| **FrugivoreS** | 1.12E-01 |  | 4.13E-01 | 9.04E-01 | 6.51E-01 | 3.73E-01 | 4.99E-01 | 7.87E-01 | 1.40E-02* | 2.00E-03** | 2.40E-01 | 4.68E-01 |
| **Generalist** | 1.53E-01 | 4.13E-01 |  | 4.25E-01 | 9.58E-01 | 9.30E-02 | 6.75E-01 | 4.61E-01 | 4.40E-02* | 3.00E-03** | 2.93E-01 | 7.46E-01 |
| **GranivoreH** | 1.76E-01 | 9.04E-01 | 4.25E-01 |  | 8.53E-01 | 2.52E-01 | 3.19E-01 | 6.87E-01 | 2.40E-02* | 3.00E-03** | 3.82E-01 | 4.87E-01 |
| **GranivoreS** | 3.72E-01 | 6.51E-01 | 9.58E-01 | 8.53E-01 |  | 9.21E-01 | 8.70E-01 | 8.57E-01 | 1.78E-01 | 1.47E-01 | 7.63E-01 | 9.74E-01 |
| **InvertivoreH** | 9.00E-03** | 3.73E-01 | 9.30E-02 | 2.52E-01 | 9.21E-01 |  | 7.60E-01 | 8.49E-01 | 5.70E-02 | 1.00E-03*** | 6.06E-01 | 7.38E-01 |
| **InvertivoreM** | 7.60E-02 | 4.99E-01 | 6.75E-01 | 3.19E-01 | 8.70E-01 | 7.60E-01 |  | 6.94E-01 | 1.25E-01 | 6.00E-03** | 3.38E-01 | 9.37E-01 |
| **InvertivoreS** | 7.10E-02 | 7.87E-01 | 4.61E-01 | 6.87E-01 | 8.57E-01 | 8.49E-01 | 6.94E-01 |  | 6.70E-02 | 4.80E-02* | 6.14E-01 | 3.22E-01 |
| **Nectarivore** | 2.10E-02* | 1.40E-02* | 4.40E-02* | 2.40E-02* | 1.78E-01 | 5.70E-02 | 1.25E-01 | 6.70E-02 |  | 3.97E-01 | 1.61E-01 | 2.39E-01 |
| **Piscivore** | 2.00E-03** | 2.00E-03** | 3.00E-03** | 3.00E-03** | 1.47E-01 | 1.00E-03*** | 6.00E-03** | 4.80E-02* | 3.97E-01 |  | 2.34E-01 | 5.90E-02 |
| **Scavenger** | 7.70E-02 | 2.40E-01 | 2.93E-01 | 3.82E-01 | 7.63E-01 | 6.06E-01 | 3.38E-01 | 6.14E-01 | 1.61E-01 | 2.34E-01 |  | 3.42E-01 |
| **Tetra Hunt** | 1.69E-01 | 4.68E-01 | 7.46E-01 | 4.87E-01 | 9.74E-01 | 7.38E-01 | 9.37E-01 | 3.22E-01 | 2.39E-01 | 5.90E-02 | 3.42E-01 |  |

| **B** | **Foli-vore** | **Frugi-voreH** | **Frugi-voreS** | **General-ist** | **Grani-voreH** | **Grani-voreS** | **Inverti-voreH** | **Inverti-voreM** | **Inverti-voreS** | **Nectari-vore** | **Piscivore** | **Scavenger** | **Tetra Hunt** |
| --- | --- | --- | --- | --- | --- | --- | --- | --- | --- | --- | --- | --- | --- |
| **Folivore** |  | 6.02E-01 | 1.84E-01 | 3.22E-01 | 2.53E-01 | 2.06E-01 | 8.00E-03** | 1.44E-01 | 1.47E-01 | 3.44E-01 | 2.00E-03** | 8.00E-02 | 4.46E-01 |
| **FrugivoreH** | 6.02E-01 |  | 7.27E-01 | 7.03E-01 | 9.69E-01 | 8.56E-01 | 6.53E-01 | 5.42E-01 | 8.61E-01 | 3.46E-01 | 1.79E-01 | 5.21E-01 | 7.44E-01 |
| **FrugivoreS** | 1.84E-01 | 7.27E-01 |  | 2.42E-01 | 7.21E-01 | 6.09E-01 | 2.21E-01 | 4.53E-01 | 7.95E-01 | 1.24E-01 | 3.00E-03** | 5.70E-02 | 5.49E-01 |
| **Generalist** | 3.22E-01 | 7.03E-01 | 2.42E-01 |  | 1.32E-01 | 7.48E-01 | 2.00E-03** | 5.20E-01 | 3.85E-01 | 6.22E-01 | 2.00E-03** | 1.06E-01 | 9.66E-01 |
| **GranivoreH** | 2.53E-01 | 9.69E-01 | 7.21E-01 | 1.32E-01 |  | 5.10E-01 | 3.80E-02* | 1.08E-01 | 5.22E-01 | 9.60E-02 | 1.00E-03*** | 3.50E-02* | 3.65E-01 |
| **GranivoreS** | 2.06E-01 | 8.56E-01 | 6.09E-01 | 7.48E-01 | 5.10E-01 |  | 3.06E-01 | 6.74E-01 | 8.19E-01 | 4.44E-01 | 1.70E-02 | 4.23E-01 | 9.57E-01 |
| **InvertivoreH** | 8.00E-03** | 6.53E-01 | 2.21E-01 | 2.00E-03** | 3.80E-02* | 3.06E-01 |  | 5.11E-01 | 7.35E-01 | 2.79E-01 | 2.00E-03** | 1.20E-02* | 4.08E-01 |
| **InvertivoreM** | 1.44E-01 | 5.42E-01 | 4.53E-01 | 5.20E-01 | 1.08E-01 | 6.74E-01 | 5.11E-01 |  | 6.22E-01 | 7.02E-01 | 1.00E-03*** | 1.93E-01 | 9.38E-01 |
| **InvertivoreS** | 1.47E-01 | 8.61E-01 | 7.95E-01 | 3.85E-01 | 5.22E-01 | 8.19E-01 | 7.35E-01 | 6.22E-01 |  | 2.72E-01 | 6.50E-02 | 2.62E-01 | 4.13E-01 |
| **Nectarivore** | 3.44E-01 | 3.46E-01 | 1.24E-01 | 6.22E-01 | 9.60E-02 | 4.44E-01 | 2.79E-01 | 7.02E-01 | 2.72E-01 |  | 2.70E-01 | 6.87E-01 | 7.75E-01 |
| **Piscivore** | 2.00E-03** | 1.79E-01 | 3.00E-03** | 2.00E-03** | 1.00E-03*** | 1.70E-02 | 2.00E-03 | 1.00E-03*** | 6.50E-02 | 2.70E-01 |  | 2.48E-01 | 3.00E-02* |
| **Scavenger** | 8.00E-02 | 5.21E-01 | 5.70E-02 | 1.06E-01 | 3.50E-02* | 4.23E-01 | 1.20E-02* | 1.93E-01 | 2.62E-01 | 6.87E-01 | 2.48E-01 |  | 4.08E-01 |
| **Tetra Hunt** | 4.46E-01 | 7.44E-01 | 5.49E-01 | 9.66E-01 | 3.65E-01 | 9.57E-01 | 4.08E-01 | 9.38E-01 | 4.13E-01 | 7.75E-01 | 3.00E-02* | 4.08E-01 |  |

## Table S4

K statistics [44] for individual variables used in MA and functional index analyses. K = 1 indicates a similarity of measured traits expected if traits evolved under Brownian motion, values less than 1 indicate traits more different than expected from Brownian motion and values greater than 1 indicate traits more similar than expected from Brownian motion. p-values are indicated with one asterisk (*) for significance at the 0.05 level, two at the 0.01 level, and three at the 0.001 level. Significant p-values indicate the presence of phylogenetic signal. Note that the K_mult_ function in [40] places a lower limit on the returned p-value, so p-values reported as 1.00E-03 may be more significant.

|  | **K**  **No Semi-specialists** | **p-value**  **No Semi-specialists** | **K**  **All Birds** | **p-value**  **All Birds** |
| --- | --- | --- | --- | --- |
| **AMA** | 0.7841247 | 2.00E-03** | 0.7473438 | 1.00E-03*** |
| **PMA** | 0.6046434 | 1.70E-02* | 0.5364633 | 5.00E-03** |
| **OMA** | 0.4908092 | 1.09E-01 | 0.4282945 | 7.30E-02 |
| **AO** | 0.9649066 | 1.00E-03*** | 0.7869946 | 1.00E-03*** |
| **MCH** | 1.0694944 | 1.00E-03*** | 1.0624462 | 1.00E-03*** |
| **ACH** | 1.3433595 | 1.00E-03*** | 1.3315524 | 1.00E-03*** |

## Table S5

p-values for phylogenetic HSD testing whether skulls of extant birds with different diets are mechanically different via comparing strain intervals after finite element analysis. Values are given with semi-specialists excluded (A) and included (B). FrugivoreH is excluded from A as only a single taxon represents this group. p-values are indicated with one asterisk (*) for significance at the 0.05 level, two at the 0.01 level, and three at the 0.001 level. Note that the pairwise() function in RRPP [42] places a lower limit on the returned p-value, so p-values reported as 1.00E-3 may be more significant.

| **A** | **Foli-vore** | **Frugi-voreS** | **General-ist** | **Grani-voreH** | **Grani-voreS** | **Inverti-voreH** | **Inverti-voreM** | **Inverti-voreS** | **Nectari-vore** | **Pisci-vore** | **Scavenger** | **Tetra Hunt** |
| --- | --- | --- | --- | --- | --- | --- | --- | --- | --- | --- | --- | --- |
| **Folivore** |  | 8.68E-01 | 2.37E-01 | 1.14E-01 | 2.40E-01 | 6.20E-02 | 2.60E-02* | 8.40E-02 | 2.67E-01 | 2.70E-02* | 2.78E-01 | 3.80E-02* |
| **FrugivoreS** | 8.68E-01 |  | 6.39E-01 | 2.49E-01 | 4.03E-01 | 1.84E-01 | 7.10E-02 | 1.00E-01 | 1.90E-01 | 1.68E-01 | 5.27E-01 | 9.30E-02 |
| **Generalist** | 2.37E-01 | 6.39E-01 |  | 8.60E-02 | 4.32E-01 | 7.00E-03** | 2.50E-02* | 5.90E-02 | 4.20E-01 | 3.80E-02* | 4.57E-01 | 5.60E-02 |
| **GranivoreH** | 1.14E-01 | 2.49E-01 | 8.60E-02 |  | 2.15E-01 | 3.45E-01 | 1.35E-01 | 5.90E-02 | 4.24E-01 | 2.15E-01 | 5.41E-01 | 9.30E-02 |
| **GranivoreS** | 2.40E-01 | 4.03E-01 | 4.32E-01 | 2.15E-01 |  | 4.83E-01 | 3.58E-01 | 3.04E-01 | 5.14E-01 | 6.22E-01 | 5.82E-01 | 3.93E-01 |
| **InvertivoreH** | 6.20E-02 | 1.84E-01 | 7.00E-03** | 3.45E-01 | 4.83E-01 |  | 3.91E-01 | 1.23E-01 | 6.36E-01 | 3.88E-01 | 5.39E-01 | 8.60E-02 |
| **InvertivoreM** | 2.60E-02* | 7.10E-02 | 2.50E-02* | 1.35E-01 | 3.58E-01 | 3.91E-01 |  | 6.76E-01 | 8.97E-01 | 3.39E-01 | 2.31E-01 | 2.77E-01 |
| **InvertivoreS** | 8.40E-02 | 1.00E-01 | 5.90E-02 | 5.90E-02 | 3.04E-01 | 1.23E-01 | 6.76E-01 |  | 7.47E-01 | 3.55E-01 | 1.05E-01 | 2.91E-01 |
| **Nectarivore** | 2.67E-01 | 1.90E-01 | 4.20E-01 | 4.24E-01 | 5.14E-01 | 6.36E-01 | 8.97E-01 | 7.47E-01 |  | 7.90E-01 | 5.27E-01 | 7.70E-01 |
| **Piscivore** | 2.70E-02* | 1.68E-01 | 3.80E-02* | 2.15E-01 | 6.22E-01 | 3.88E-01 | 3.39E-01 | 3.55E-01 | 7.90E-01 |  | 4.70E-01 | 2.98E-01 |
| **Scavenger** | 2.78E-01 | 5.27E-01 | 4.57E-01 | 5.41E-01 | 5.82E-01 | 5.39E-01 | 2.31E-01 | 1.05E-01 | 5.27E-01 | 4.70E-01 |  | 4.32E-01 |
| **Tetra Hunt** | 3.80E-02* | 9.30E-02 | 5.60E-02 | 9.30E-02 | 3.93E-01 | 8.60E-02 | 2.77E-01 | 2.91E-01 | 7.70E-01 | 2.98E-01 | 4.32E-01 |  |

| **B** | **Foli-vore** | **Frugi-voreH** | **Frugi-voreS** | **General-ist** | **Grani-voreH** | **Grani-voreS** | **Inverti-voreH** | **Inverti-voreM** | **Inverti-voreS** | **Nectari-vore** | **Pisci-vore** | **Scavenger** | **Tetra Hunt** |
| --- | --- | --- | --- | --- | --- | --- | --- | --- | --- | --- | --- | --- | --- |
| **Folivore** |  | 4.00E-01 | 8.68E-01 | 2.16E-01 | 1.86E-01 | 1.51E-01 | 1.50E-02* | 1.60E-02* | 1.46E-01 | 4.95E-01 | 2.10E-02* | 1.90E-02* | 1.45E-01 |
| **FrugivoreH** | 4.00E-01 |  | 7.01E-01 | 3.74E-01 | 6.72E-01 | 3.05E-01 | 1.59E-01 | 1.16E-01 | 1.22E-01 | 3.59E-01 | 1.52E-01 | 4.79E-01 | 2.21E-01 |
| **FrugivoreS** | 8.68E-01 | 7.01E-01 |  | 5.67E-01 | 5.64E-01 | 4.71E-01 | 1.90E-01 | 1.31E-01 | 2.76E-01 | 5.33E-01 | 2.35E-01 | 3.88E-01 | 3.32E-01 |
| **Generalist** | 2.16E-01 | 3.74E-01 | 5.67E-01 |  | 1.18E-01 | 5.42E-01 | 1.00E-03*** | 1.90E-02* | 1.26E-01 | 5.61E-01 | 1.20E-02* | 3.00E-03** | 1.09E-01 |
| **GranivoreH** | 1.86E-01 | 6.72E-01 | 5.64E-01 | 1.18E-01 |  | 3.04E-01 | 2.51E-01 | 1.52E-01 | 1.68E-01 | 7.15E-01 | 2.71E-01 | 1.59E-01 | 3.47E-01 |
| **GranivoreS** | 1.51E-01 | 3.05E-01 | 4.71E-01 | 5.42E-01 | 3.04E-01 |  | 3.72E-01 | 3.89E-01 | 4.40E-01 | 7.88E-01 | 7.70E-01 | 2.00E-02* | 5.12E-01 |
| **InvertivoreH** | 1.50E-02* | 1.59E-01 | 1.90E-01 | 1.00E-03*** | 2.51E-01 | 3.72E-01 |  | 5.21E-01 | 2.26E-01 | 4.90E-01 | 4.86E-01 | 2.00E-03** | 2.97E-01 |
| **InvertivoreM** | 1.60E-02* | 1.16E-01 | 1.31E-01 | 1.90E-02* | 1.52E-01 | 3.89E-01 | 5.21E-01 |  | 7.44E-01 | 5.75E-01 | 5.52E-01 | 2.00E-03** | 4.15E-01 |
| **InvertivoreS** | 1.46E-01 | 1.22E-01 | 2.76E-01 | 1.26E-01 | 1.68E-01 | 4.40E-01 | 2.26E-01 | 7.44E-01 |  | 5.68E-01 | 4.33E-01 | 5.20E-02 | 3.72E-01 |
| **Nectarivore** | 4.95E-01 | 3.59E-01 | 5.33E-01 | 5.61E-01 | 7.15E-01 | 7.88E-01 | 4.90E-01 | 5.75E-01 | 5.68E-01 |  | 6.53E-01 | 1.77E-01 | 8.64E-01 |
| **Piscivore** | 2.10E-02* | 1.52E-01 | 2.35E-01 | 1.20E-02* | 2.71E-01 | 7.70E-01 | 4.86E-01 | 5.52E-01 | 4.33E-01 | 6.53E-01 |  | 2.00E-03** | 5.41E-01 |
| **Scavenger** | 1.90E-02* | 4.79E-01 | 3.88E-01 | 3.00E-03** | 1.59E-01 | 2.00E-02 | 2.00E-03** | 2.00E-03** | 5.20E-02 | 1.77E-01 | 2.00E-03** |  | 4.30E-02* |
| **Tetra Hunt** | 1.45E-01 | 2.21E-01 | 3.32E-01 | 1.09E-01 | 3.47E-01 | 5.12E-01 | 2.97E-01 | 4.15E-01 | 3.72E-01 | 8.64E-01 | 5.41E-01 | 4.30E-02* |  |

## Table S6

Sensitivity analysis of the position of the quadrate on predicting longipterygid diet (See reconstructions in Figure S8). Posterior probabilities are provided from LDA on MA and functional index data from avian upper jaws, with semi-specialists excluded. Values with green backgrounds are more likely, values with red backgrounds are less likely. Compared to results with most likely reconstructions (Table 5), folivory is more likely when the quadrate is shifted anteriorly and scavenging and piscivory are more likely when the quadrate is shifted posteriorly. The overall trend of invertivory and generalist feeding being likely across taxa persists, so we consider the MA and function index analysis robust to the reconstructed position of the quadrate. lg teeth and sm teeth *Longipteryx* refer to the large-toothed and small-toothed morphotypes of *Longipteryx*, respectively. Diet abbreviations: FrugivoreH hard frugivore, FrugivoreS soft frugivore, GranivoreS swallowing granivore, GranivoreH husking granivore, InvertivoreH hard invertivore, InvertivoreM medium invertivore, InvertivoreS soft invertivore, Tetra Hunt tetrapod hunter.

|  | **Taxon** | **Foli-vore** | **Frugi-voreH** | **Frugi-voreS** | **General-ist** | **Grani-voreH** | **Grani-voreS** | **Inverti-voreH** | **Inverti-voreM** | **Inverti-voreS** | **Nectari-vore** | **Pisci-vore** | **Scavenger** | **Tetra Hunt** |
| --- | --- | --- | --- | --- | --- | --- | --- | --- | --- | --- | --- | --- | --- | --- |
| **Anterior- Shifted**  **Quadrate** | lg teeth *Longipteryx* | 5.66E-08 | 6.15E-03 | 3.66E-06 | 1.12E-01 | 7.01E-08 | 2.97E-04 | 4.41E-01 | 3.32E-01 | 4.27E-03 | 2.94E-06 | 6.00E-02 | 4.37E-02 | 2.92E-04 |
|  | sm teeth *Longipteryx* | 4.94E-07 | 3.85E-03 | 4.08E-06 | 1.86E-01 | 6.57E-07 | 8.70E-04 | 4.40E-01 | 2.29E-01 | 2.78E-03 | 5.99E-06 | 8.83E-02 | 4.93E-02 | 2.07E-04 |
|  | *Longirostravis* | 9.61E-01 | 2.55E-10 | 1.21E-05 | 2.71E-02 | 2.73E-08 | 1.35E-03 | 1.22E-03 | 5.75E-03 | 6.96E-06 | 2.66E-04 | 5.34E-05 | 2.82E-07 | 2.97E-03 |
|  | *Rapaxavis* | 7.61E-04 | 1.51E-06 | 2.01E-05 | 3.14E-01 | 1.65E-08 | 2.71E-03 | 1.33E-01 | 5.17E-01 | 7.98E-04 | 7.48E-05 | 9.85E-03 | 6.56E-04 | 2.09E-02 |
|  | *Shanweiniao* | 8.60E-05 | 1.35E-05 | 3.13E-06 | 4.28E-01 | 2.85E-07 | 4.17E-03 | 1.75E-01 | 3.01E-01 | 2.93E-04 | 2.12E-05 | 5.55E-02 | 1.77E-02 | 1.87E-02 |
| **Posterior- Shifted**  **Quadrate** | lg teeth *Longipteryx* | 5.71E-09 | 1.78E-03 | 2.68E-07 | 8.16E-02 | 1.68E-08 | 2.64E-04 | 2.73E-01 | 3.05E-01 | 1.06E-03 | 2.04E-07 | 4.49E-02 | 2.92E-01 | 3.59E-04 |
|  | sm teeth *Longipteryx* | 1.60E-08 | 1.39E-03 | 2.27E-07 | 9.59E-02 | 1.07E-07 | 5.43E-04 | 2.50E-01 | 1.78E-01 | 6.93E-04 | 2.54E-07 | 6.28E-02 | 4.11E-01 | 1.82E-04 |
|  | *Longirostravis* | 1.43E-07 | 5.76E-05 | 1.40E-07 | 9.85E-02 | 3.84E-07 | 1.45E-03 | 1.22E-01 | 6.84E-02 | 9.46E-05 | 6.59E-07 | 2.05E-01 | 5.03E-01 | 1.97E-03 |
|  | *Rapaxavis* | 1.81E-10 | 1.77E-04 | 2.66E-09 | 1.94E-02 | 3.68E-09 | 5.81E-05 | 5.31E-02 | 4.33E-02 | 2.83E-05 | 6.06E-09 | 8.98E-02 | 7.94E-01 | 2.05E-04 |
|  | *Shanweiniao* | 7.31E-09 | 2.24E-04 | 2.45E-08 | 5.67E-02 | 7.54E-08 | 3.09E-04 | 8.94E-02 | 5.97E-02 | 5.67E-05 | 8.98E-08 | 1.33E-01 | 6.60E-01 | 7.69E-04 |

## Table S7

Quantified differences MWAM strain from the sensitivity analysis in Figure S9. Each comparison is made to the radiograph model (representing the true rhamphotheca thickness), showing differences for each taxon and each modelled thickness as well as total difference across all taxa for each modelled thickness. Note that the 80% bone 20% rhamphotheca models (used for unknown thicknesses in this study) have the least different MWAM strain from the radiograph models overall.

|  | ***Aceros*** | ***Bubo*** | ***Buteo*** | ***Colinus*** | ***Nymphicus*** | **Total** |
| --- | --- | --- | --- | --- | --- | --- |
| **20% Bone 80% Rhamphotheca** | 1.37E-05 | 3.94E-06 | 5.08E-06 | 2.11E-07 | 1.57E-06 | 2.30E-05 |
| **50% Bone 50% Rhamphotheca** | 5.57E-06 | 8.46E-07 | 2.81E-06 | 6.57E-07 | 9.29E-08 | 9.88E-06 |
| **80% Bone 20% Rhamphotheca** | 1.60E-06 | 3.31E-06 | 4.56E-09 | 7.17E-07 | 1.85E-06 | 5.64E-06 |
| **100% Bone** | 9.26E-08 | 7.85E-06 | 1.17E-06 | 3.27E-06 | 3.70E-06 | 1.24E-05 |
